# Supplementary material for: The origin of heterogeneous nanoparticle uptake by cells
Source: Nat Commun. 2019 May 28;10:2341. doi: 10.1038/s41467-019-10112-4 (PMC6538724; doi:10.1038/s41467-019-10112-4)
Supplement: Supplementary file 1 — Supplementary Information [file 41467_2019_10112_MOESM1_ESM.pdf]

Supplementary Information

The origin of heterogeneous nanoparticle uptake by cells

Paul Rees*, John W. Wills, M. Rowan Brown, Claire M. Barnes and Huw D. Summers.

List of supplementary information –

1. **Supplementary Table 1** – Tabulated physico-chemical characteristics of the Qtracker 705 quantum dot nanoparticles used in this study.

2. **Supplementary Figures 1-4** – Further confocal micrographs of the A549 and BEAS-2B cells showing nanoparticle uptake across a 16-fold variation in the dose-time product. Segmentation of cell nuclei, cell membranes and nanoparticle loaded vesicles is demonstrated. These figures also include negative control data and exact N sizes for both the cells and NLV quantified in each experiment.

3. **Supplementary Figures 5/6** – These figures show the use of a gamma function to describe the distribution of cell areas across populations of cells. The shape and scale factors used for each fit are provided.

4. **Supplementary Figure 7** – This figure presents an additional live cell analysis showing that far greater numbers of unloaded vesicles form during the one hour exposure period used herein when compared to the number of nanoparticle loaded vesicles (NLVs) that form in the same period.

5. **Supplementary Figure 8** – Further histograms and fits showing that the nanoparticle dose per vesicle is independent from the initial administered dose. Data are presented for both the A549 and BEAS-2B cell lines across the full 16-fold range of the dose-time product.

6. **Supplementary Figure 9** – Further examples of experimental measurements and theoretical predictions of nanoparticle cellular delivery. Results are provided for both NLV numbers per cell and for total nanoparticle fluorescence per cell.

7. **Supplementary Note 1–** This section presents via screenshots the complete CellProfiler image analysis pipeline used to segment and measure the properties of each cell's nucleus, cell membrane and any associated nanoparticle loaded vesicles.

8. **Supplementary Note 2 –** Model parameters and fitting instructions. This section contains a complete list and description of all model parameters alongside dedicated instructions for fitting the nanoparticle uptake model derived herein.

Supplementary Table 1 – Nanoparticle physico-chemical characteristics

Product name	Text reference	Primary size	Shape / morphology	Composition	Crystallinity	Surface charge / chemistry
Thermo-Fisher Qtracker™ 705 Cell Labelling Kit (Q25061MP)	Quantum dot; nanoparticle	~ 10-20 nm	Oval / smooth	CdTe/ZnS (cadmium telluride with a zinc sulphide shell)	Crystalline CdTe core	Cationic, surface functionalised with polyarginine peptides

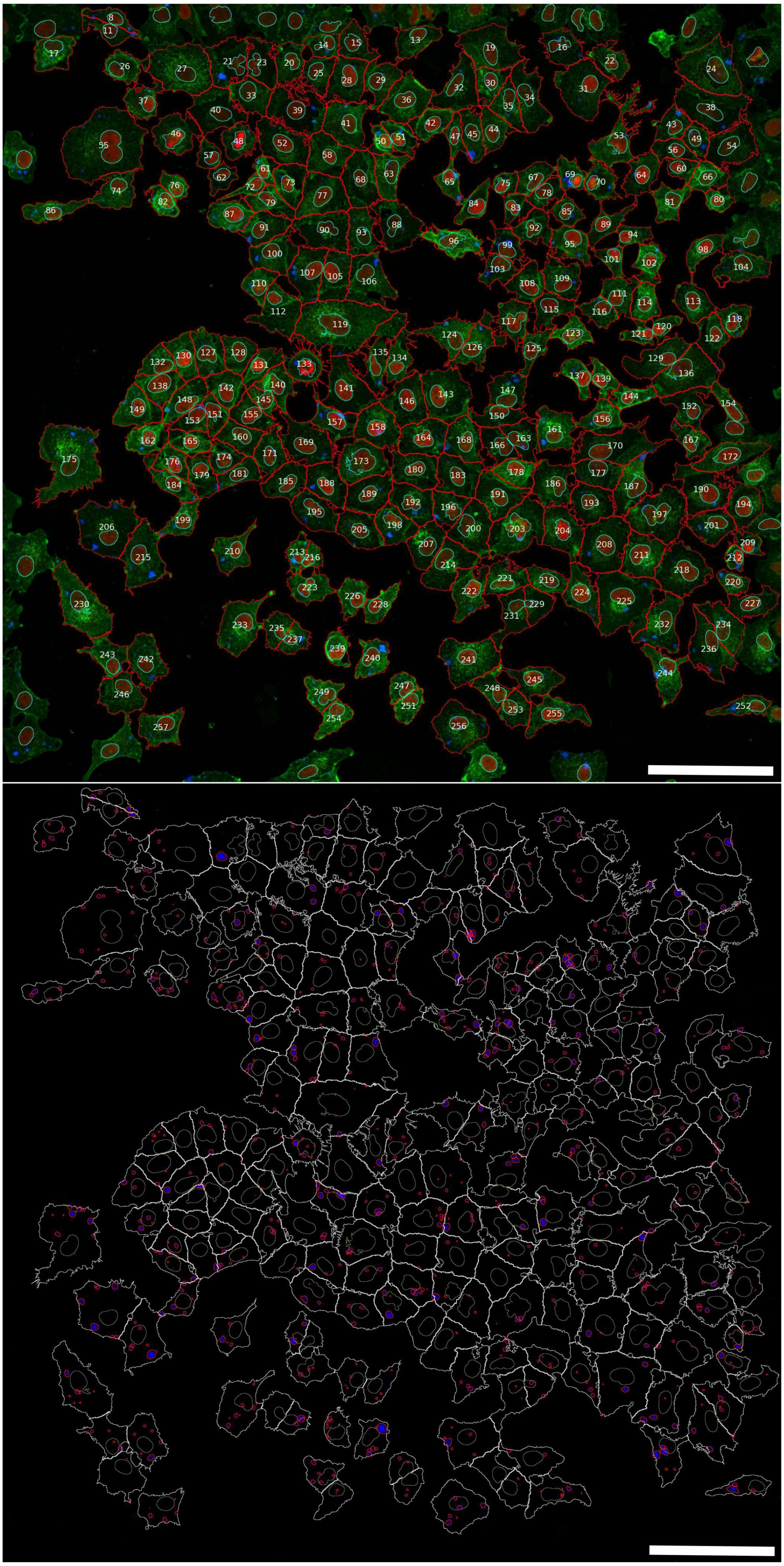

Supplementary Figure 1 – Image based analysis of nanoparticle delivery to A459 lung adenocarcinoma cells. (Top) A typical field of view taken from over 100 collected per experiment by laser scanning confocal microscopy. Cell identification numbers alongside nuclear and cell membrane segmentation masks achieved by image analysis (see Methods) are shown in blue and red, respectively. (Bottom) For each cell (segmentation outlines shown), individual nanoparticle loaded vesicles (NLVs) were also segmented (red outlines). In this way, image analysis allowed nuclear, cell and NLV features (*e.g.,* size, shape and fluorescence intensity *etc.*) to be measured for ~>10^4 cells and ~>10^5 NLV for each exposure condition (*i.e.,* dose-time combination). (Scale bars = 100 μm).

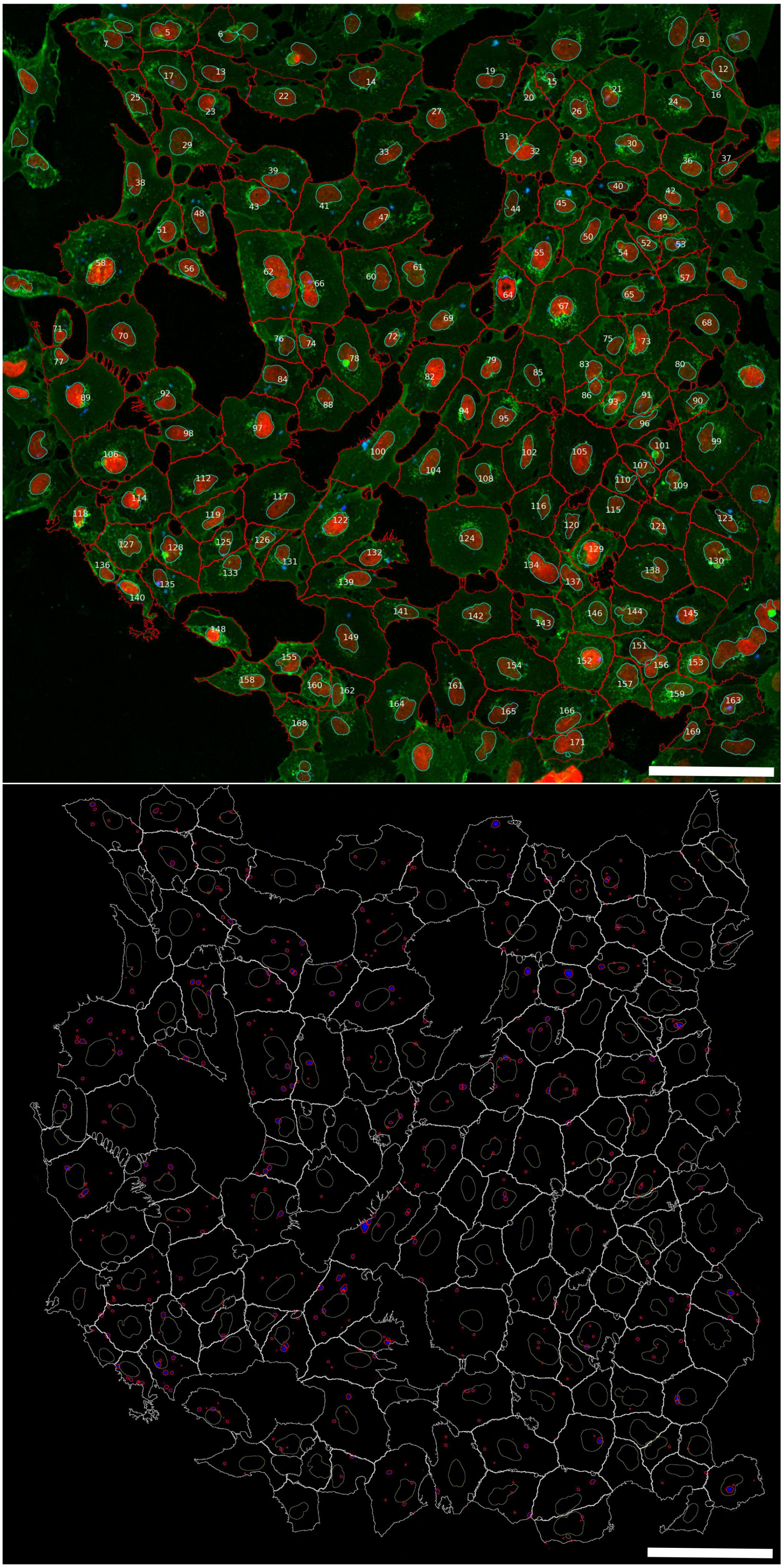

Supplementary Figure 2 – Image based analysis of nanoparticle delivery to BEAS-2B bronchial epithelial cells. (Top) A typical field of view taken from over 100 collected per experiment by laser scanning confocal microscopy. Cell identification numbers alongside nuclear and cell membrane segmentation masks achieved by image analysis (see Methods) are shown in blue and red, respectively. (Bottom) For each cell (segmentation outlines shown), individual nanoparticle loaded vesicles (NLVs) were also segmented (red outlines). In this way, image analysis allowed nuclear, cell and NLV features (*e.g.*, size, shape and fluorescence intensity *etc.*) to be measured for ~>10^4 cells and ~>10^5 NLV for each exposure condition (*i.e.*, dose-time combination). (Scale bars = 100 µm).

A549 Cells

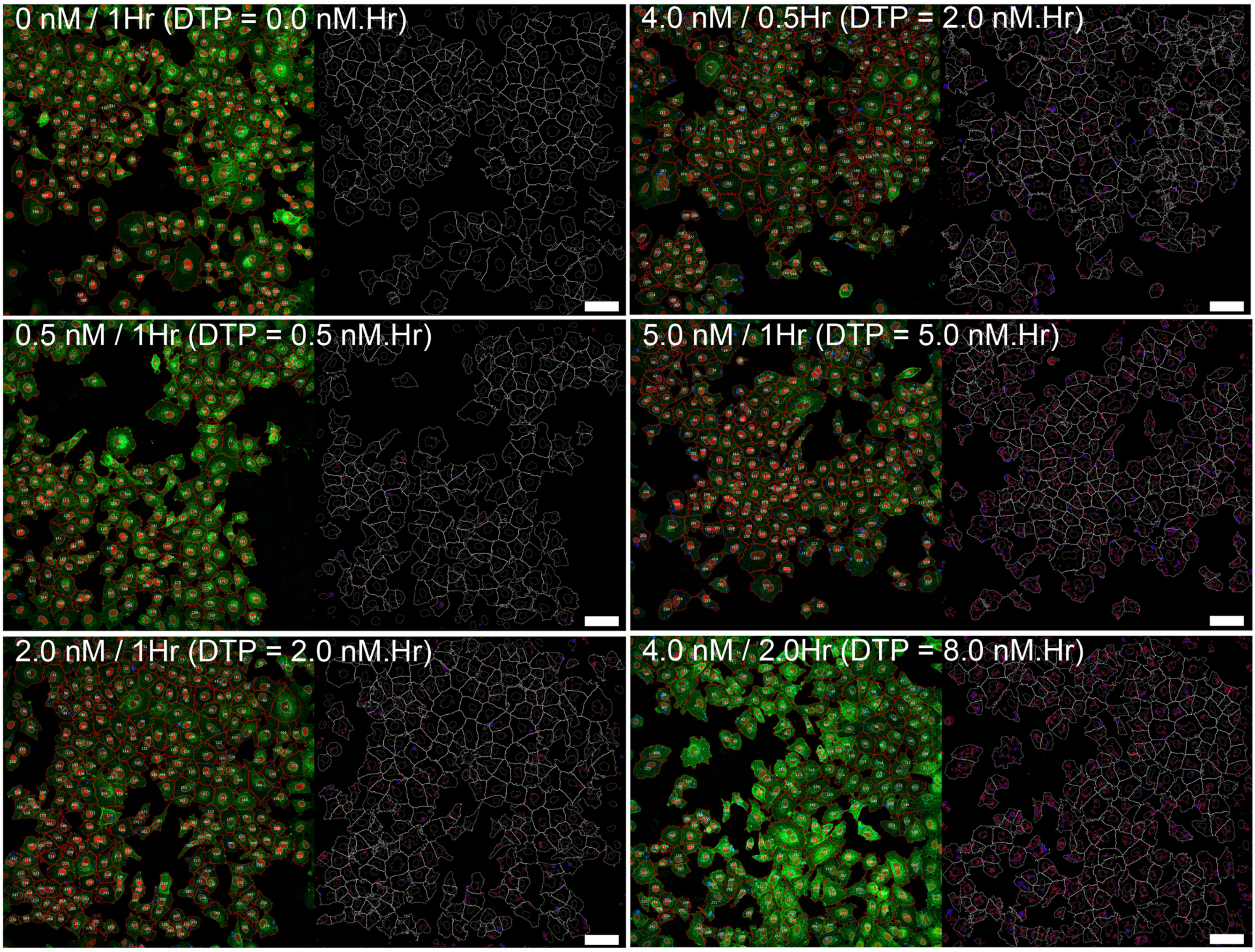

A549 Cells

Dose (nM)	Exposure duration (Hr)	Dose-time product (nM.Hr)	Replicates	n_{cell}	n_{nlv}
0.0	1.0	0.0	1	384	0
0.5	1.0	0.5	1	10,811	16,133
2.0	1.0	2.0	1	14,442	65,749
4.0	0.5	2.0	1	14,488	85,135
5.0	1.0	5.0	1	10,327	142,188
4.0	2.0	8.0	1	13,464	221,115

Supplementary Figure 3 – Image analysis of A549 bronchial adenocarcinoma cells. (**Left**) Typical confocal micrographs showing cells stained with Hoechst 33342 (nuclei, red) and wheat germ agglutinin-AlexaFluor 555 conjugate (cell membranes, green). The cells were exposed to 0.0-5.0 nM doses of 8 nm Qtracker® 705 quantum dot nanoparticles (blue) for 0-2 hours. (Overlaid / **Right**) CellProfiler was used to segment the nucleus, cell membrane and any nanoparticle loaded vesicles (NLV) in each cell object. The manual threshold set for the NLV channel – subsequently used for determining the number of NLV per cell - was set against an image stack treated identically but unexposed to Qtracker® 705 (i.e. a negative control, Top Left image). (**Bottom**) Tabulated n sizes showing the number of cells and number of NLV quantified for each dose/exposure condition. Scale bars = 100 µm.

BEAS-2B Cells

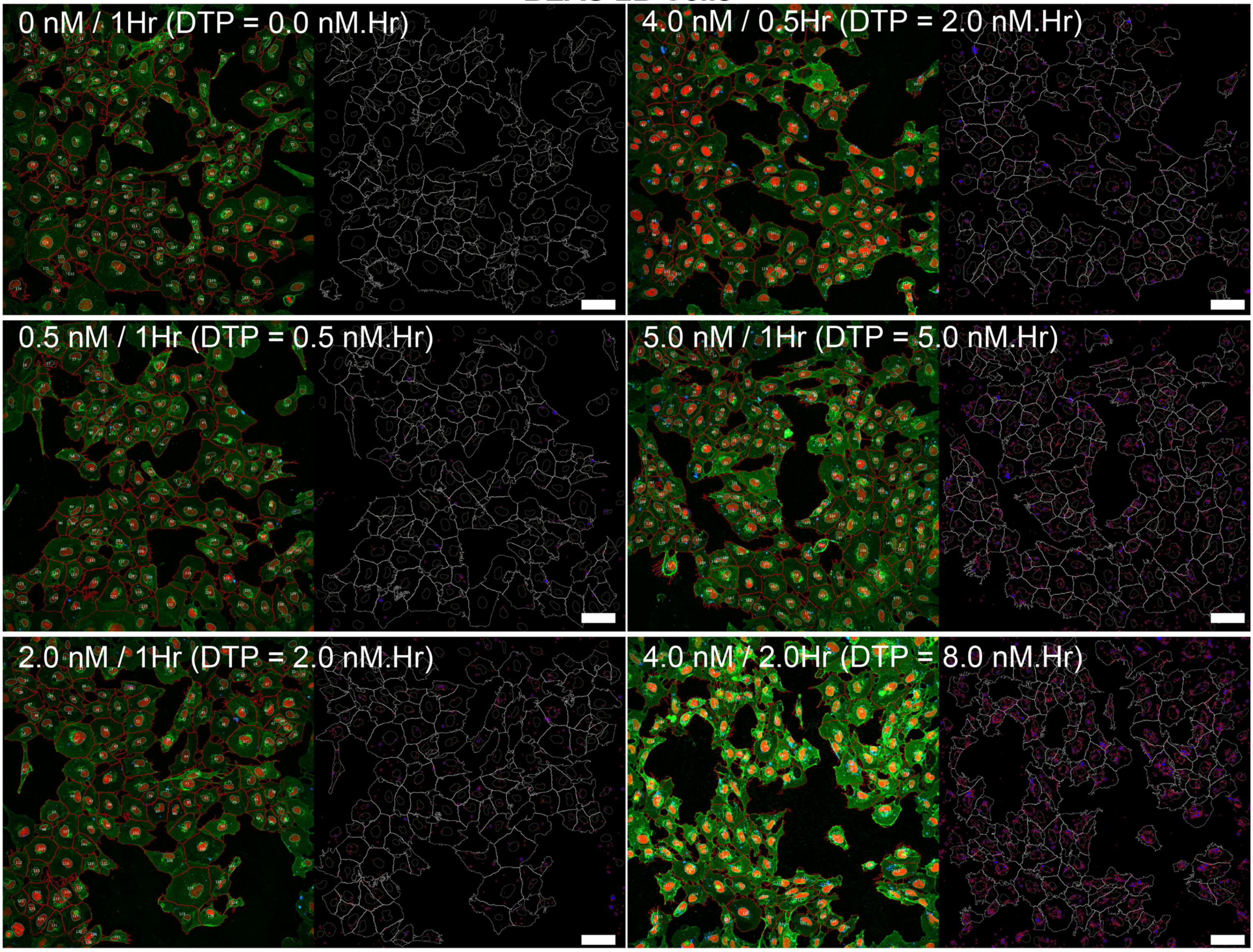

BEAS-2B Cells

Dose (nM)	Exposure duration (Hr)	Dose-time product (nM.Hr)	Replicates	n_{cell}	n_{nlv}
0.0	1.0	0.0	1	412	0
0.5	1.0	0.5	1	16,134	12,040
2.0	1.0	2.0	1	14,003	58,432
4.0	0.5	2.0	1	14,488	85,135
5.0	1.0	5.0	1	14,589	153,410
4.0	2.0	8.0	1	18,523	287,773

Supplementary Figure 4 – Image analysis of BEAS-2B lung epithelial cells. (**Left**) Typical confocal micrographs showing cells stained with Hoechst 33342 (nuclei, red) and wheat germ agglutinin-AlexaFluor 555 conjugate (cell membranes, green). The cells were exposed to 0.0-5.0 nM doses of 8 nm Qtracker® 705 quantum dot nanoparticles (blue) for 0-2 hours. (Overlaid / **Right**) CellProfiler was used to segment the nucleus, cell membrane and any nanoparticle loaded vesicles (NLV) in each cell object. The manual threshold set for the NLV channel – subsequently used for determining the number of NLV per cell - was set against an image stack treated identically but unexposed to Qtracker® 705 (i.e. a negative control, Top Left image). (**Bottom**) Tabulated n sizes showing the number of cells and number of NLV quantified for each dose/exposure condition. Scale bars = 100 µm.

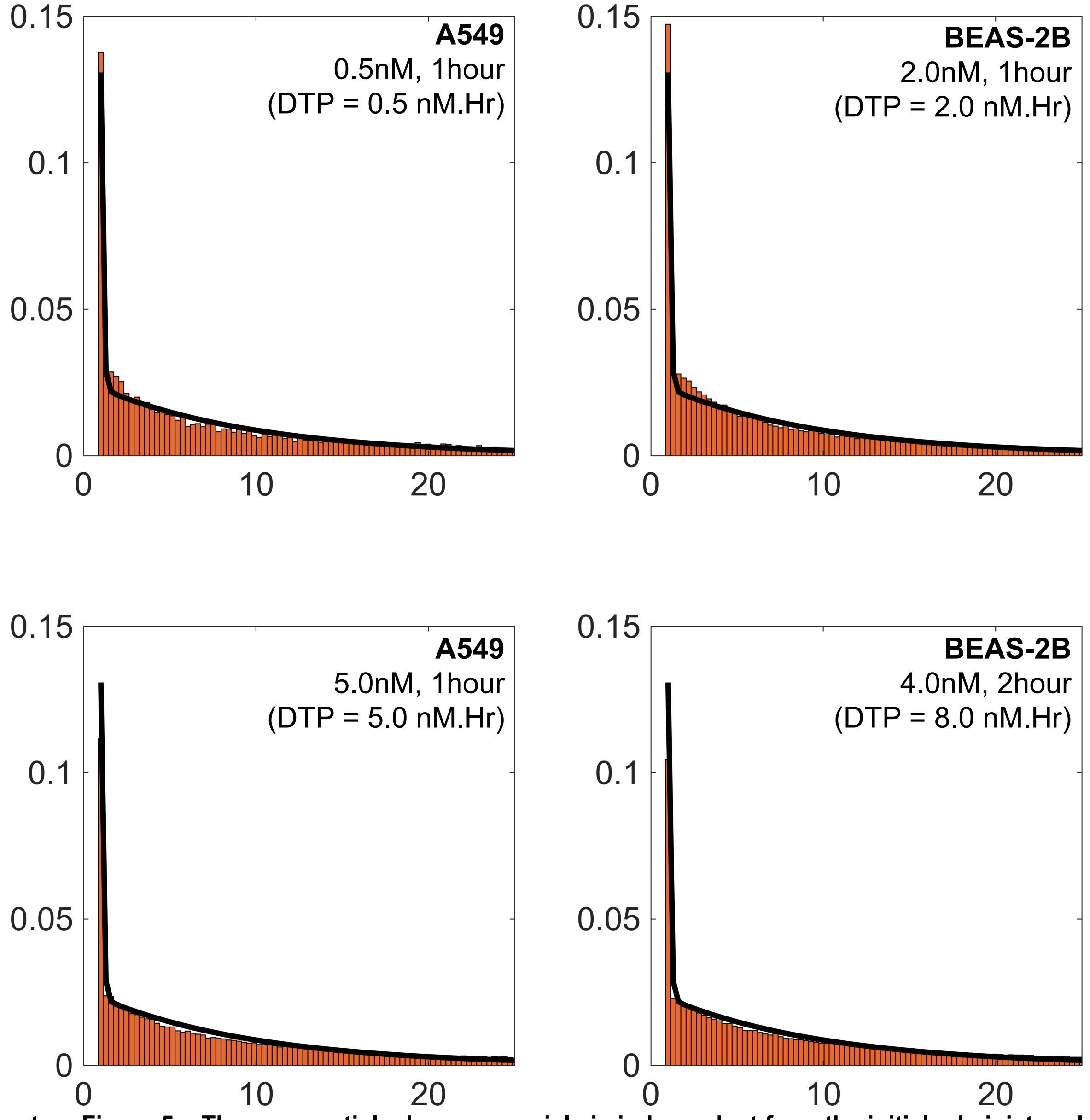

Supplementary Figure 5 – The nanoparticle dose per vesicle is independent from the initial administered dose. Plots showing four further histograms (*i.e.,* in addition to those shown in Figure 2g) from both cell lines and across the full 16-fold range of the dose/time product. The black lines represent the exact same, simultaneous fits used in Figure 2g - demonstrating the insensitivity of the nanoparticle dose per vesicle to the initial administered dose or exposure duration.

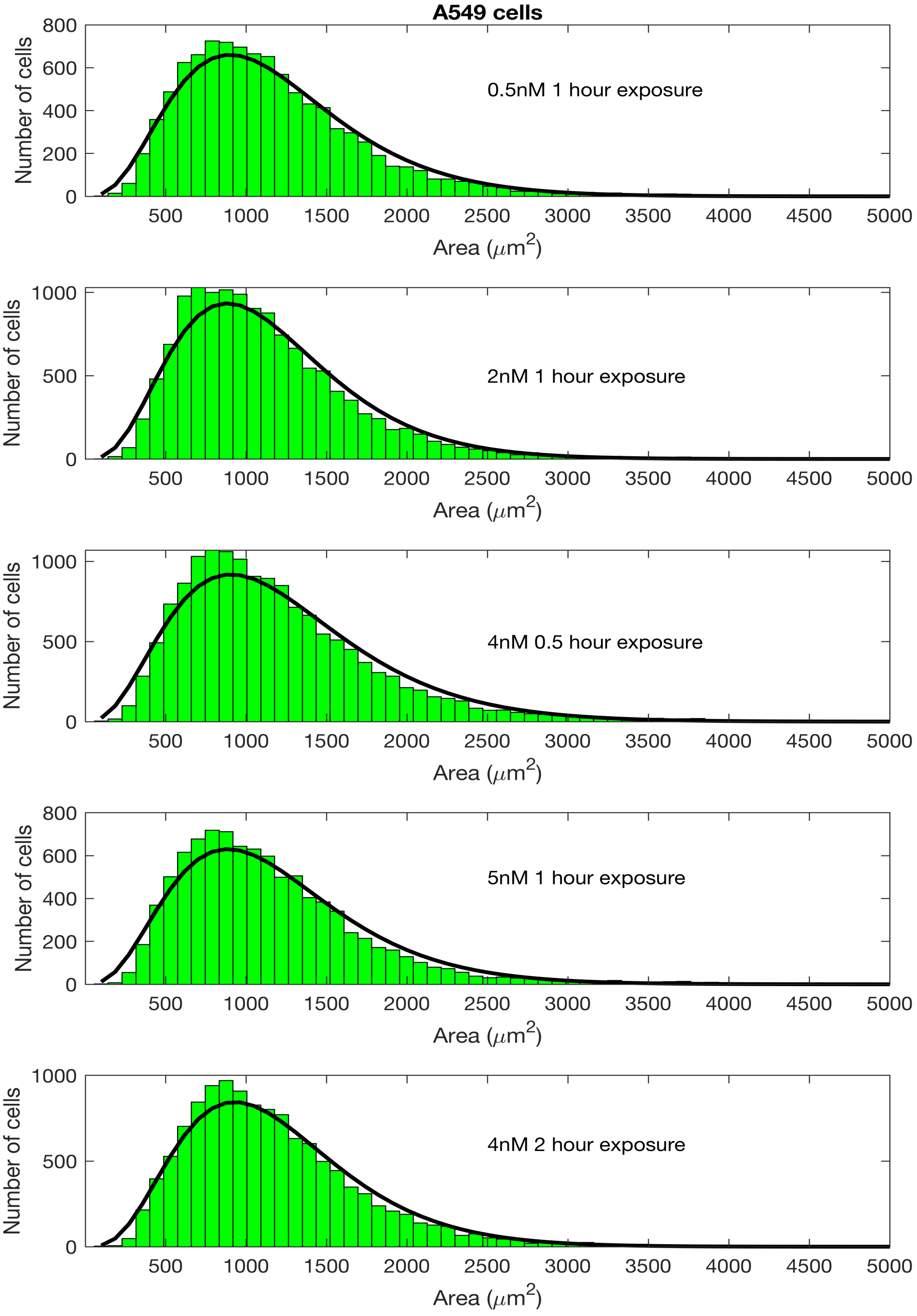

A549 Cells

Dose (nM)	Exposure duration (Hr)	Dose-time product (nM.Hr)	Scale factor (beta)	Shape factor (alpha)
0.5	1.0	0.5	4.2299	278.23
2.0	1.0	2.0	4.4254	257.52
4.0	0.5	2.0	3.8449	318.28
5.0	1.0	5.0	4.1016	285.74
4.0	2.0	8.0	4.5637	260.54
**Mean (+/- std)**			**4.2331 (0.28029)**	**280.06 (24.42)**

Supplementary Figure 6 – The distribution of A549 cell areas is well approximated by a gamma distribution function. Bars show the distribution of cell areas across the population for each dose and exposure duration as measured by image analysis. The inset table shows the shape (alpha) and scale (beta) factors used to fit the gamma function to the measured data (overlaid black curves). The cell area distributions were unperturbed by the different doses/exposure durations employed (0.5 – 16 nM.Hr).

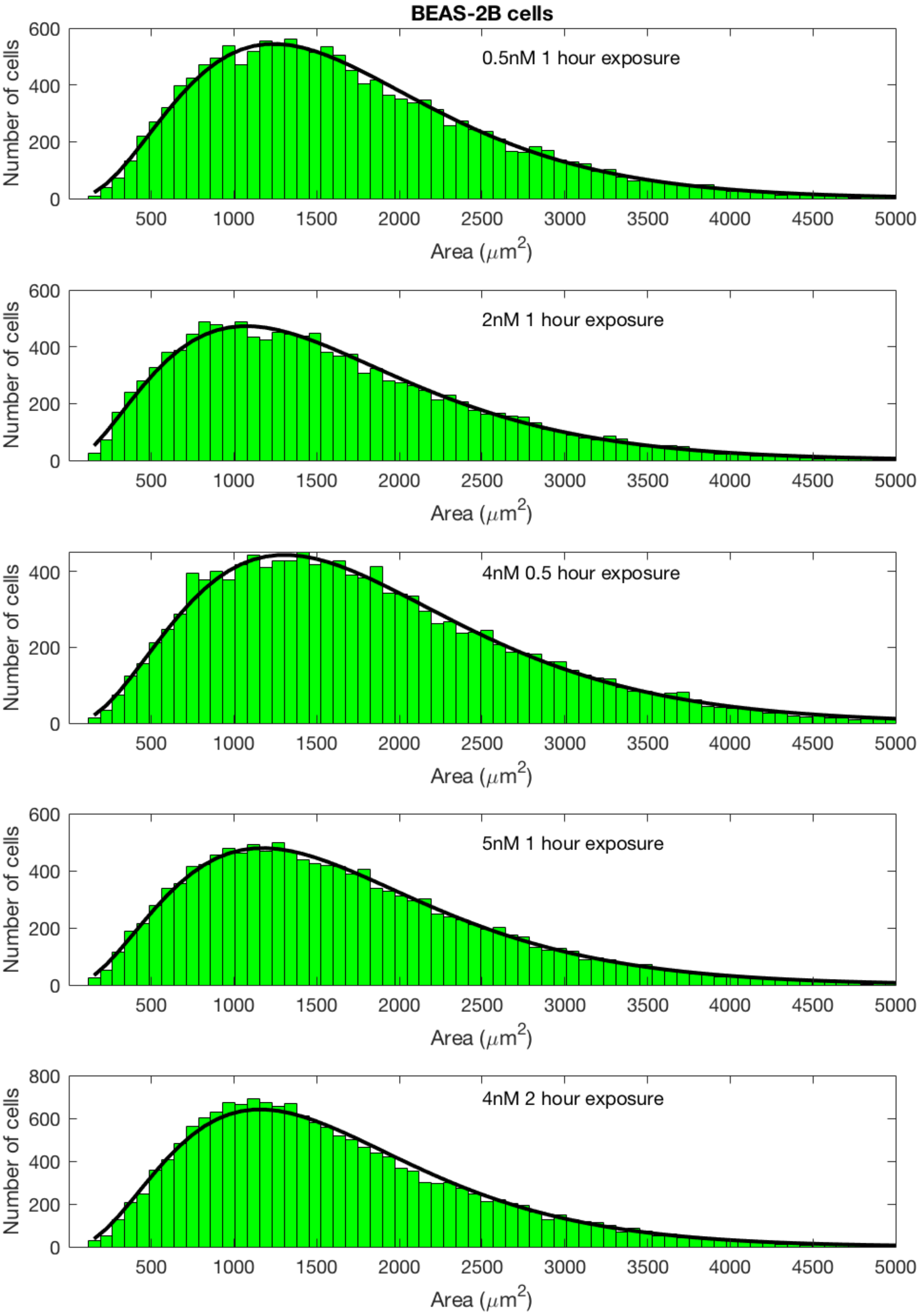

BEAS-2B Cells

Dose (nM)	Exposure duration (Hr)	Dose-time product (nM.Hr)	Scale factor (beta)	Shape factor (alpha)
0.5	1.0	0.5	3.6889	460.91
2.0	1.0	2.0	3.0406	526.02
4.0	0.5	2.0	3.4441	534.15
5.0	1.0	5.0	3.2882	512.48
4.0	2.0	8.0	3.4759	467.42
**Mean (+/- std)**			**3.3875 (0.24085)**	**500.2 (33.87)**

Supplementary Figure 7 – The distribution of BEAS-2B cell areas is well approximated by a gamma distribution function. Bars show the distribution of cell areas across the population for each dose and exposure duration as measured by image analysis. The inset table shows the shape (alpha) and scale (beta) factors used to fit the gamma function to the measured data (overlaid black curves). The cell area distributions were unperturbed by the different doses/exposure durations employed (0.5 – 16 nM.Hr).

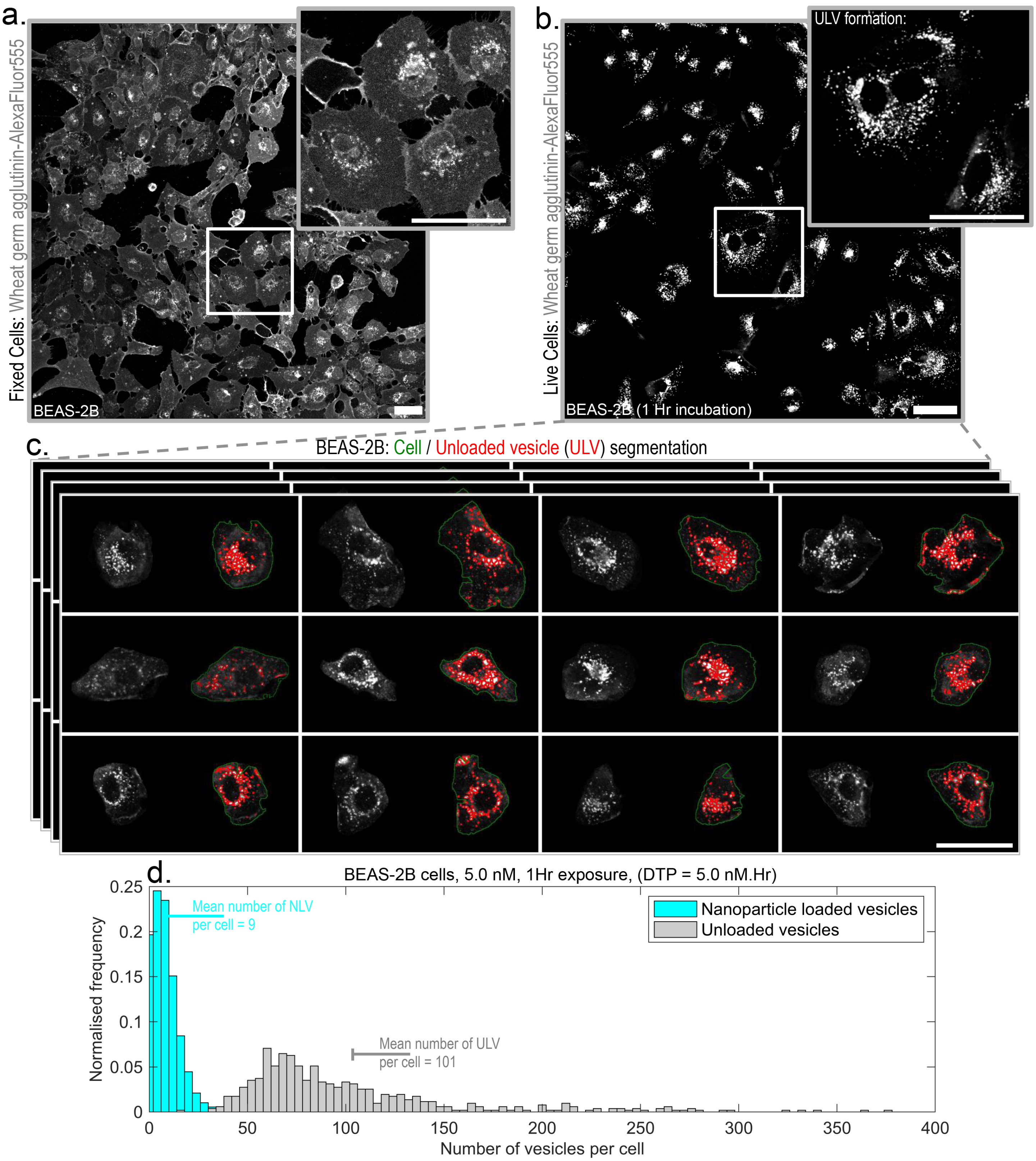

Supplementary Figure 8 – For typical nanomolar dose ranges, nanoparticle capture by forming endosomes is a rare event. a, Labelling fixed cells with fluorescently conjugated wheat germ agglutinin (WGA) provides an excellent outline of the cell membrane. In contrast (**b**), live cells incubated with the dye before washing and returned to the incubator for 1 hour rapidly loose their outline and instead show highly punctate staining as the labelled membrane is 'turned over' into endosomes. **c**, Due to the loss of a clear cell outline, cells in these images were manually segmented (n_{cell} = 507) prior to automated segmentation of the bright detail intensity foci (i.e., unloaded vesicles) in each (red outlines). This approach permits estimation of the number of unloaded vesicles (ULV) formed in the hour period (N.B., due to resolution limits, the count is likely conservative). **d**, None-the-less, it can be seen than the number of ULVs formed is *much* greater than the number of NLVs formed – even for the highest nanoparticle exposure (5 nM) used in this study for the 1 hour exposure duration. Scale bars = 50 μm.

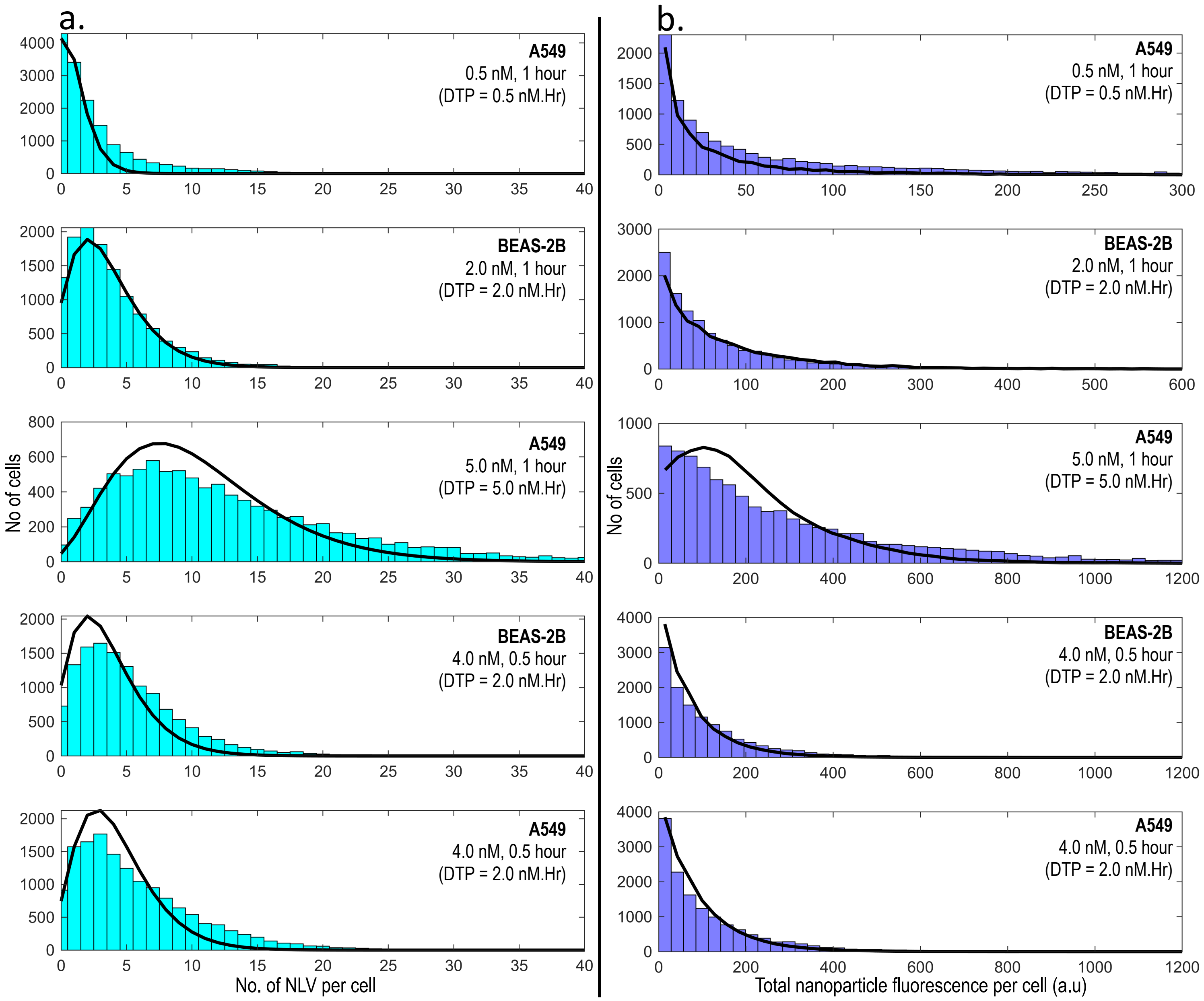

Supplementary Figure 9 – Further experimental measurements and theoretical predictions of nanoparticle cellular delivery. a, Histograms showing the number of NLV formed per cell across the cell population for both BEAS-2B and A549 cells under different exposure conditions (DTP = dose/time product). The black curves are the predicted NLV distributions (see Methods, Equation 12) using the value of λ=0.00107nM^{-1}hr^{-1}m^{-2} for BEAS-2B cells and λ=0.00135nM^{-1}hr^{-1}m^{-2} for A549 cells. **b**, Histograms of total nanoparticle fluorescence per cell across the cell population for the matched exposure conditions as in (**a**). The black curves are predicted cell intensity distributions (see Methods, Equation 13).

Supplementary Note 1 – This section presents screenshots of the complete CellProfiler image analysis pipeline used to segment and measure the properties of each cell's nucleus, cell membrane and to count and measure the intensity of any associated nanoparticle loaded vesicles. The **Supplementary Data 1** file further provides the CellProfiler pipeline template file, as well as example .lsm images from the microscope. To use the image analysis pipeline with new image data, the 'IdentifyPrimaryObjects' modules simply need adjusting so that the 'typical diameter of objects' size range matches the pixel scaling of the new images. For complete newcomers, we first recommend downloading the sample image data and pipeline from the **Supplementary Data 1** file, which will allow the pipeline to run with a subset of our data and will allow the user to see how each module works. To repurpose the pipeline with new data that's in a different channel format, or to write a new pipeline to perform a similar task with different data, we recommend visiting the video tutorials at https://cellprofiler.org/tutorials/ to pick up the process.

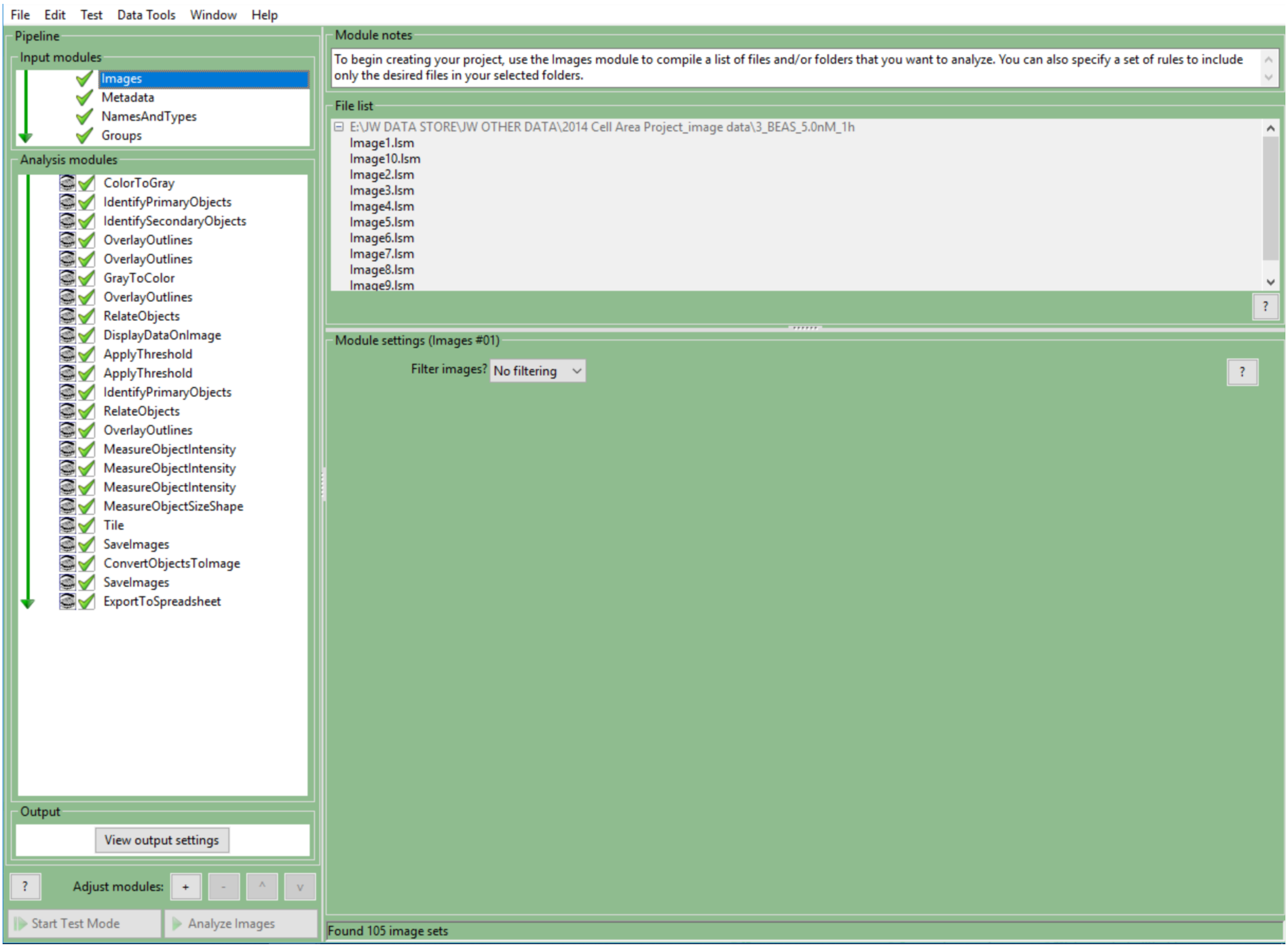

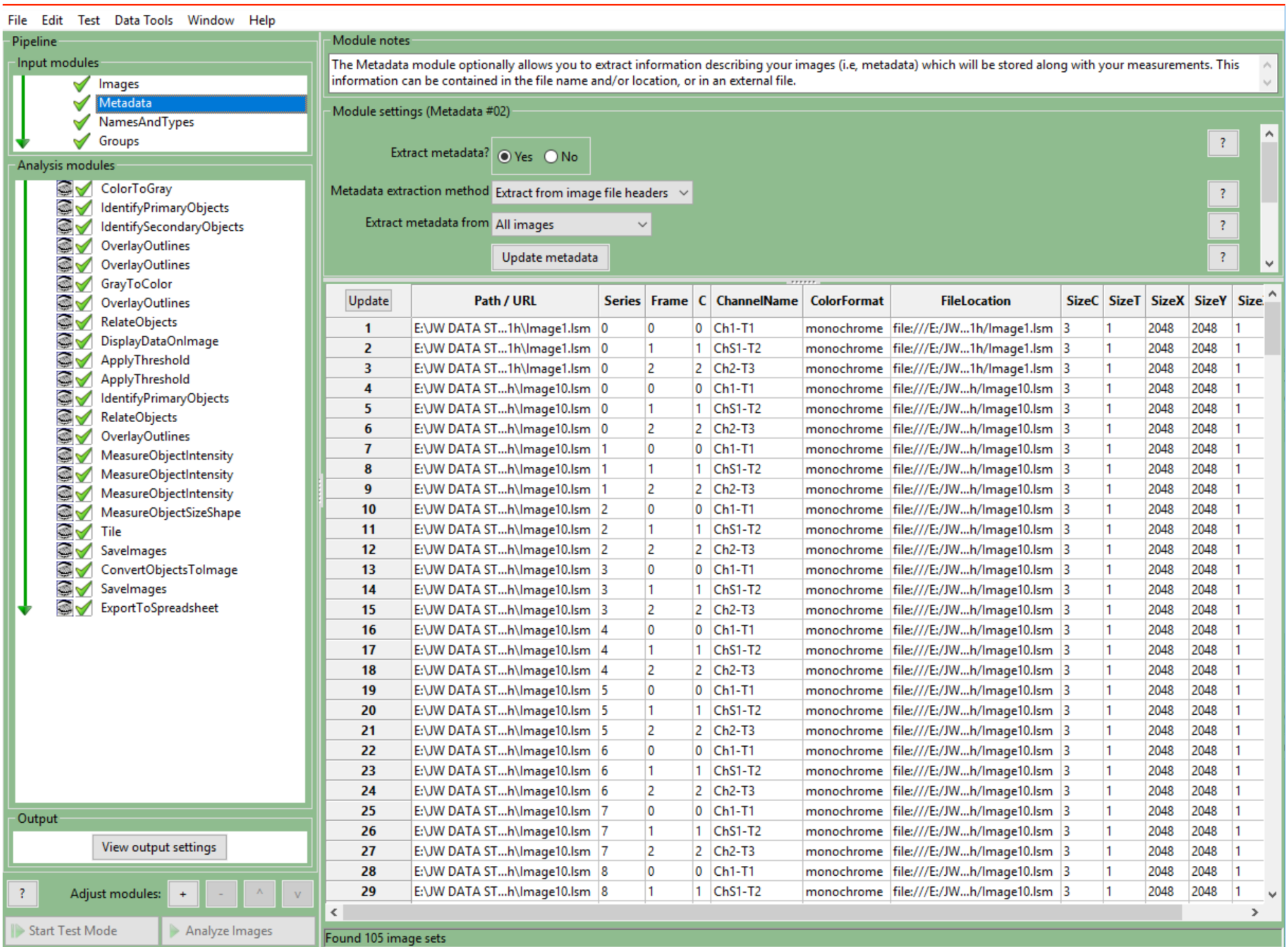
File Edit Test Data Tools Window Help
Pipeline
Input modules
Images
Metadata
NamesAndTypes
Groups
Analysis modules
ColorToGray
IdentifyPrimaryObjects
IdentifySecondaryObjects
OverlayOutlines
OverlayOutlines
GrayToColor
OverlayOutlines
RelateObjects
DisplayDataOnImage
ApplyThreshold
ApplyThreshold
IdentifyPrimaryObjects
RelateObjects
OverlayOutlines
MeasureObjectIntensity
MeasureObjectIntensity
MeasureObjectIntensity
MeasureObjectSizeShape
Tile
SaveImages
ConvertObjectsToImage
SaveImages
ExportToSpreadsheet
Output
View output settings
?
Adjust modules:
+
-
^
v
Start Test Mode
Analyze Images
Module notes
The Metadata module optionally allows you to extract information describing your images (i.e, metadata) which will be stored along with your measurements. This information can be contained in the file name and/or location, or in an external file.
Module settings (Metadata #02)
Extract metadata? Yes No
Metadata extraction method Extract from image file headers
Extract metadata from All images
Update metadata
?
Update
Path / URL
Series
Frame
C
ChannelName
ColorFormat
FileLocation
SizeC
SizeT
SizeX
SizeY
Size
1 E:\JW DATA ST...1h\Image1.lsm 0 0 0 Ch1-T1 monochrome file:///E:/JW...1h/Image1.lsm 3 1 2048 2048 1
2 E:\JW DATA ST...1h\Image1.lsm 0 1 1 ChS1-T2 monochrome file:///E:/JW...1h/Image1.lsm 3 1 2048 2048 1
3 E:\JW DATA ST...1h\Image1.lsm 0 2 2 Ch2-T3 monochrome file:///E:/JW...1h/Image1.lsm 3 1 2048 2048 1
4 E:\JW DATA ST...h\Image10.lsm 0 0 0 Ch1-T1 monochrome file:///E:/JW...h/Image10.lsm 3 1 2048 2048 1
5 E:\JW DATA ST...h\Image10.lsm 0 1 1 ChS1-T2 monochrome file:///E:/JW...h/Image10.lsm 3 1 2048 2048 1
6 E:\JW DATA ST...h\Image10.lsm 0 2 2 Ch2-T3 monochrome file:///E:/JW...h/Image10.lsm 3 1 2048 2048 1
7 E:\JW DATA ST...h\Image10.lsm 1 0 0 Ch1-T1 monochrome file:///E:/JW...h/Image10.lsm 3 1 2048 2048 1
8 E:\JW DATA ST...h\Image10.lsm 1 1 1 ChS1-T2 monochrome file:///E:/JW...h/Image10.lsm 3 1 2048 2048 1
9 E:\JW DATA ST...h\Image10.lsm 1 2 2 Ch2-T3 monochrome file:///E:/JW...h/Image10.lsm 3 1 2048 2048 1
10 E:\JW DATA ST...h\Image10.lsm 2 0 0 Ch1-T1 monochrome file:///E:/JW...h/Image10.lsm 3 1 2048 2048 1
11 E:\JW DATA ST...h\Image10.lsm 2 1 1 ChS1-T2 monochrome file:///E:/JW...h/Image10.lsm 3 1 2048 2048 1
12 E:\JW DATA ST...h\Image10.lsm 2 2 2 Ch2-T3 monochrome file:///E:/JW...h/Image10.lsm 3 1 2048 2048 1
13 E:\JW DATA ST...h\Image10.lsm 3 0 0 Ch1-T1 monochrome file:///E:/JW...h/Image10.lsm 3 1 2048 2048 1
14 E:\JW DATA ST...h\Image10.lsm 3 1 1 ChS1-T2 monochrome file:///E:/JW...h/Image10.lsm 3 1 2048 2048 1
15 E:\JW DATA ST...h\Image10.lsm 3 2 2 Ch2-T3 monochrome file:///E:/JW...h/Image10.lsm 3 1 2048 2048 1
16 E:\JW DATA ST...h\Image10.lsm 4 0 0 Ch1-T1 monochrome file:///E:/JW...h/Image10.lsm 3 1 2048 2048 1
17 E:\JW DATA ST...h\Image10.lsm 4 1 1 ChS1-T2 monochrome file:///E:/JW...h/Image10.lsm 3 1 2048 2048 1
18 E:\JW DATA ST...h\Image10.lsm 4 2 2 Ch2-T3 monochrome file:///E:/JW...h/Image10.lsm 3 1 2048 2048 1
19 E:\JW DATA ST...h\Image10.lsm 5 0 0 Ch1-T1 monochrome file:///E:/JW...h/Image10.lsm 3 1 2048 2048 1
20 E:\JW DATA ST...h\Image10.lsm 5 1 1 ChS1-T2 monochrome file:///E:/JW...h/Image10.lsm 3 1 2048 2048 1
21 E:\JW DATA ST...h\Image10.lsm 5 2 2 Ch2-T3 monochrome file:///E:/JW...h/Image10.lsm 3 1 2048 2048 1
22 E:\JW DATA ST...h\Image10.lsm 6 0 0 Ch1-T1 monochrome file:///E:/JW...h/Image10.lsm 3 1 2048 2048 1
23 E:\JW DATA ST...h\Image10.lsm 6 1 1 ChS1-T2 monochrome file:///E:/JW...h/Image10.lsm 3 1 2048 2048 1
24 E:\JW DATA ST...h\Image10.lsm 6 2 2 Ch2-T3 monochrome file:///E:/JW...h/Image10.lsm 3 1 2048 2048 1
25 E:\JW DATA ST...h\Image10.lsm 7 0 0 Ch1-T1 monochrome file:///E:/JW...h/Image10.lsm 3 1 2048 2048 1
26 E:\JW DATA ST...h\Image10.lsm 7 1 1 ChS1-T2 monochrome file:///E:/JW...h/Image10.lsm 3 1 2048 2048 1
27 E:\JW DATA ST...h\Image10.lsm 7 2 2 Ch2-T3 monochrome file:///E:/JW...h/Image10.lsm 3 1 2048 2048 1
28 E:\JW DATA ST...h\Image10.lsm 8 0 0 Ch1-T1 monochrome file:///E:/JW...h/Image10.lsm 3 1 2048 2048 1
29 E:\JW DATA ST...h\Image10.lsm 8 1 1 ChS1-T2 monochrome file:///E:/JW...h/Image10.lsm 3 1 2048 2048 1
Found 105 image sets

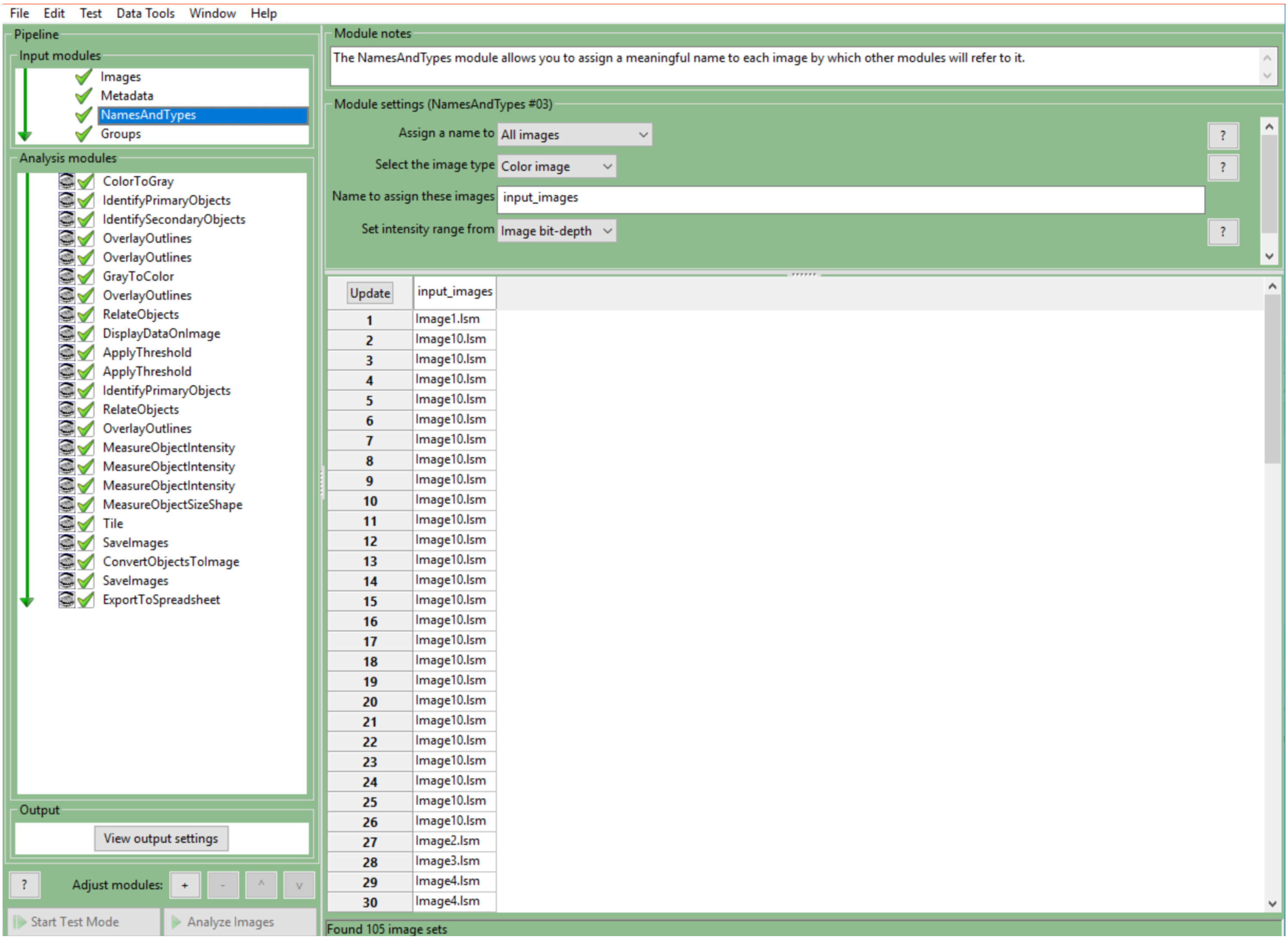
File Edit Test Data Tools Window Help
Pipeline
Input modules
Images
Metadata
NamesAndTypes
Groups
Analysis modules
ColorToGray
IdentifyPrimaryObjects
IdentifySecondaryObjects
OverlayOutlines
OverlayOutlines
GrayToColor
OverlayOutlines
RelateObjects
DisplayDataOnImage
ApplyThreshold
ApplyThreshold
IdentifyPrimaryObjects
RelateObjects
OverlayOutlines
MeasureObjectIntensity
MeasureObjectIntensity
MeasureObjectIntensity
MeasureObjectSizeShape
Tile
SaveImages
ConvertObjectsToImage
SaveImages
ExportToSpreadsheet
Output
View output settings
Adjust modules:
Start Test Mode
Analyze Images
Module notes
The NamesAndTypes module allows you to assign a meaningful name to each image by which other modules will refer to it.
Module settings (NamesAndTypes #03)
Assign a name to
All images
Select the image type
Color image
Name to assign these images
input_images
Set intensity range from
Image bit-depth
Update
input_images
1 Image1.lsm
2 Image10.lsm
3 Image10.lsm
4 Image10.lsm
5 Image10.lsm
6 Image10.lsm
7 Image10.lsm
8 Image10.lsm
9 Image10.lsm
10 Image10.lsm
11 Image10.lsm
12 Image10.lsm
13 Image10.lsm
14 Image10.lsm
15 Image10.lsm
16 Image10.lsm
17 Image10.lsm
18 Image10.lsm
19 Image10.lsm
20 Image10.lsm
21 Image10.lsm
22 Image10.lsm
23 Image10.lsm
24 Image10.lsm
25 Image10.lsm
26 Image10.lsm
27 Image2.lsm
28 Image3.lsm
29 Image4.lsm
30 Image4.lsm
Found 105 image sets

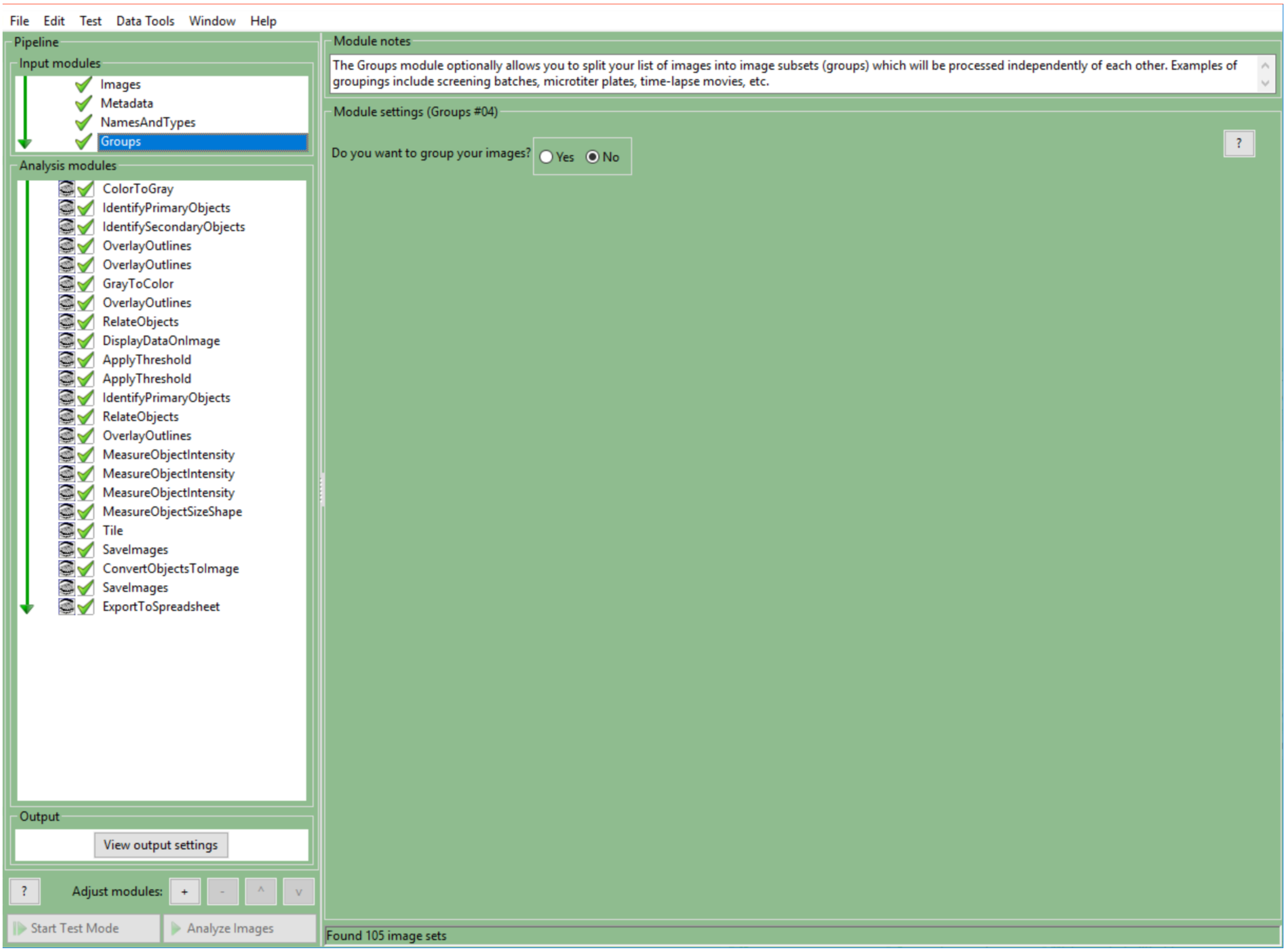
File Edit Test Data Tools Window Help
Pipeline
Input modules
Images
Metadata
NamesAndTypes
Groups
Analysis modules
ColorToGray
IdentifyPrimaryObjects
IdentifySecondaryObjects
OverlayOutlines
OverlayOutlines
GrayToColor
OverlayOutlines
RelateObjects
DisplayDataOnImage
ApplyThreshold
ApplyThreshold
IdentifyPrimaryObjects
RelateObjects
OverlayOutlines
MeasureObjectIntensity
MeasureObjectIntensity
MeasureObjectIntensity
MeasureObjectSizeShape
Tile
SaveImages
ConvertObjectsToImage
SaveImages
ExportToSpreadsheet
Output
View output settings
?
Adjust modules:
Start Test Mode
Analyze Images
Module notes
The Groups module optionally allows you to split your list of images into image subsets (groups) which will be processed independently of each other. Examples of groupings include screening batches, microtiter plates, time-lapse movies, etc.
Module settings (Groups #04)
Do you want to group your images?
Yes
No
Found 105 image sets

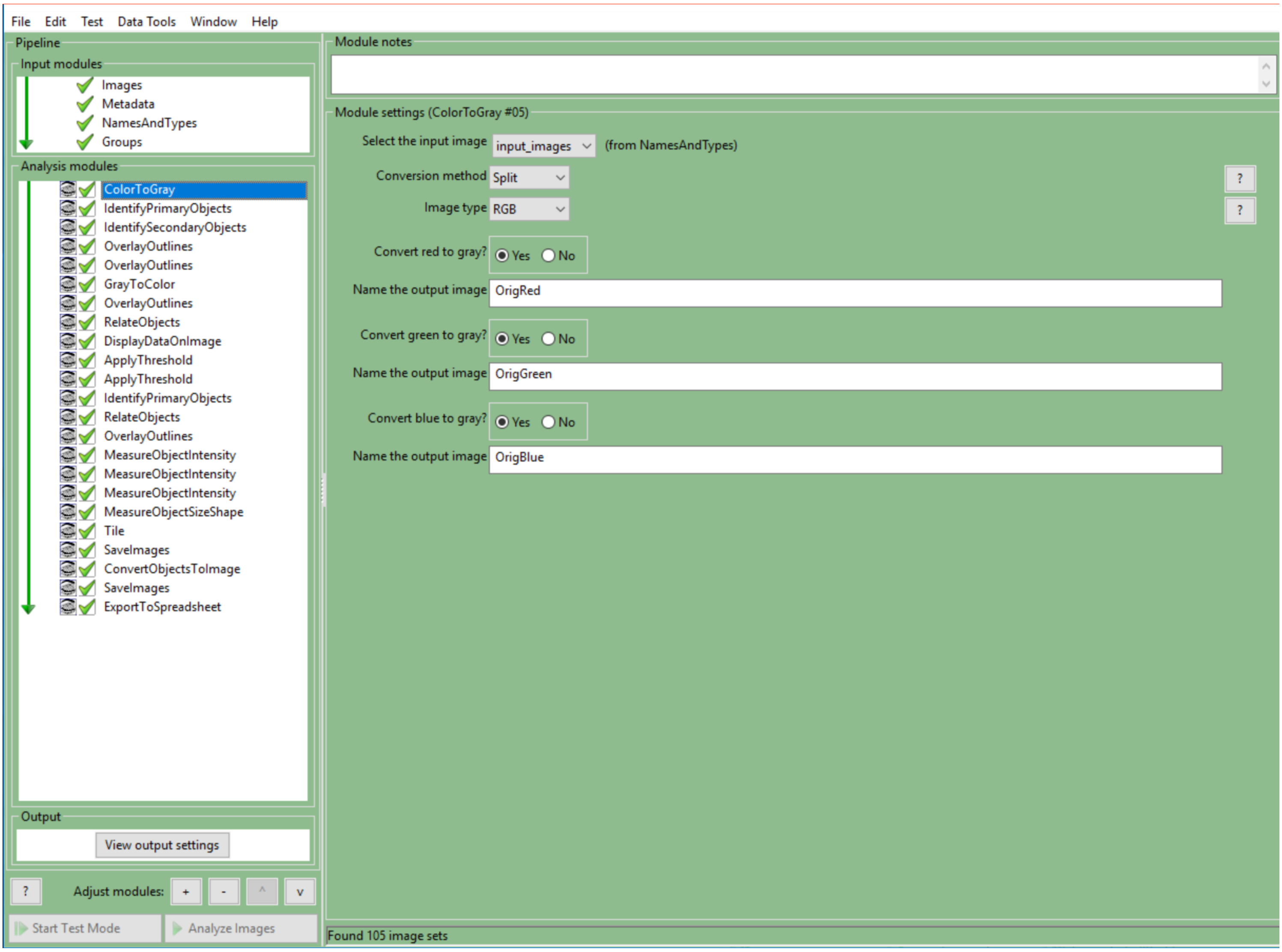
File Edit Test Data Tools Window Help
Pipeline
Input modules
Images
Metadata
NamesAndTypes
Groups
Analysis modules
ColorToGray
IdentifyPrimaryObjects
IdentifySecondaryObjects
OverlayOutlines
OverlayOutlines
GrayToColor
OverlayOutlines
RelateObjects
DisplayDataOnImage
ApplyThreshold
ApplyThreshold
IdentifyPrimaryObjects
RelateObjects
OverlayOutlines
MeasureObjectIntensity
MeasureObjectIntensity
MeasureObjectIntensity
MeasureObjectSizeShape
Tile
SaveImages
ConvertObjectsToImage
SaveImages
ExportToSpreadsheet
Output
View output settings
Adjust modules:
Start Test Mode
Analyze Images
Module notes
Module settings (ColorToGray #05)
Select the input image
input_images
(from NamesAndTypes)
Conversion method
Split
Image type
RGB
Convert red to gray?
Yes
No
Name the output image
OrigRed
Convert green to gray?
Yes
No
Name the output image
OrigGreen
Convert blue to gray?
Yes
No
Name the output image
OrigBlue
Found 105 image sets

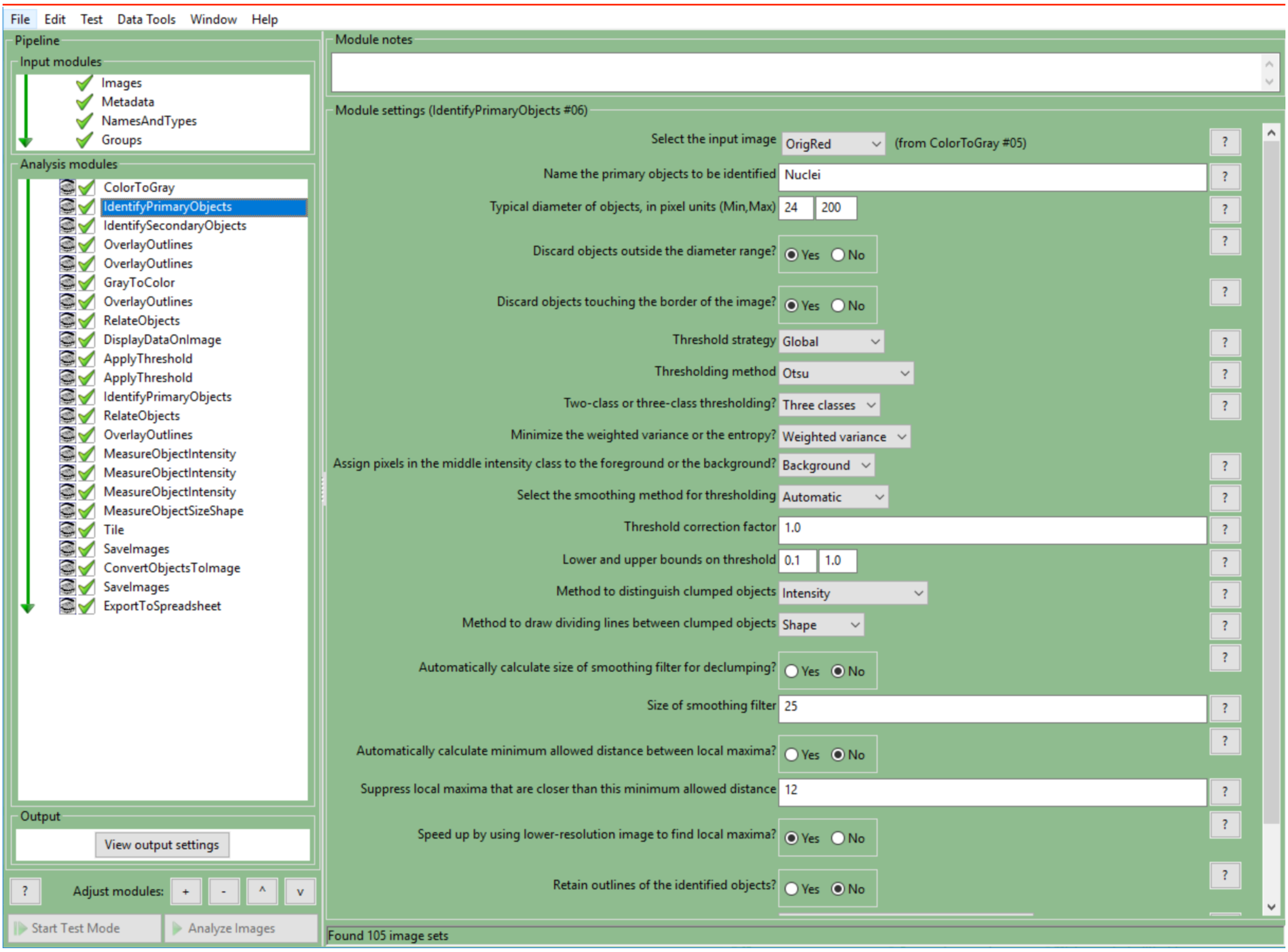
File Edit Test Data Tools Window Help
Pipeline
Input modules
Images
Metadata
NamesAndTypes
Groups
Analysis modules
ColorToGray
IdentifyPrimaryObjects
IdentifySecondaryObjects
OverlayOutlines
OverlayOutlines
GrayToColor
OverlayOutlines
RelateObjects
DisplayDataOnImage
ApplyThreshold
ApplyThreshold
IdentifyPrimaryObjects
RelateObjects
OverlayOutlines
MeasureObjectIntensity
MeasureObjectIntensity
MeasureObjectIntensity
MeasureObjectSizeShape
Tile
SaveImages
ConvertObjectsToImage
SaveImages
ExportToSpreadsheet
Output
View output settings
Adjust modules:
Start Test Mode
Analyze Images
Module notes
Module settings (IdentifyPrimaryObjects #06)
Select the input image OrigRed (from ColorToGray #05)
Name the primary objects to be identified Nuclei
Typical diameter of objects, in pixel units (Min,Max) 24 200
Discard objects outside the diameter range? Yes No
Discard objects touching the border of the image? Yes No
Threshold strategy Global
Thresholding method Otsu
Two-class or three-class thresholding? Three classes
Minimize the weighted variance or the entropy? Weighted variance
Assign pixels in the middle intensity class to the foreground or the background? Background
Select the smoothing method for thresholding Automatic
Threshold correction factor 1.0
Lower and upper bounds on threshold 0.1 1.0
Method to distinguish clumped objects Intensity
Method to draw dividing lines between clumped objects Shape
Automatically calculate size of smoothing filter for declumping? Yes No
Size of smoothing filter 25
Automatically calculate minimum allowed distance between local maxima? Yes No
Suppress local maxima that are closer than this minimum allowed distance 12
Speed up by using lower-resolution image to find local maxima? Yes No
Retain outlines of the identified objects? Yes No
Found 105 image sets

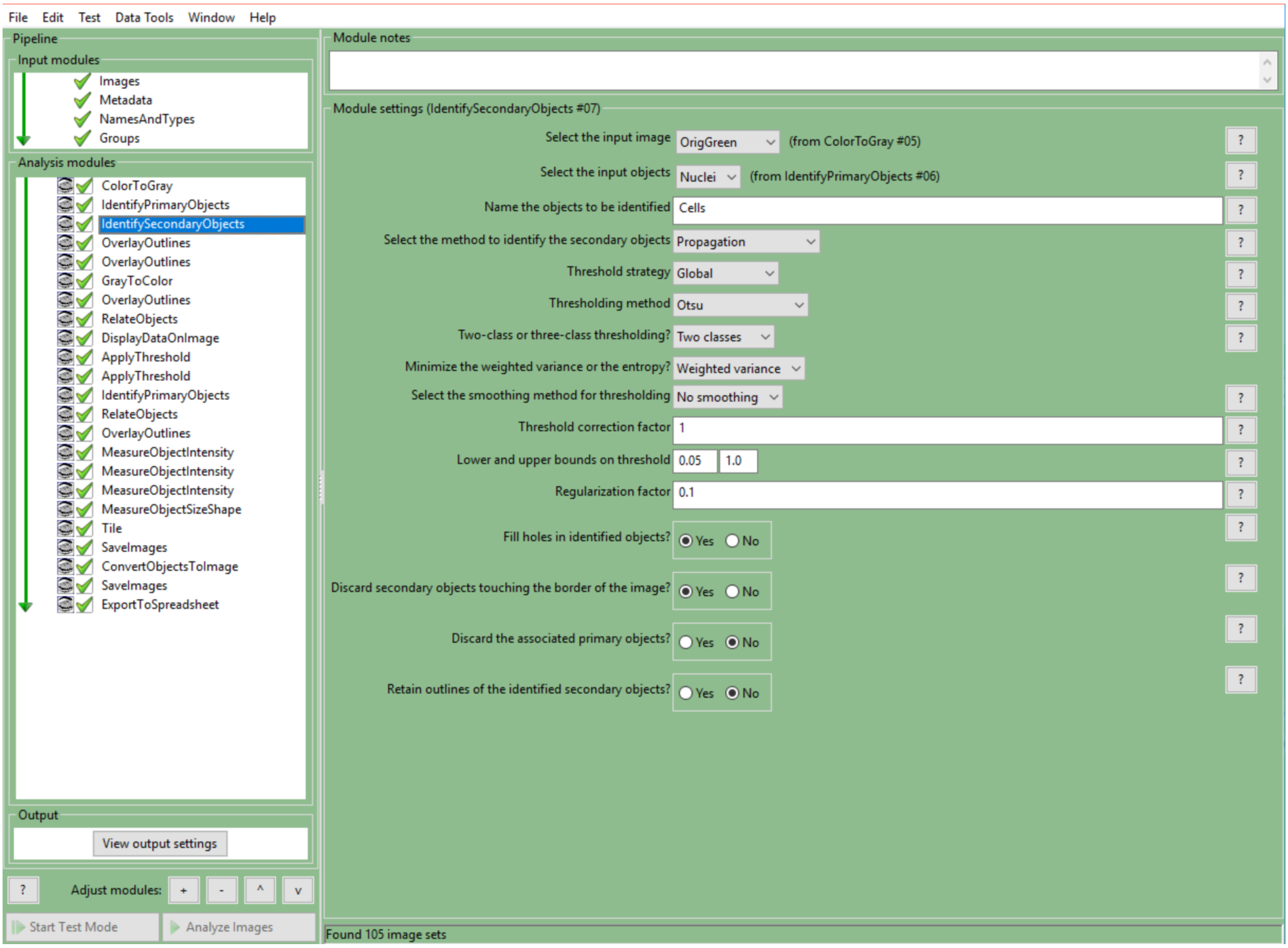
File Edit Test Data Tools Window Help
Pipeline
Input modules
Images
Metadata
NamesAndTypes
Groups
Analysis modules
ColorToGray
IdentifyPrimaryObjects
IdentifySecondaryObjects
OverlayOutlines
OverlayOutlines
GrayToColor
OverlayOutlines
RelateObjects
DisplayDataOnImage
ApplyThreshold
ApplyThreshold
IdentifyPrimaryObjects
RelateObjects
OverlayOutlines
MeasureObjectIntensity
MeasureObjectIntensity
MeasureObjectIntensity
MeasureObjectSizeShape
Tile
SaveImages
ConvertObjectsToImage
SaveImages
ExportToSpreadsheet
Output
View output settings
Adjust modules:
Start Test Mode
Analyze Images
Module notes
Module settings (IdentifySecondaryObjects #07)
Select the input image
OrigGreen
(from ColorToGray #05)
Select the input objects
Nuclei
(from IdentifyPrimaryObjects #06)
Name the objects to be identified
Cells
Select the method to identify the secondary objects
Propagation
Threshold strategy
Global
Thresholding method
Otsu
Two-class or three-class thresholding?
Two classes
Minimize the weighted variance or the entropy?
Weighted variance
Select the smoothing method for thresholding
No smoothing
Threshold correction factor
1
Lower and upper bounds on threshold
0.05
1.0
Regularization factor
0.1
Fill holes in identified objects?
Yes No
Discard secondary objects touching the border of the image?
Yes No
Discard the associated primary objects?
Yes No
Retain outlines of the identified secondary objects?
Yes No
Found 105 image sets

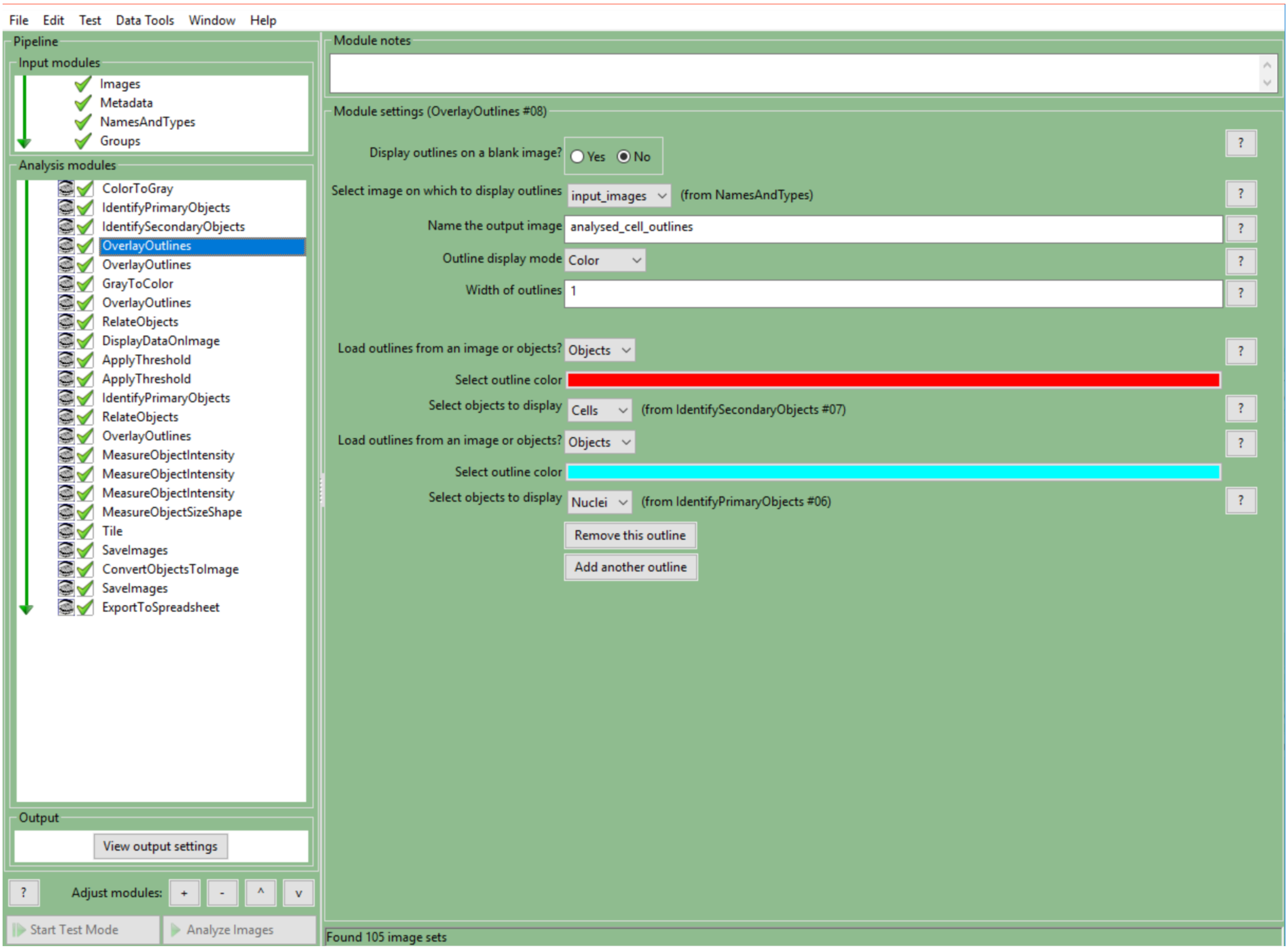
File Edit Test Data Tools Window Help
Pipeline
Input modules
Images
Metadata
NamesAndTypes
Groups
Analysis modules
ColorToGray
IdentifyPrimaryObjects
IdentifySecondaryObjects
OverlayOutlines
OverlayOutlines
GrayToColor
OverlayOutlines
RelateObjects
DisplayDataOnImage
ApplyThreshold
ApplyThreshold
IdentifyPrimaryObjects
RelateObjects
OverlayOutlines
MeasureObjectIntensity
MeasureObjectIntensity
MeasureObjectIntensity
MeasureObjectSizeShape
Tile
SaveImages
ConvertObjectsToImage
SaveImages
ExportToSpreadsheet
Output
View output settings
Adjust modules:
Start Test Mode
Analyze Images
Module notes
Module settings (OverlayOutlines #08)
Display outlines on a blank image? Yes No
Select image on which to display outlines input_images (from NamesAndTypes)
Name the output image analysed_cell_outlines
Outline display mode Color
Width of outlines 1
Load outlines from an image or objects? Objects
Select outline color
Select objects to display Cells (from IdentifySecondaryObjects #07)
Load outlines from an image or objects? Objects
Select outline color
Select objects to display Nuclei (from IdentifyPrimaryObjects #06)
Remove this outline
Add another outline
Found 105 image sets

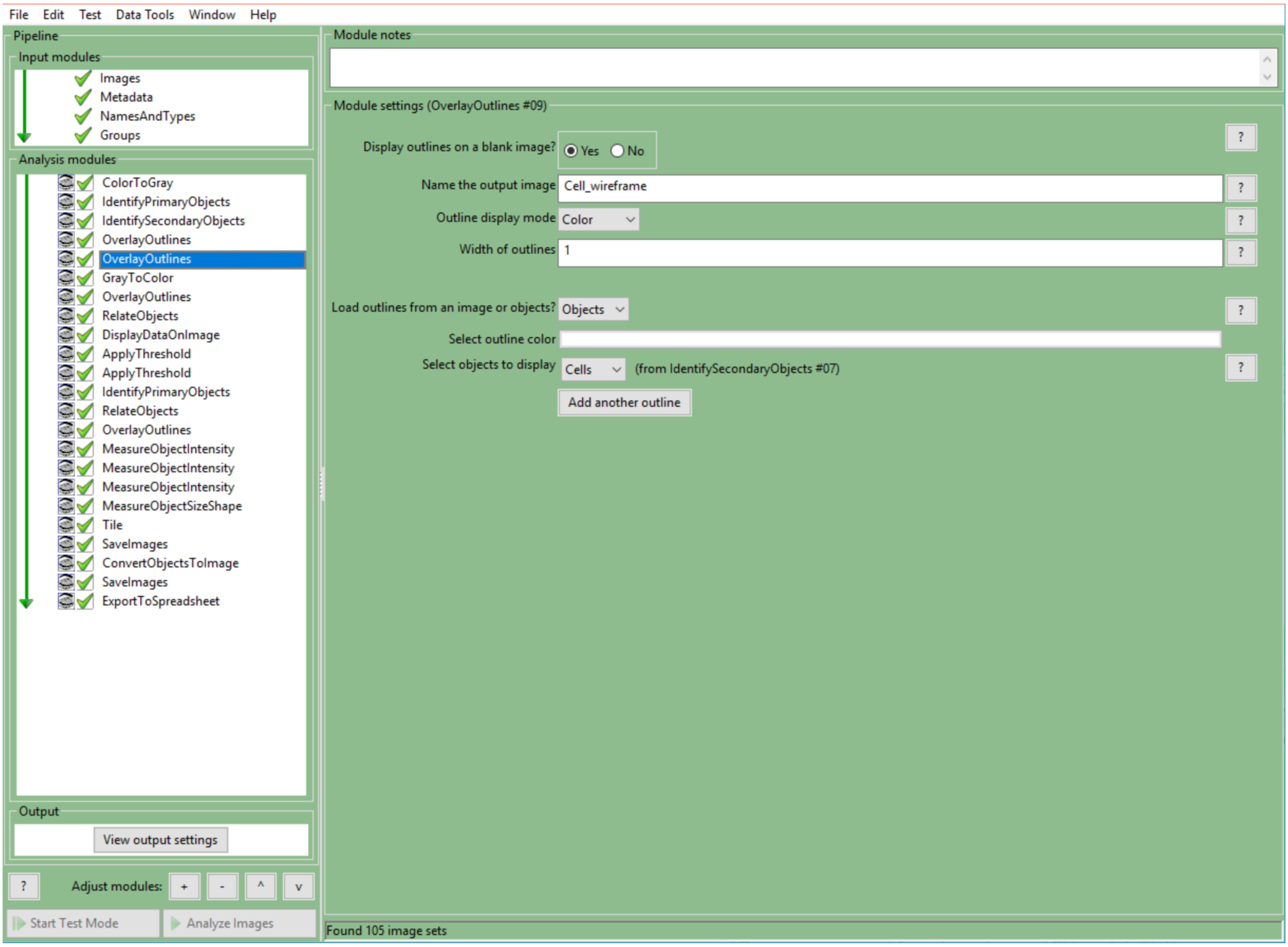
File Edit Test Data Tools Window Help
Pipeline
Input modules
Images
Metadata
NamesAndTypes
Groups
Analysis modules
ColorToGray
IdentifyPrimaryObjects
IdentifySecondaryObjects
OverlayOutlines
OverlayOutlines
GrayToColor
OverlayOutlines
RelateObjects
DisplayDataOnImage
ApplyThreshold
ApplyThreshold
IdentifyPrimaryObjects
RelateObjects
OverlayOutlines
MeasureObjectIntensity
MeasureObjectIntensity
MeasureObjectIntensity
MeasureObjectSizeShape
Tile
SaveImages
ConvertObjectsToImage
SaveImages
ExportToSpreadsheet
Output
View output settings
Adjust modules:
Start Test Mode
Analyze Images
Module notes
Module settings (OverlayOutlines #09)
Display outlines on a blank image?
Yes
No
Name the output image
Cell_wireframe
Outline display mode
Color
Width of outlines
1
Load outlines from an image or objects?
Objects
Select outline color
Select objects to display
Cells
(from IdentifySecondaryObjects #07)
Add another outline
Found 105 image sets

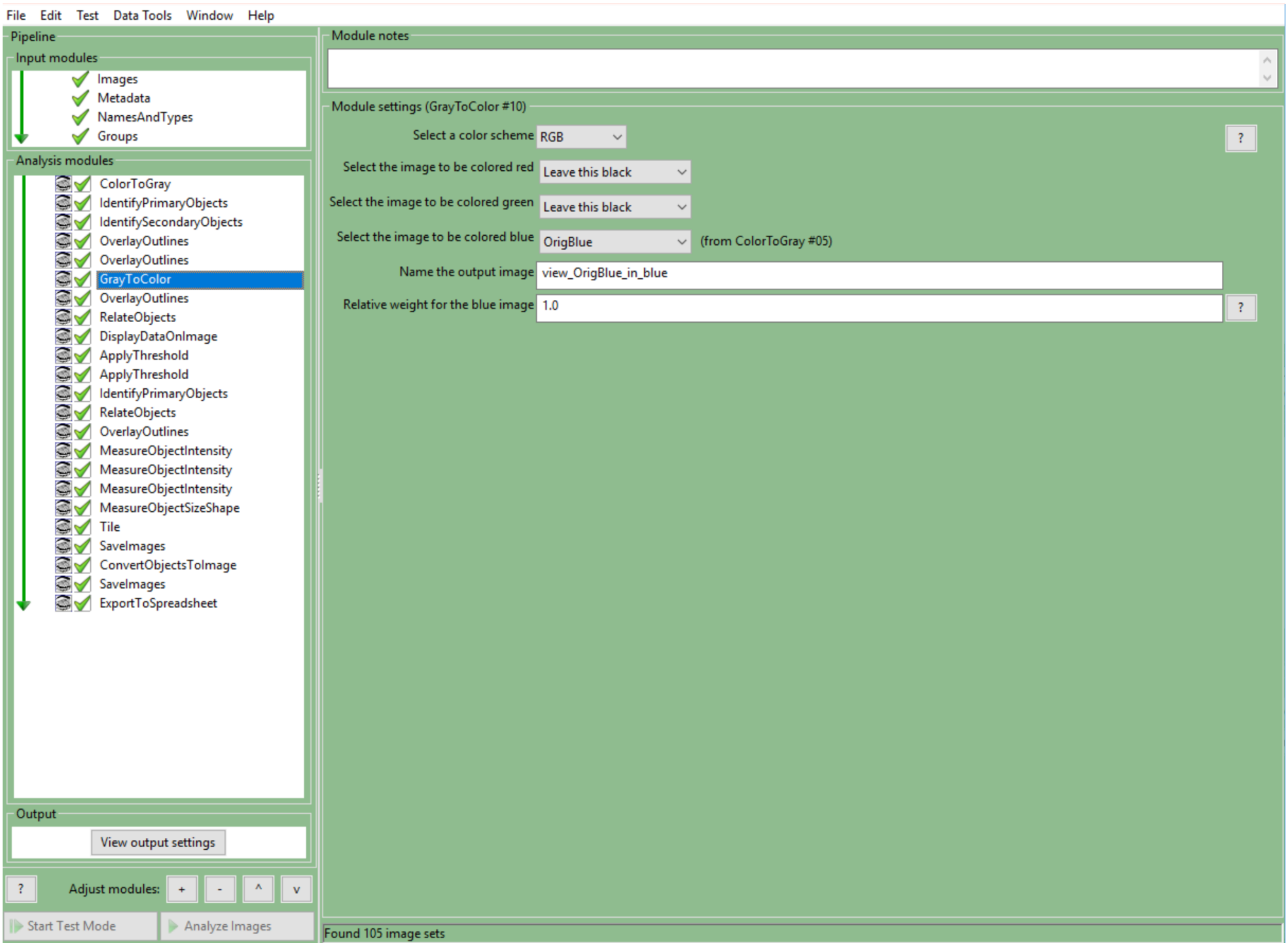
File Edit Test Data Tools Window Help
Pipeline
Input modules
Images
Metadata
NamesAndTypes
Groups
Analysis modules
ColorToGray
IdentifyPrimaryObjects
IdentifySecondaryObjects
OverlayOutlines
OverlayOutlines
GrayToColor
OverlayOutlines
RelateObjects
DisplayDataOnImage
ApplyThreshold
ApplyThreshold
IdentifyPrimaryObjects
RelateObjects
OverlayOutlines
MeasureObjectIntensity
MeasureObjectIntensity
MeasureObjectIntensity
MeasureObjectSizeShape
Tile
SaveImages
ConvertObjectsToImage
SaveImages
ExportToSpreadsheet
Output
View output settings
Adjust modules:
Start Test Mode
Analyze Images
Module notes
Module settings (GrayToColor #10)
Select a color scheme RGB
Select the image to be colored red Leave this black
Select the image to be colored green Leave this black
Select the image to be colored blue OrigBlue (from ColorToGray #05)
Name the output image view_OrigBlue_in_blue
Relative weight for the blue image 1.0
Found 105 image sets

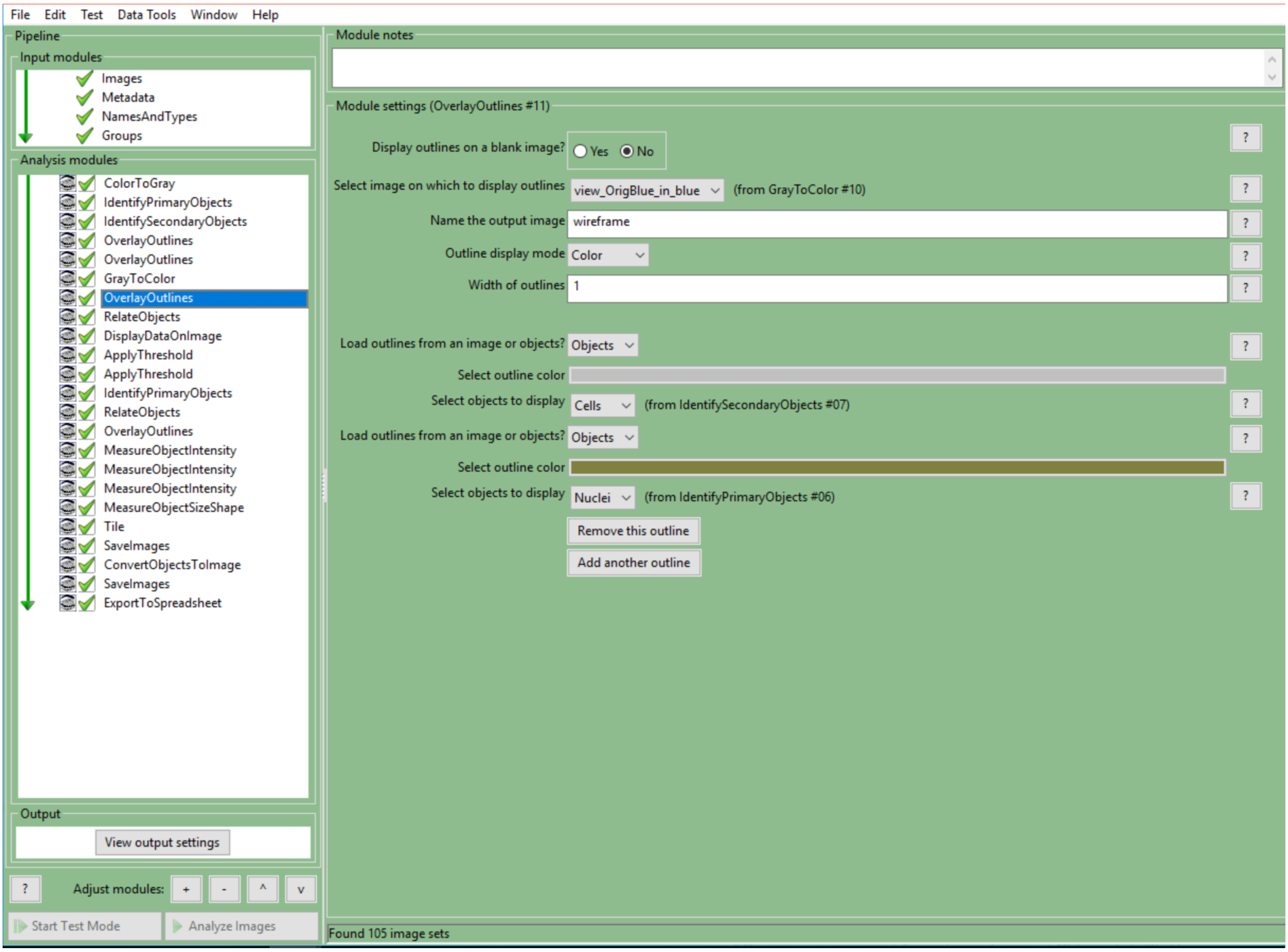
File Edit Test Data Tools Window Help
Pipeline
Input modules
Images
Metadata
NamesAndTypes
Groups
Analysis modules
ColorToGray
IdentifyPrimaryObjects
IdentifySecondaryObjects
OverlayOutlines
OverlayOutlines
GrayToColor
OverlayOutlines
RelateObjects
DisplayDataOnImage
ApplyThreshold
ApplyThreshold
IdentifyPrimaryObjects
RelateObjects
OverlayOutlines
MeasureObjectIntensity
MeasureObjectIntensity
MeasureObjectIntensity
MeasureObjectSizeShape
Tile
SaveImages
ConvertObjectsToImage
SaveImages
ExportToSpreadsheet
Output
View output settings
Adjust modules: + - ^ v
Start Test Mode
Analyze Images
Module notes
Module settings (OverlayOutlines #11)
Display outlines on a blank image? Yes No
Select image on which to display outlines view_OrigBlue_in_blue (from GrayToColor #10)
Name the output image wireframe
Outline display mode Color
Width of outlines 1
Load outlines from an image or objects? Objects
Select outline color
Select objects to display Cells (from IdentifySecondaryObjects #07)
Load outlines from an image or objects? Objects
Select outline color
Select objects to display Nuclei (from IdentifyPrimaryObjects #06)
Remove this outline
Add another outline
Found 105 image sets

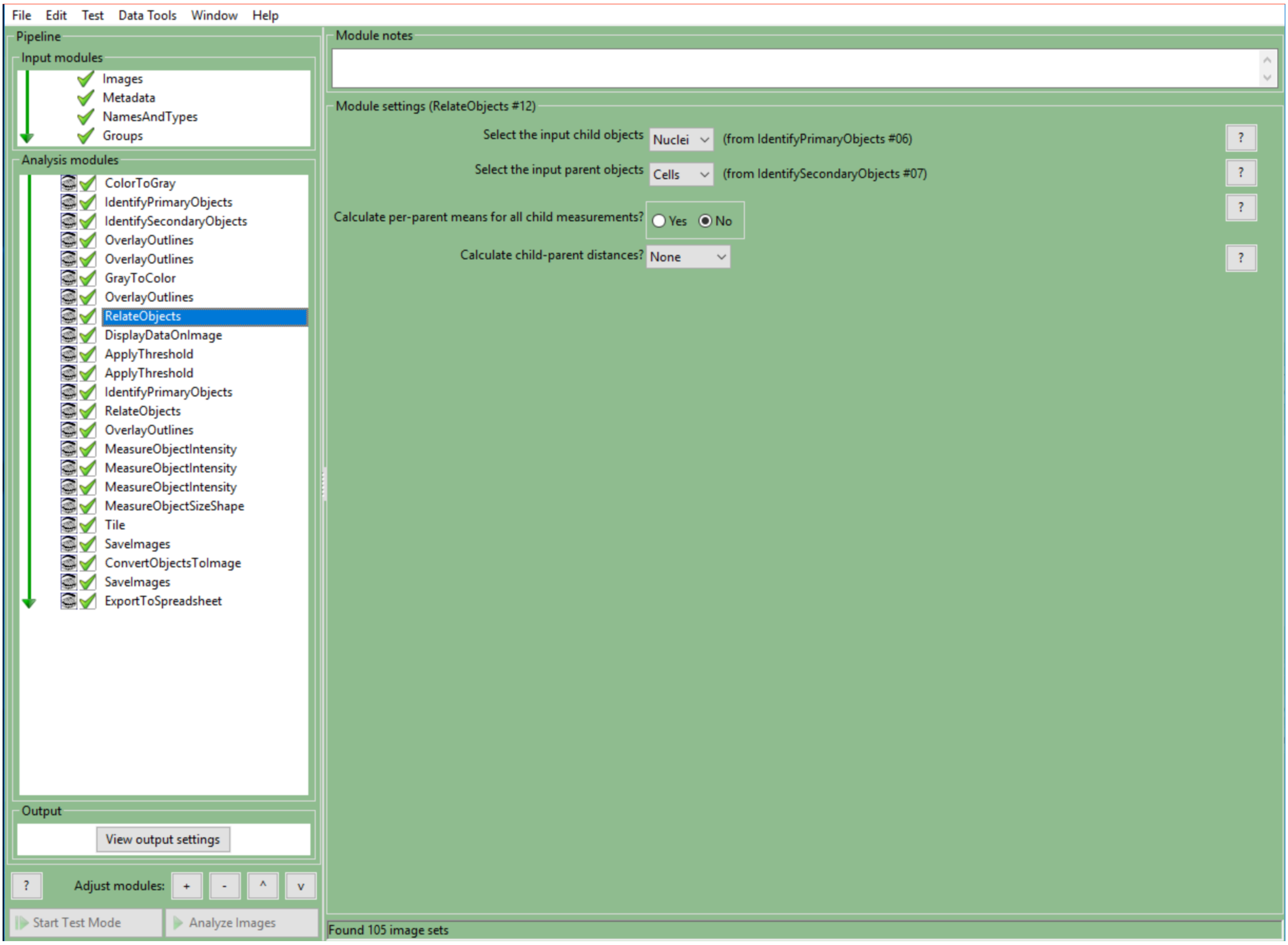
File Edit Test Data Tools Window Help
Pipeline
Input modules
Images
Metadata
NamesAndTypes
Groups
Analysis modules
ColorToGray
IdentifyPrimaryObjects
IdentifySecondaryObjects
OverlayOutlines
OverlayOutlines
GrayToColor
OverlayOutlines
RelateObjects
DisplayDataOnImage
ApplyThreshold
ApplyThreshold
IdentifyPrimaryObjects
RelateObjects
OverlayOutlines
MeasureObjectIntensity
MeasureObjectIntensity
MeasureObjectIntensity
MeasureObjectSizeShape
Tile
SaveImages
ConvertObjectsToImage
SaveImages
ExportToSpreadsheet
Output
View output settings
Adjust modules:
Start Test Mode
Analyze Images
Module notes
Module settings (RelateObjects #12)
Select the input child objects
Nuclei
(from IdentifyPrimaryObjects #06)
Select the input parent objects
Cells
(from IdentifySecondaryObjects #07)
Calculate per-parent means for all child measurements?
Yes
No
Calculate child-parent distances?
None
Found 105 image sets

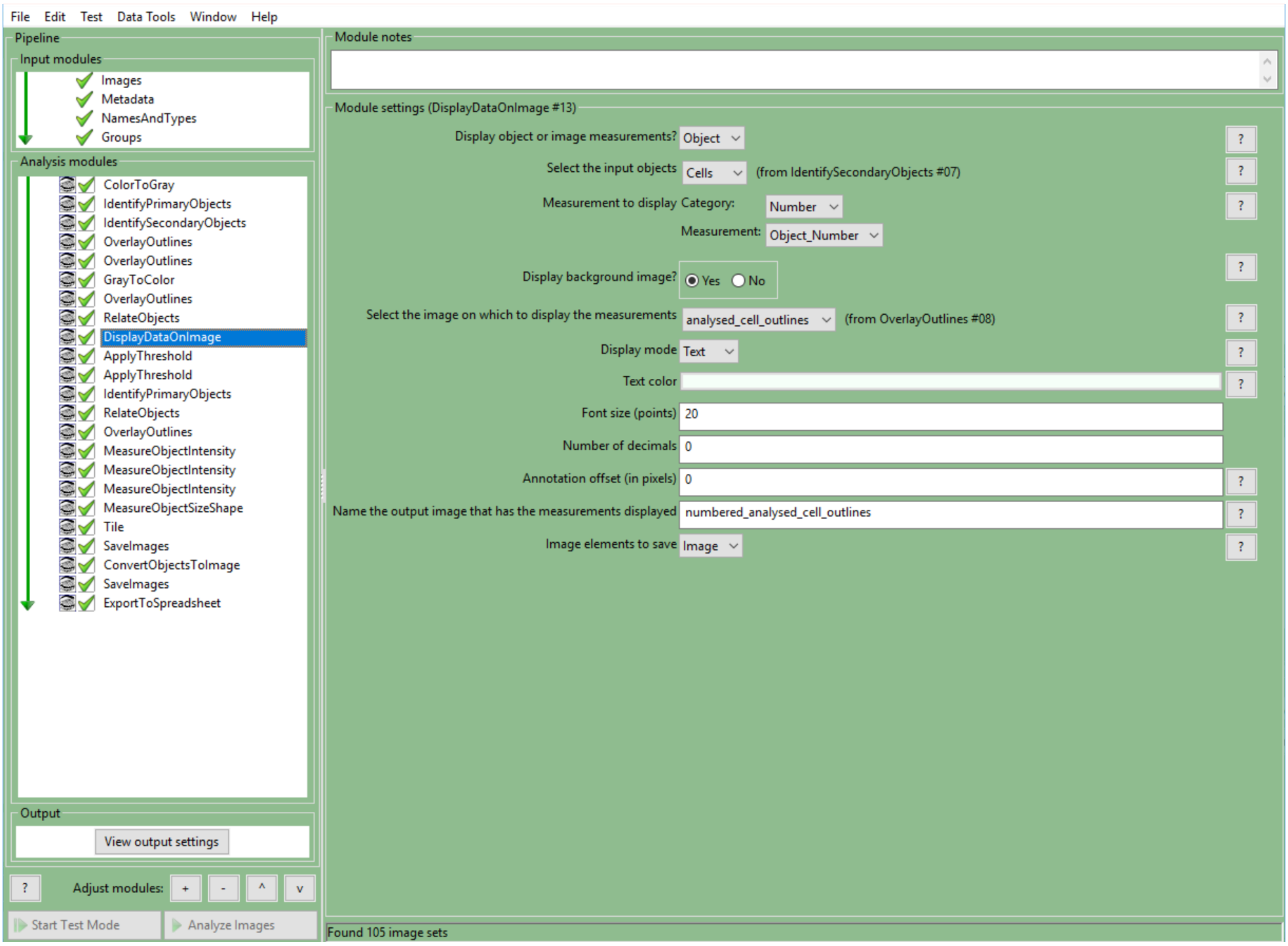
File Edit Test Data Tools Window Help
Pipeline
Input modules
Images
Metadata
NamesAndTypes
Groups
Analysis modules
ColorToGray
IdentifyPrimaryObjects
IdentifySecondaryObjects
OverlayOutlines
OverlayOutlines
GrayToColor
OverlayOutlines
RelateObjects
DisplayDataOnImage
ApplyThreshold
ApplyThreshold
IdentifyPrimaryObjects
RelateObjects
OverlayOutlines
MeasureObjectIntensity
MeasureObjectIntensity
MeasureObjectIntensity
MeasureObjectSizeShape
Tile
SaveImages
ConvertObjectsToImage
SaveImages
ExportToSpreadsheet
Output
View output settings
Adjust modules:
Start Test Mode
Analyze Images
Module notes
Module settings (DisplayDataOnImage #13)
Display object or image measurements? Object
Select the input objects Cells (from IdentifySecondaryObjects #07)
Measurement to display Category: Number
Measurement: Object_Number
Display background image? Yes No
Select the image on which to display the measurements analysed_cell_outlines (from OverlayOutlines #08)
Display mode Text
Text color
Font size (points) 20
Number of decimals 0
Annotation offset (in pixels) 0
Name the output image that has the measurements displayed numbered_analysed_cell_outlines
Image elements to save Image
Found 105 image sets

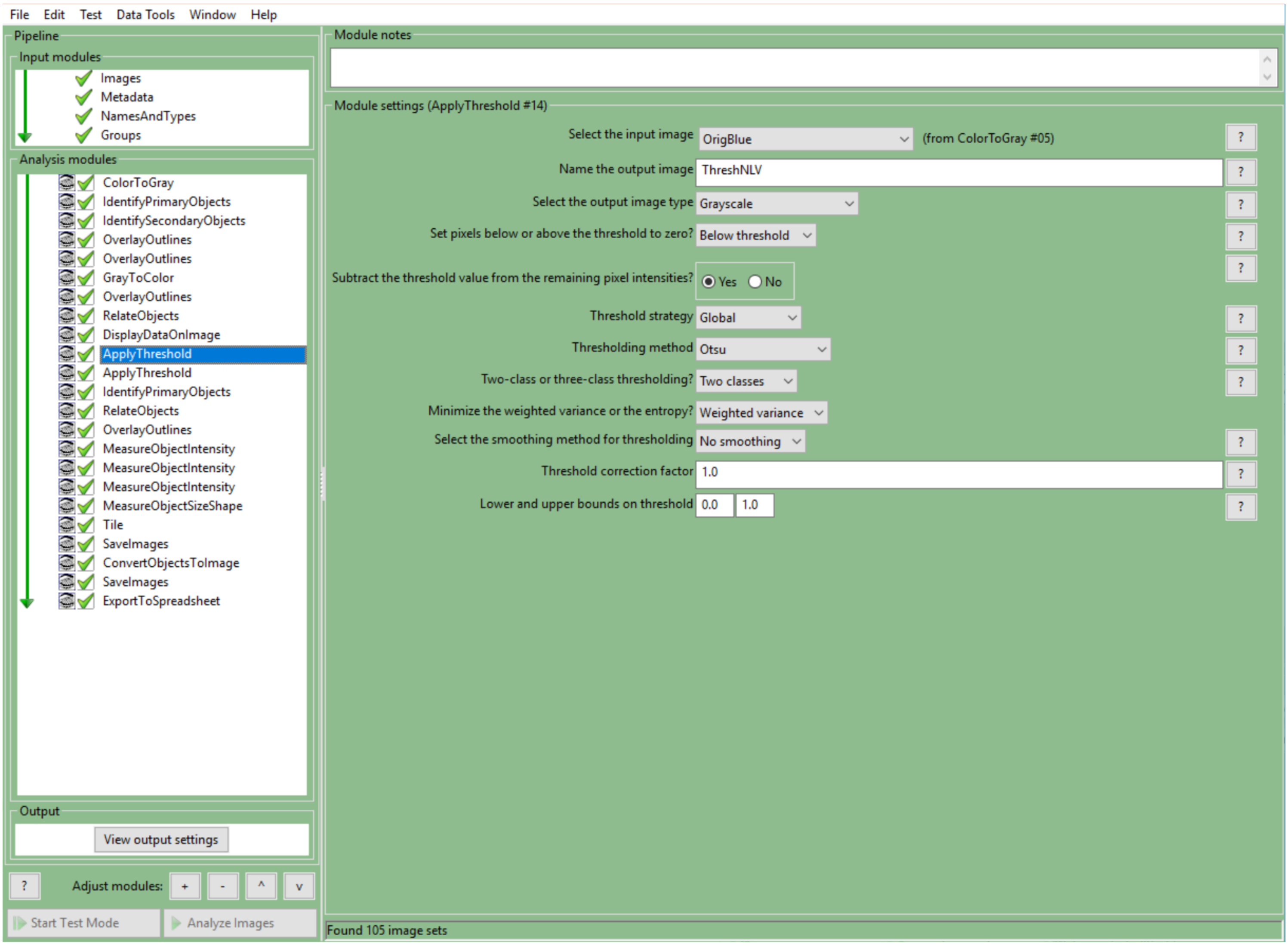

File Edit Test Data Tools Window Help
Pipeline
Input modules
Images
Metadata
NamesAndTypes
Groups
Analysis modules
ColorToGray
IdentifyPrimaryObjects
IdentifySecondaryObjects
OverlayOutlines
OverlayOutlines
GrayToColor
OverlayOutlines
RelateObjects
DisplayDataOnImage
ApplyThreshold
ApplyThreshold
IdentifyPrimaryObjects
RelateObjects
OverlayOutlines
MeasureObjectIntensity
MeasureObjectIntensity
MeasureObjectIntensity
MeasureObjectSizeShape
Tile
SaveImages
ConvertObjectsToImage
SaveImages
ExportToSpreadsheet
Output
View output settings
Adjust modules:
Start Test Mode
Analyze Images
Module notes
Module settings (ApplyThreshold #14)
Select the input image
OrigBlue
(from ColorToGray #05)
Name the output image
ThreshNLV
Select the output image type
Grayscale
Set pixels below or above the threshold to zero?
Below threshold
Subtract the threshold value from the remaining pixel intensities?
Yes No
Threshold strategy
Global
Thresholding method
Otsu
Two-class or three-class thresholding?
Two classes
Minimize the weighted variance or the entropy?
Weighted variance
Select the smoothing method for thresholding
No smoothing
Threshold correction factor
1.0
Lower and upper bounds on threshold
0.0
1.0
Found 105 image sets

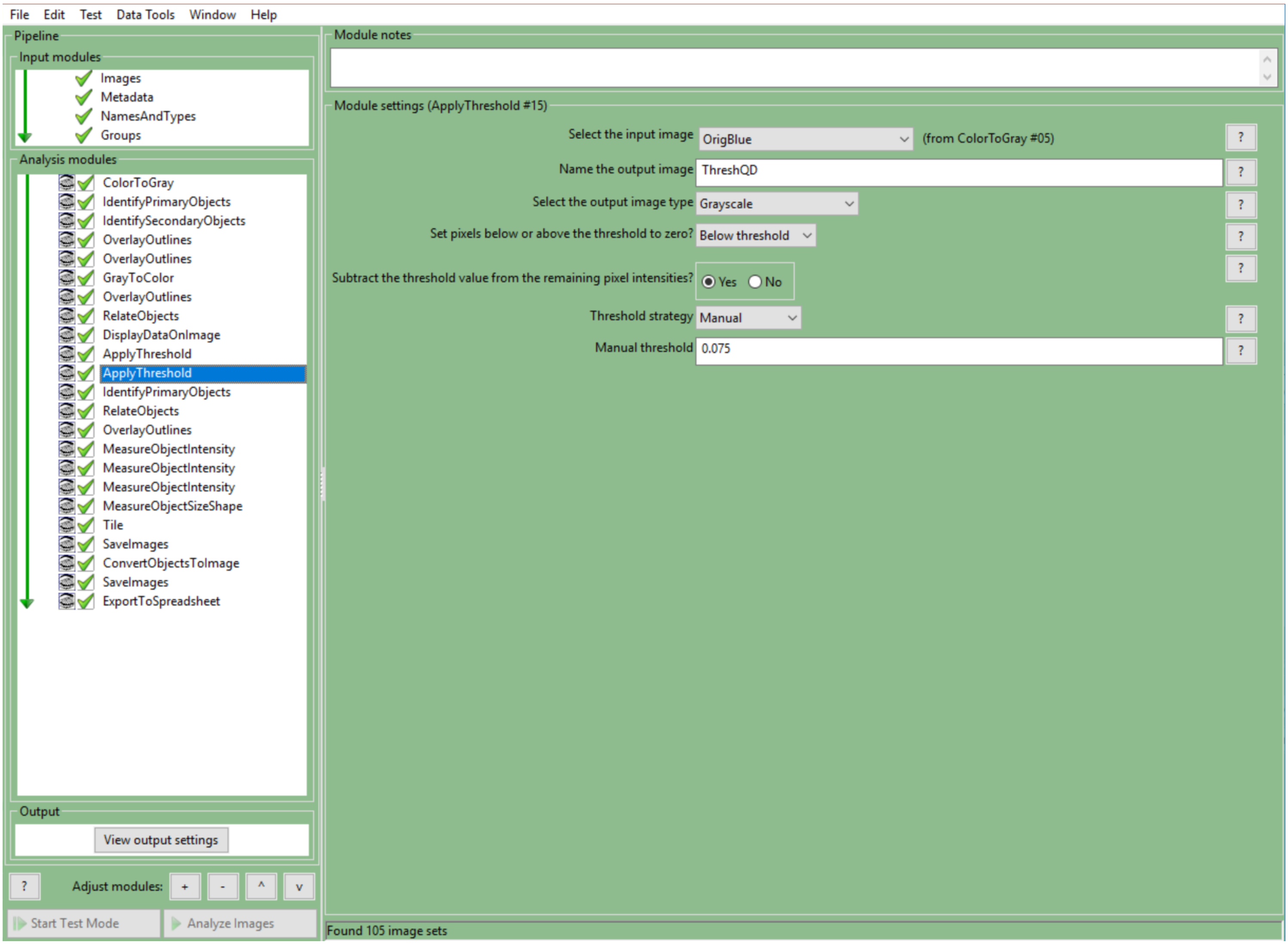
File Edit Test Data Tools Window Help
Pipeline
Input modules
Images
Metadata
NamesAndTypes
Groups
Analysis modules
ColorToGray
IdentifyPrimaryObjects
IdentifySecondaryObjects
OverlayOutlines
OverlayOutlines
GrayToColor
OverlayOutlines
RelateObjects
DisplayDataOnImage
ApplyThreshold
ApplyThreshold
IdentifyPrimaryObjects
RelateObjects
OverlayOutlines
MeasureObjectIntensity
MeasureObjectIntensity
MeasureObjectIntensity
MeasureObjectSizeShape
Tile
SaveImages
ConvertObjectsToImage
SaveImages
ExportToSpreadsheet
Output
View output settings
Adjust modules:
Start Test Mode
Analyze Images
Module notes
Module settings (ApplyThreshold #15)
Select the input image
OrigBlue
(from ColorToGray #05)
Name the output image
ThreshQD
Select the output image type
Grayscale
Set pixels below or above the threshold to zero?
Below threshold
Subtract the threshold value from the remaining pixel intensities?
Yes
No
Threshold strategy
Manual
Manual threshold
0.075
Found 105 image sets

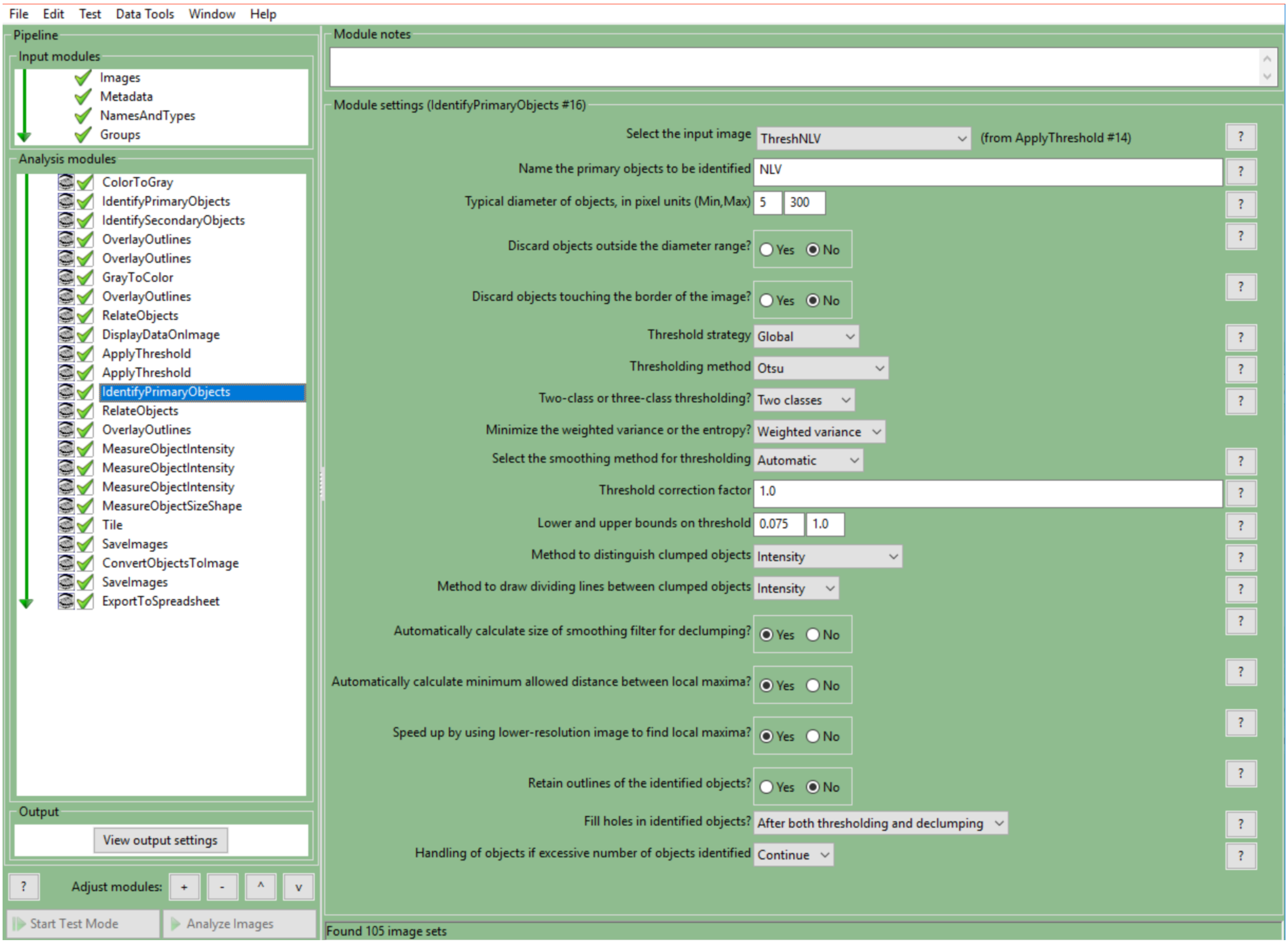

File Edit Test Data Tools Window Help
Pipeline
Input modules
Images
Metadata
NamesAndTypes
Groups
Analysis modules
ColorToGray
IdentifyPrimaryObjects
IdentifySecondaryObjects
OverlayOutlines
OverlayOutlines
GrayToColor
OverlayOutlines
RelateObjects
DisplayDataOnImage
ApplyThreshold
ApplyThreshold
IdentifyPrimaryObjects
RelateObjects
OverlayOutlines
MeasureObjectIntensity
MeasureObjectIntensity
MeasureObjectIntensity
MeasureObjectSizeShape
Tile
SaveImages
ConvertObjectsToImage
SaveImages
ExportToSpreadsheet
Output
View output settings
Adjust modules:
Start Test Mode
Analyze Images
Module notes
Module settings (IdentifyPrimaryObjects #16)
Select the input image ThreshNLV (from ApplyThreshold #14)
Name the primary objects to be identified NLV
Typical diameter of objects, in pixel units (Min,Max) 5 300
Discard objects outside the diameter range? Yes No
Discard objects touching the border of the image? Yes No
Threshold strategy Global
Thresholding method Otsu
Two-class or three-class thresholding? Two classes
Minimize the weighted variance or the entropy? Weighted variance
Select the smoothing method for thresholding Automatic
Threshold correction factor 1.0
Lower and upper bounds on threshold 0.075 1.0
Method to distinguish clumped objects Intensity
Method to draw dividing lines between clumped objects Intensity
Automatically calculate size of smoothing filter for declumping? Yes No
Automatically calculate minimum allowed distance between local maxima? Yes No
Speed up by using lower-resolution image to find local maxima? Yes No
Retain outlines of the identified objects? Yes No
Fill holes in identified objects? After both thresholding and declumping
Handling of objects if excessive number of objects identified Continue
Found 105 image sets

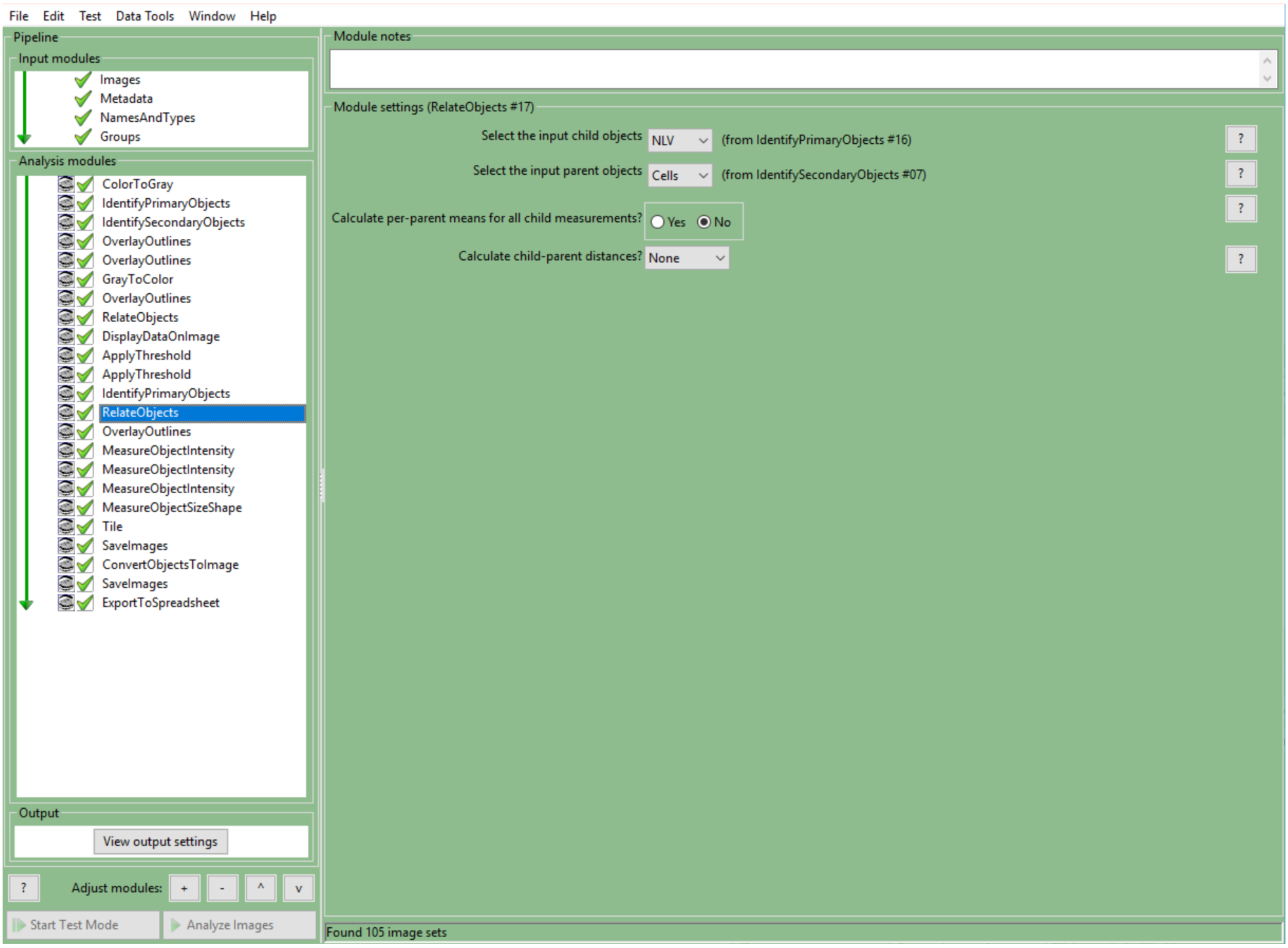
File Edit Test Data Tools Window Help
Pipeline
Input modules
Images
Metadata
NamesAndTypes
Groups
Analysis modules
ColorToGray
IdentifyPrimaryObjects
IdentifySecondaryObjects
OverlayOutlines
OverlayOutlines
GrayToColor
OverlayOutlines
RelateObjects
DisplayDataOnImage
ApplyThreshold
ApplyThreshold
IdentifyPrimaryObjects
RelateObjects
OverlayOutlines
MeasureObjectIntensity
MeasureObjectIntensity
MeasureObjectIntensity
MeasureObjectSizeShape
Tile
SaveImages
ConvertObjectsToImage
SaveImages
ExportToSpreadsheet
Output
View output settings
Adjust modules:
Start Test Mode
Analyze Images
Module notes
Module settings (RelateObjects #17)
Select the input child objects
NLV
(from IdentifyPrimaryObjects #16)
Select the input parent objects
Cells
(from IdentifySecondaryObjects #07)
Calculate per-parent means for all child measurements?
Yes
No
Calculate child-parent distances?
None
Found 105 image sets

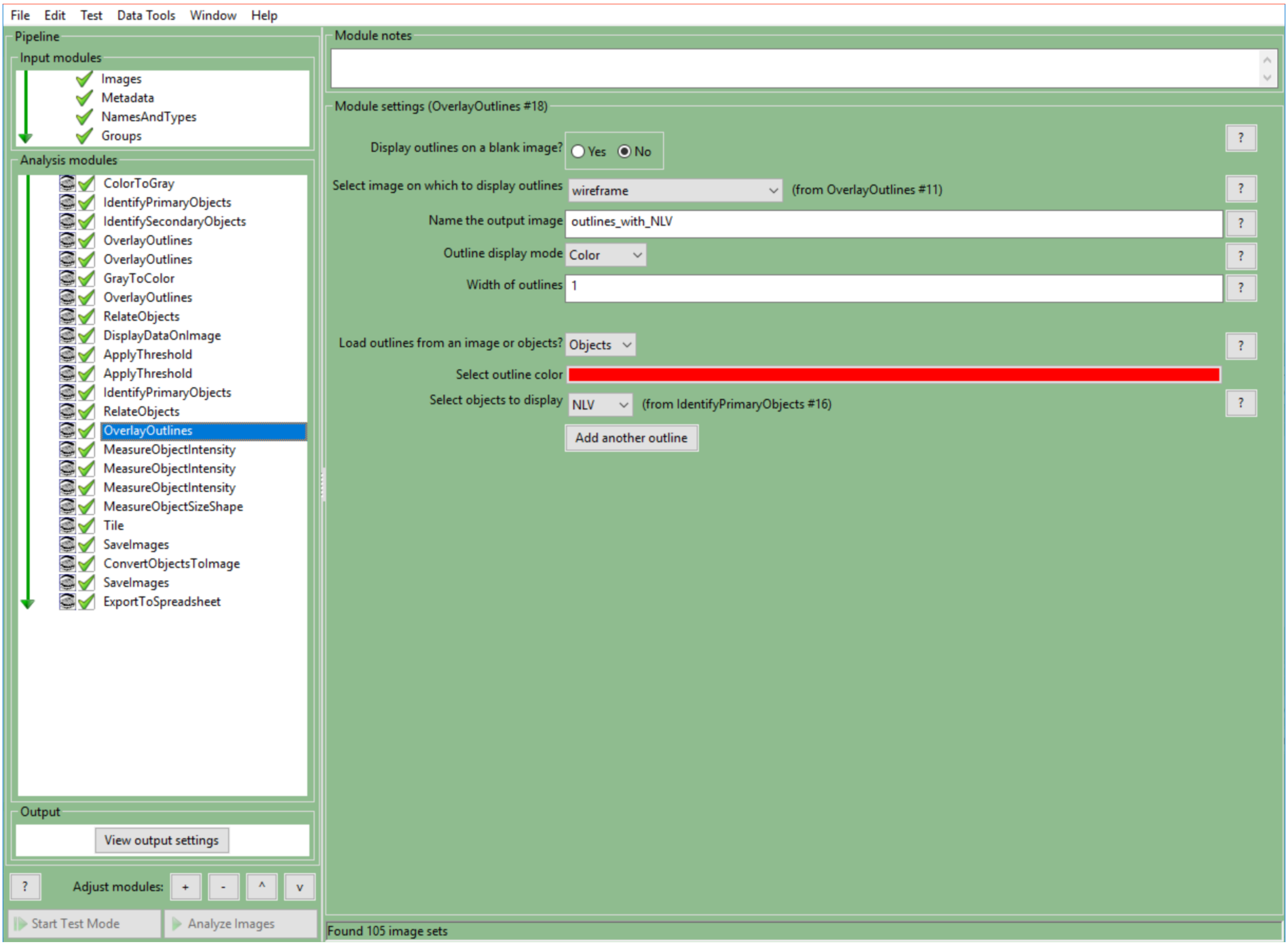
File Edit Test Data Tools Window Help
Pipeline
Input modules
Images
Metadata
NamesAndTypes
Groups
Analysis modules
ColorToGray
IdentifyPrimaryObjects
IdentifySecondaryObjects
OverlayOutlines
OverlayOutlines
GrayToColor
OverlayOutlines
RelateObjects
DisplayDataOnImage
ApplyThreshold
ApplyThreshold
IdentifyPrimaryObjects
RelateObjects
OverlayOutlines
MeasureObjectIntensity
MeasureObjectIntensity
MeasureObjectIntensity
MeasureObjectSizeShape
Tile
SaveImages
ConvertObjectsToImage
SaveImages
ExportToSpreadsheet
Output
View output settings
Adjust modules:
Start Test Mode
Analyze Images
Module notes
Module settings (OverlayOutlines #18)
Display outlines on a blank image? Yes No
Select image on which to display outlines wireframe (from OverlayOutlines #11)
Name the output image outlines_with_NLV
Outline display mode Color
Width of outlines 1
Load outlines from an image or objects? Objects
Select outline color
Select objects to display NLV (from IdentifyPrimaryObjects #16)
Add another outline
Found 105 image sets

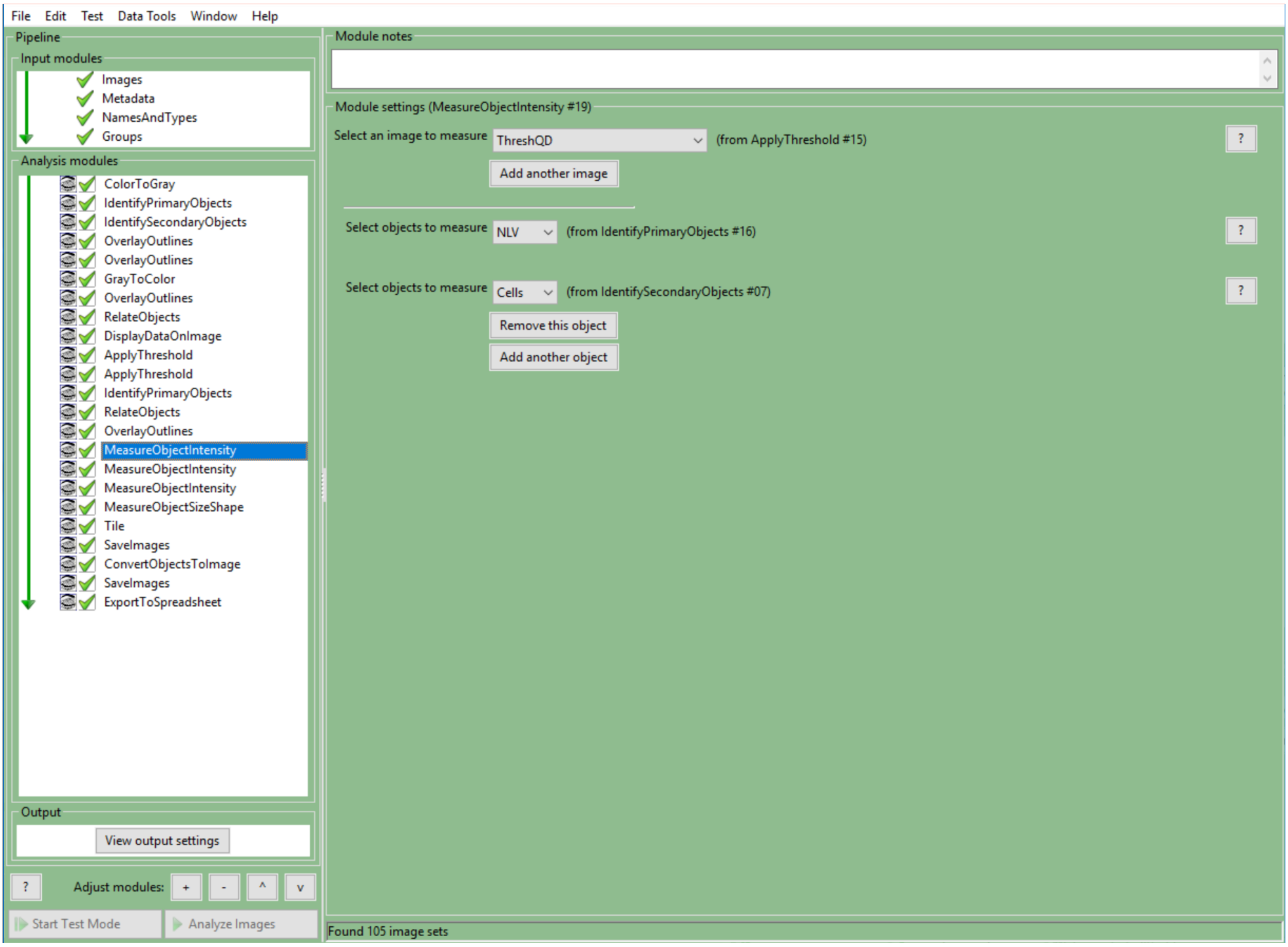
File Edit Test Data Tools Window Help
Pipeline
Input modules
Images
Metadata
NamesAndTypes
Groups
Analysis modules
ColorToGray
IdentifyPrimaryObjects
IdentifySecondaryObjects
OverlayOutlines
OverlayOutlines
GrayToColor
OverlayOutlines
RelateObjects
DisplayDataOnImage
ApplyThreshold
ApplyThreshold
IdentifyPrimaryObjects
RelateObjects
OverlayOutlines
MeasureObjectIntensity
MeasureObjectIntensity
MeasureObjectIntensity
MeasureObjectSizeShape
Tile
SaveImages
ConvertObjectsToImage
SaveImages
ExportToSpreadsheet
Output
View output settings
Adjust modules:
Start Test Mode
Analyze Images
Module notes
Module settings (MeasureObjectIntensity #19)
Select an image to measure
ThreshQD
(from ApplyThreshold #15)
Add another image
Select objects to measure
NLV
(from IdentifyPrimaryObjects #16)
Select objects to measure
Cells
(from IdentifySecondaryObjects #07)
Remove this object
Add another object
Found 105 image sets

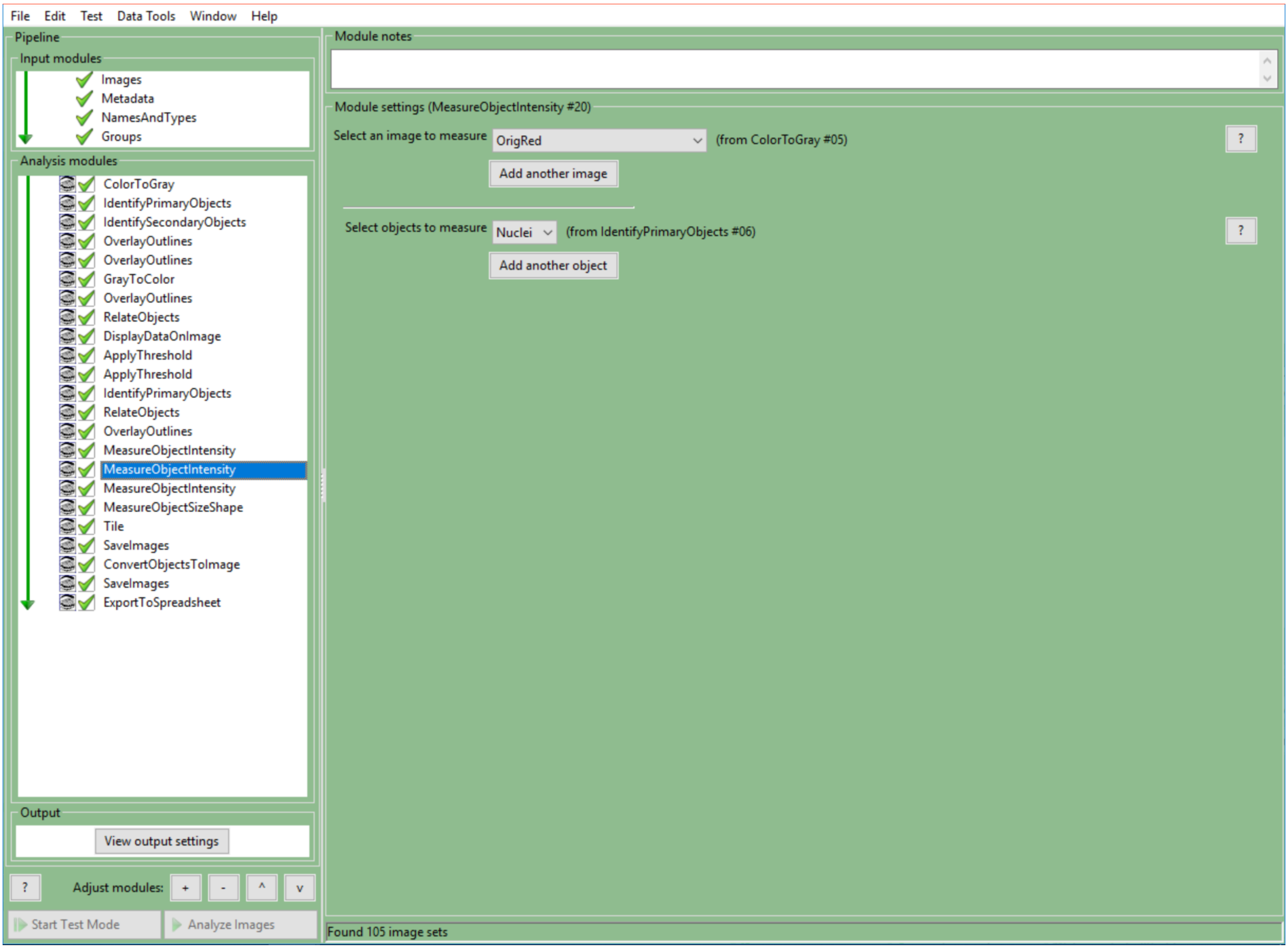
File Edit Test Data Tools Window Help
Pipeline
Input modules
Images
Metadata
NamesAndTypes
Groups
Analysis modules
ColorToGray
IdentifyPrimaryObjects
IdentifySecondaryObjects
OverlayOutlines
OverlayOutlines
GrayToColor
OverlayOutlines
RelateObjects
DisplayDataOnImage
ApplyThreshold
ApplyThreshold
IdentifyPrimaryObjects
RelateObjects
OverlayOutlines
MeasureObjectIntensity
MeasureObjectIntensity
MeasureObjectIntensity
MeasureObjectSizeShape
Tile
SaveImages
ConvertObjectsToImage
SaveImages
ExportToSpreadsheet
Output
View output settings
Adjust modules:
Start Test Mode
Analyze Images
Module notes
Module settings (MeasureObjectIntensity #20)
Select an image to measure
OrigRed
(from ColorToGray #05)
Add another image
Select objects to measure
Nuclei
(from IdentifyPrimaryObjects #06)
Add another object
Found 105 image sets

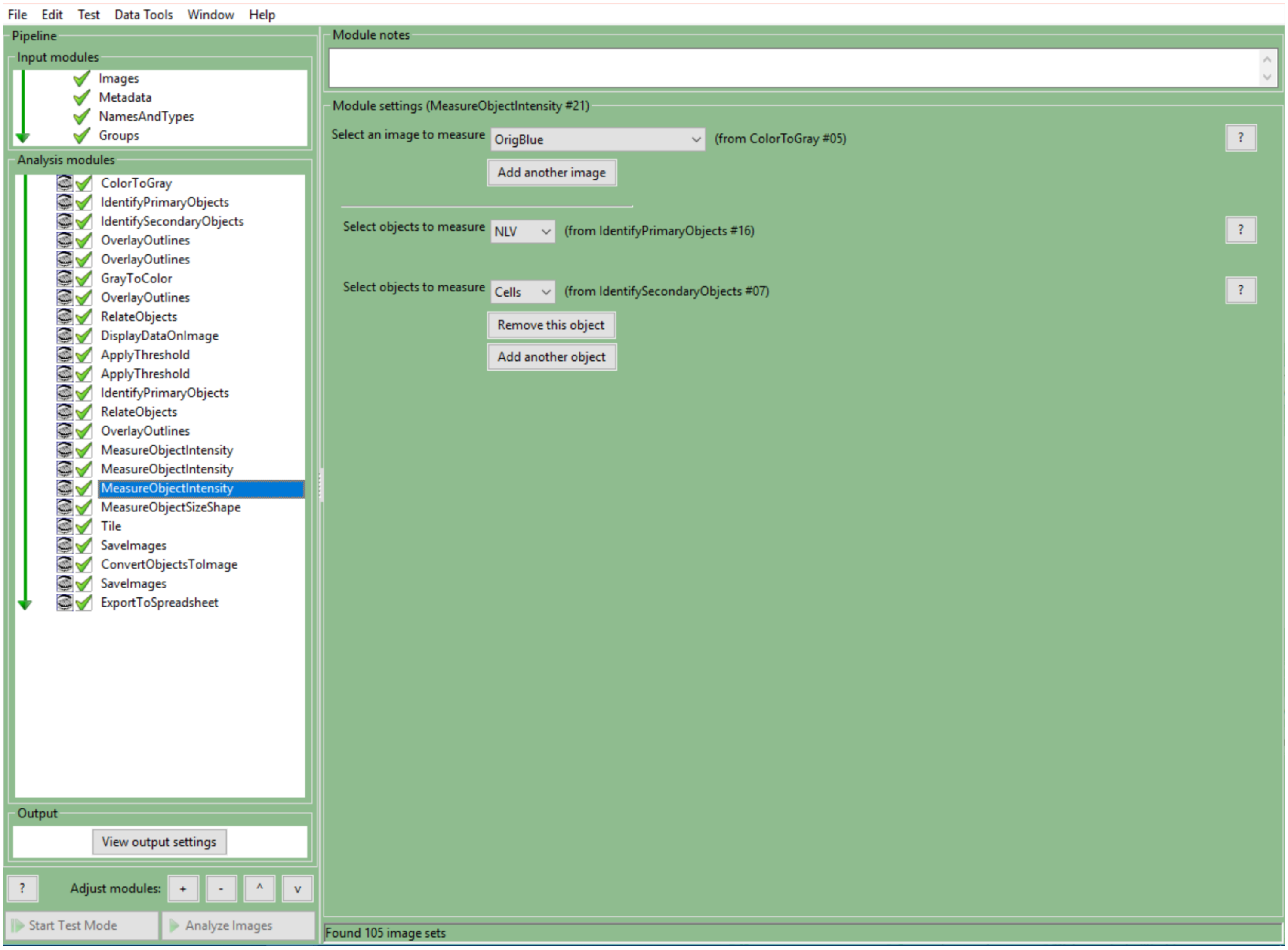
File Edit Test Data Tools Window Help
Pipeline
Input modules
Images
Metadata
NamesAndTypes
Groups
Analysis modules
ColorToGray
IdentifyPrimaryObjects
IdentifySecondaryObjects
OverlayOutlines
OverlayOutlines
GrayToColor
OverlayOutlines
RelateObjects
DisplayDataOnImage
ApplyThreshold
ApplyThreshold
IdentifyPrimaryObjects
RelateObjects
OverlayOutlines
MeasureObjectIntensity
MeasureObjectIntensity
MeasureObjectIntensity
MeasureObjectSizeShape
Tile
SaveImages
ConvertObjectsToImage
SaveImages
ExportToSpreadsheet
Output
View output settings
Adjust modules:
Start Test Mode
Analyze Images
Module notes
Module settings (MeasureObjectIntensity #21)
Select an image to measure
OrigBlue
(from ColorToGray #05)
Add another image
Select objects to measure
NLV
(from IdentifyPrimaryObjects #16)
Select objects to measure
Cells
(from IdentifySecondaryObjects #07)
Remove this object
Add another object
Found 105 image sets

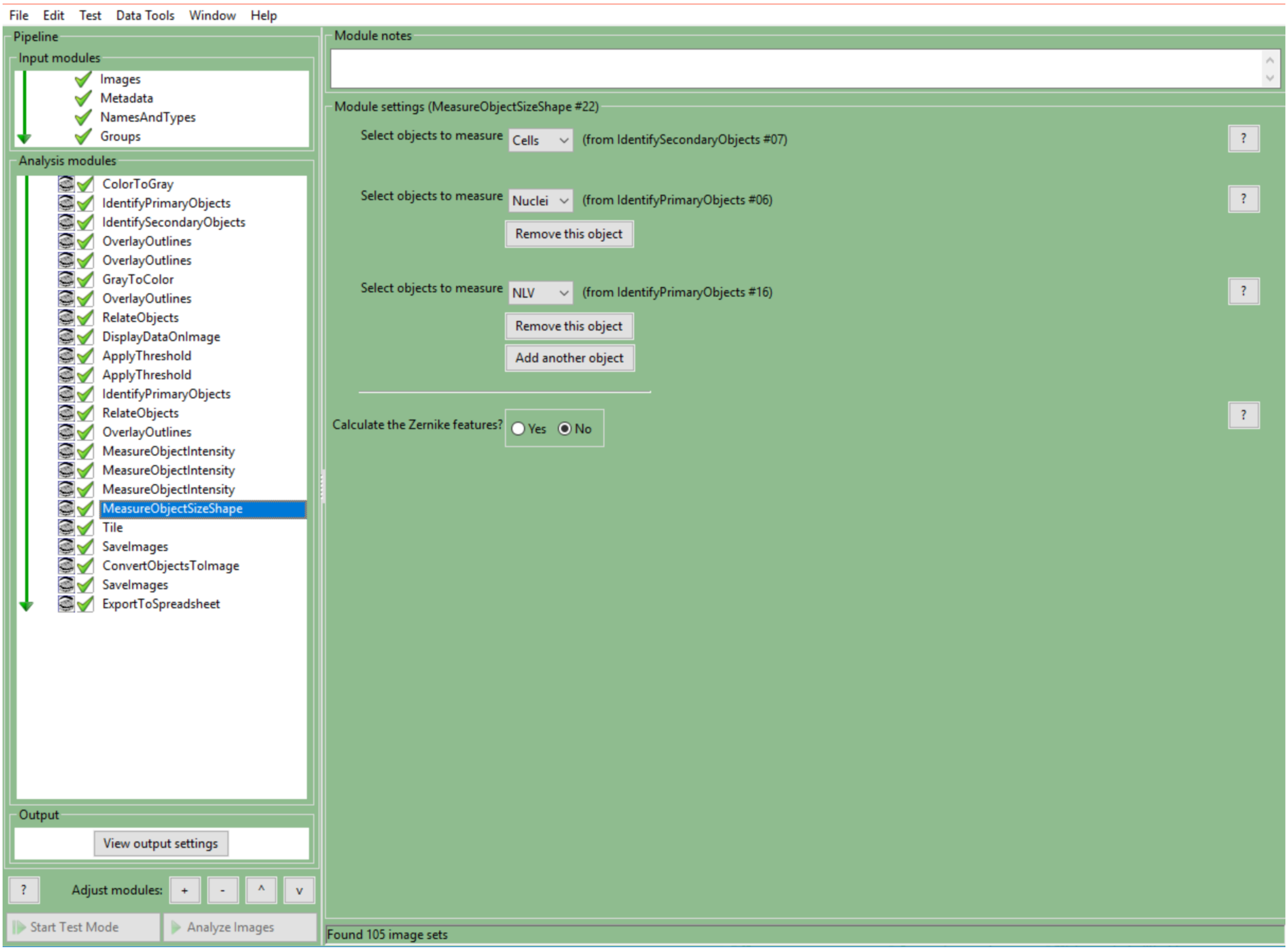
File Edit Test Data Tools Window Help
Pipeline
Input modules
Images
Metadata
NamesAndTypes
Groups
Analysis modules
ColorToGray
IdentifyPrimaryObjects
IdentifySecondaryObjects
OverlayOutlines
OverlayOutlines
GrayToColor
OverlayOutlines
RelateObjects
DisplayDataOnImage
ApplyThreshold
ApplyThreshold
IdentifyPrimaryObjects
RelateObjects
OverlayOutlines
MeasureObjectIntensity
MeasureObjectIntensity
MeasureObjectIntensity
MeasureObjectSizeShape
Tile
SaveImages
ConvertObjectsToImage
SaveImages
ExportToSpreadsheet
Output
View output settings
Adjust modules:
Start Test Mode
Analyze Images
Module notes
Module settings (MeasureObjectSizeShape #22)
Select objects to measure
Cells
(from IdentifySecondaryObjects #07)
Select objects to measure
Nuclei
(from IdentifyPrimaryObjects #06)
Remove this object
Select objects to measure
NLV
(from IdentifyPrimaryObjects #16)
Remove this object
Add another object
Calculate the Zernike features?
Yes
No
Found 105 image sets

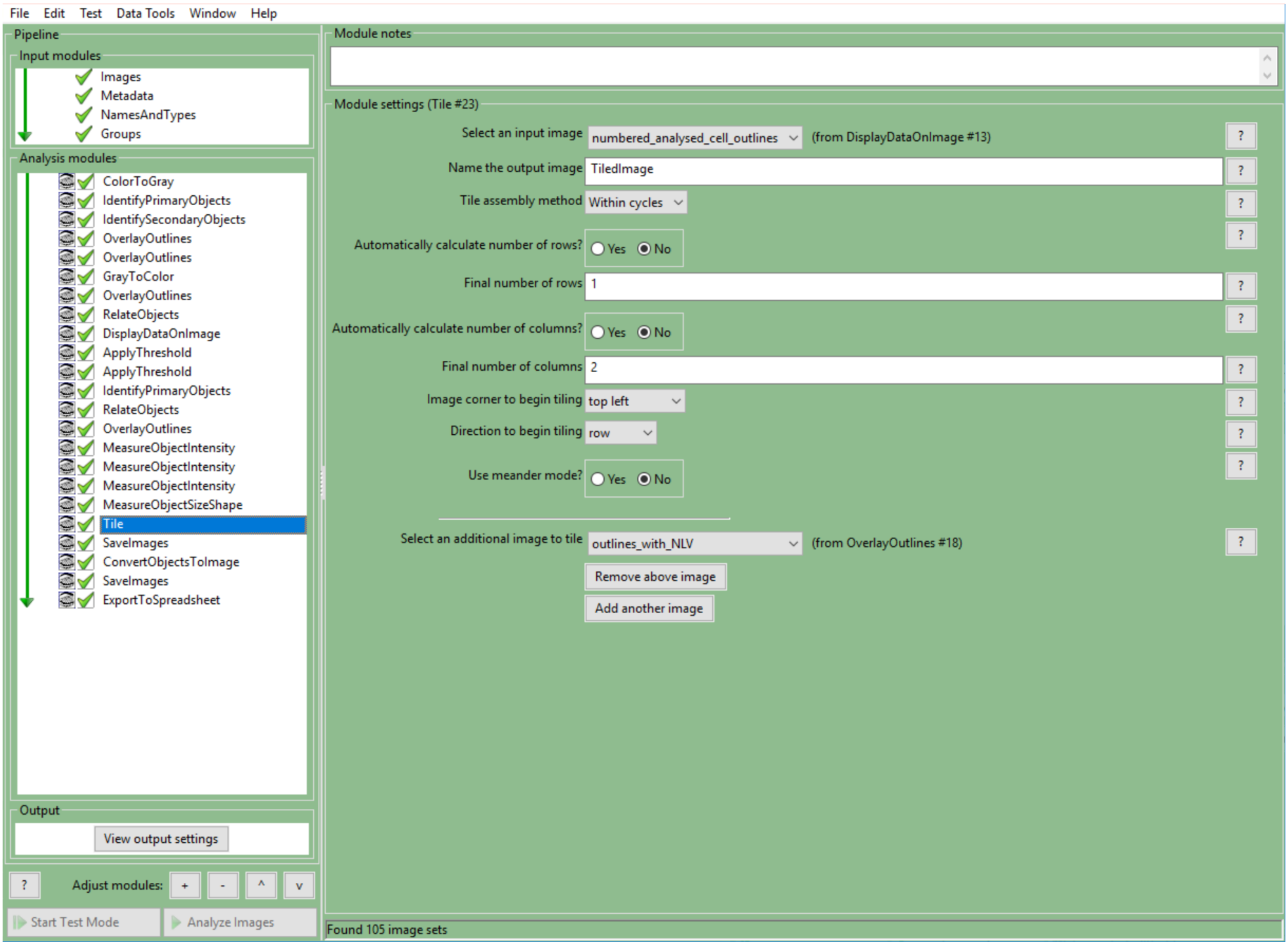
File Edit Test Data Tools Window Help
Pipeline
Input modules
Images
Metadata
NamesAndTypes
Groups
Analysis modules
ColorToGray
IdentifyPrimaryObjects
IdentifySecondaryObjects
OverlayOutlines
OverlayOutlines
GrayToColor
OverlayOutlines
RelateObjects
DisplayDataOnImage
ApplyThreshold
ApplyThreshold
IdentifyPrimaryObjects
RelateObjects
OverlayOutlines
MeasureObjectIntensity
MeasureObjectIntensity
MeasureObjectIntensity
MeasureObjectSizeShape
Tile
SaveImages
ConvertObjectsToImage
SaveImages
ExportToSpreadsheet
Output
View output settings
Adjust modules:
Start Test Mode
Analyze Images
Module notes
Module settings (Tile #23)
Select an input image
numbered_analysed_cell_outlines
(from DisplayDataOnImage #13)
Name the output image
TiledImage
Tile assembly method
Within cycles
Automatically calculate number of rows?
Yes No
Final number of rows
1
Automatically calculate number of columns?
Yes No
Final number of columns
2
Image corner to begin tiling
top left
Direction to begin tiling
row
Use meander mode?
Yes No
Select an additional image to tile
outlines_with_NLV
(from OverlayOutlines #18)
Remove above image
Add another image
Found 105 image sets

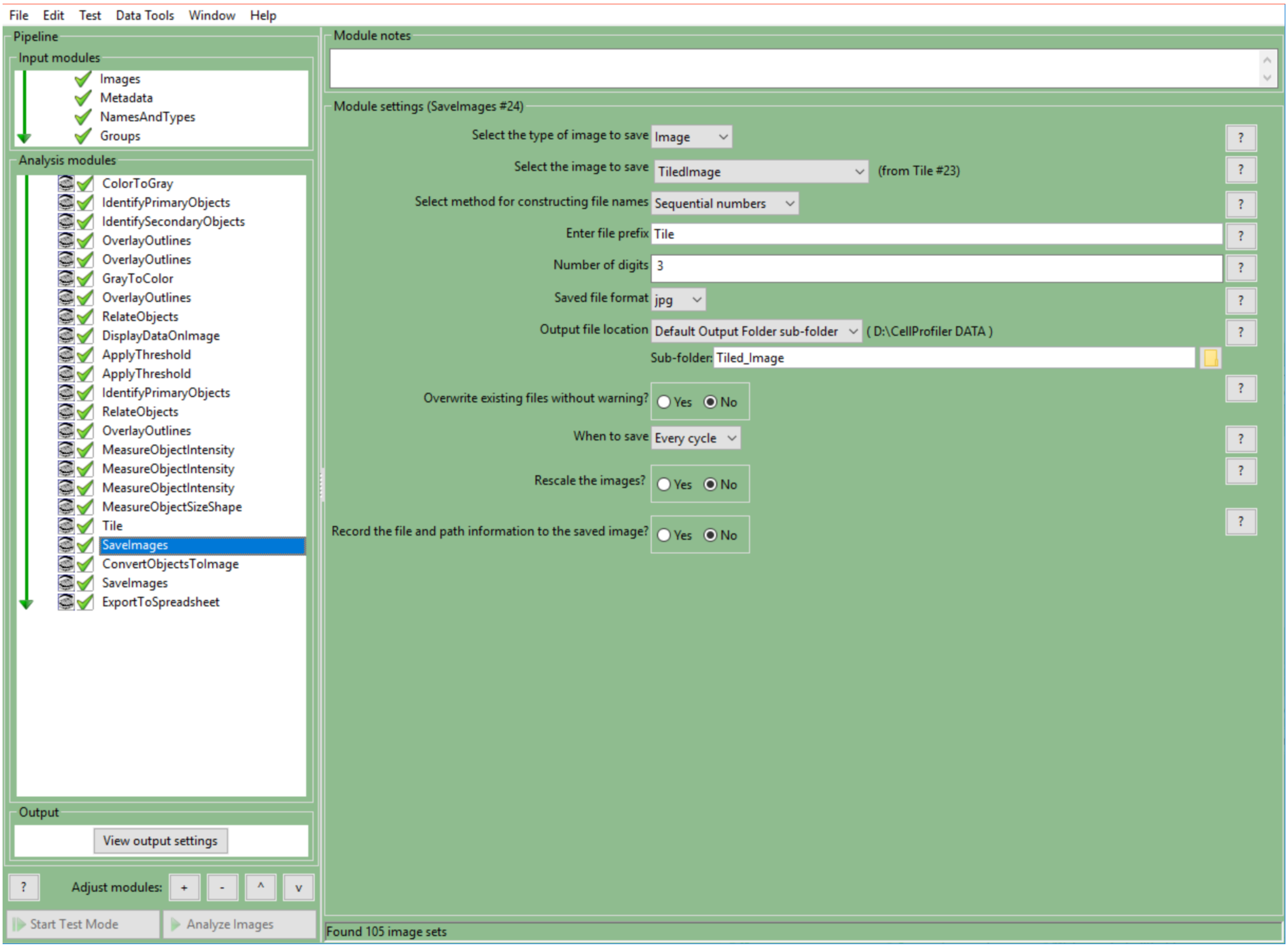
File Edit Test Data Tools Window Help
Pipeline
Input modules
Images
Metadata
NamesAndTypes
Groups
Analysis modules
ColorToGray
IdentifyPrimaryObjects
IdentifySecondaryObjects
OverlayOutlines
OverlayOutlines
GrayToColor
OverlayOutlines
RelateObjects
DisplayDataOnImage
ApplyThreshold
ApplyThreshold
IdentifyPrimaryObjects
RelateObjects
OverlayOutlines
MeasureObjectIntensity
MeasureObjectIntensity
MeasureObjectIntensity
MeasureObjectSizeShape
Tile
SaveImages
ConvertObjectsToImage
SaveImages
ExportToSpreadsheet
Output
View output settings
Adjust modules:
Start Test Mode
Analyze Images
Module notes
Module settings (SaveImages #24)
Select the type of image to save Image
Select the image to save TiledImage (from Tile #23)
Select method for constructing file names Sequential numbers
Enter file prefix Tile
Number of digits 3
Saved file format jpg
Output file location Default Output Folder sub-folder (D:\CellProfiler DATA)
Sub-folder: Tiled_Image
Overwrite existing files without warning? Yes No
When to save Every cycle
Rescale the images? Yes No
Record the file and path information to the saved image? Yes No
Found 105 image sets

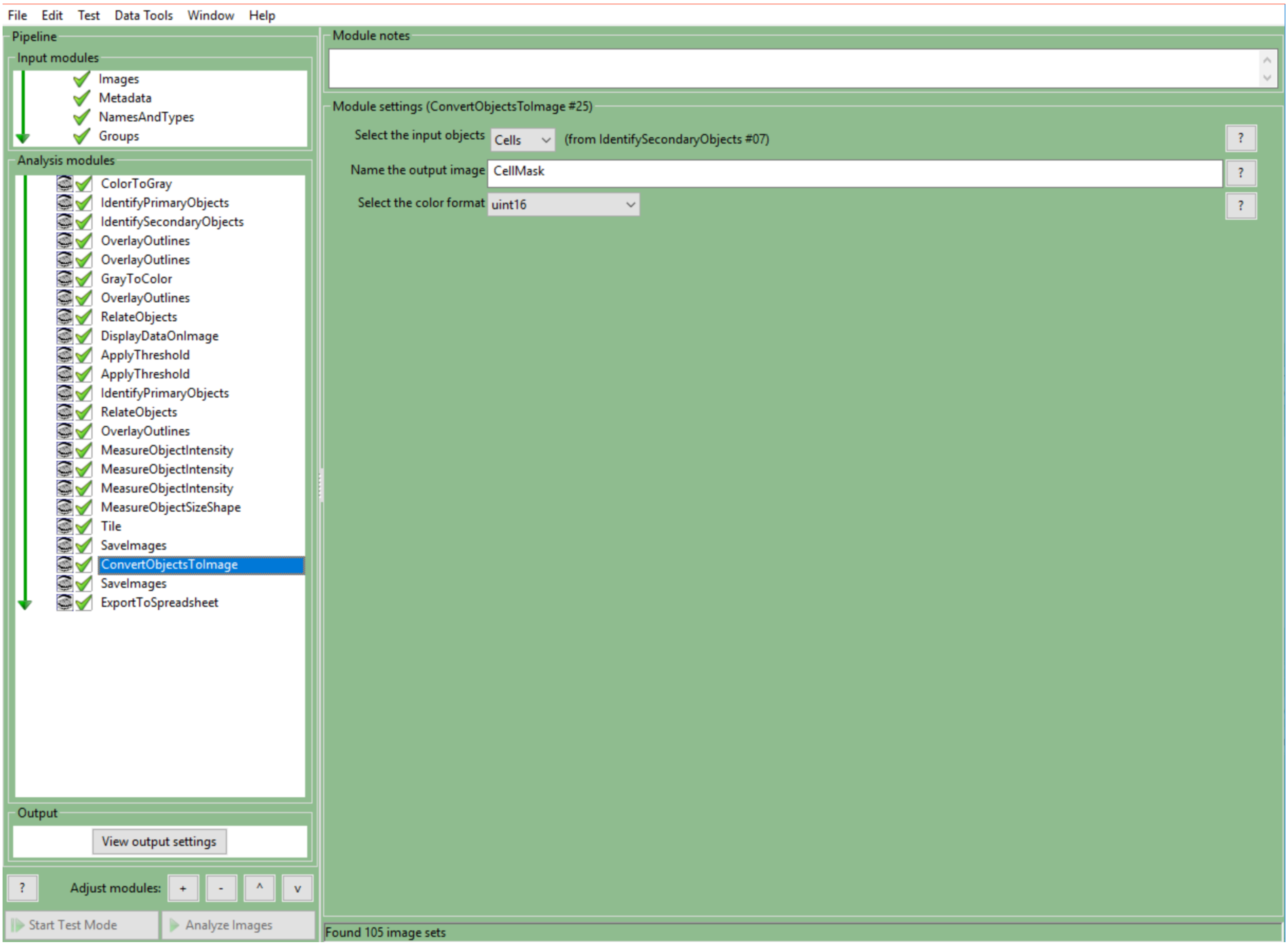
File Edit Test Data Tools Window Help
Pipeline
Input modules
Images
Metadata
NamesAndTypes
Groups
Analysis modules
ColorToGray
IdentifyPrimaryObjects
IdentifySecondaryObjects
OverlayOutlines
OverlayOutlines
GrayToColor
OverlayOutlines
RelateObjects
DisplayDataOnImage
ApplyThreshold
ApplyThreshold
IdentifyPrimaryObjects
RelateObjects
OverlayOutlines
MeasureObjectIntensity
MeasureObjectIntensity
MeasureObjectIntensity
MeasureObjectSizeShape
Tile
SaveImages
ConvertObjectsToImage
SaveImages
ExportToSpreadsheet
Output
View output settings
Adjust modules:
Start Test Mode
Analyze Images
Module notes
Module settings (ConvertObjectsToImage #25)
Select the input objects
Cells
(from IdentifySecondaryObjects #07)
Name the output image
CellMask
Select the color format
uint16
Found 105 image sets

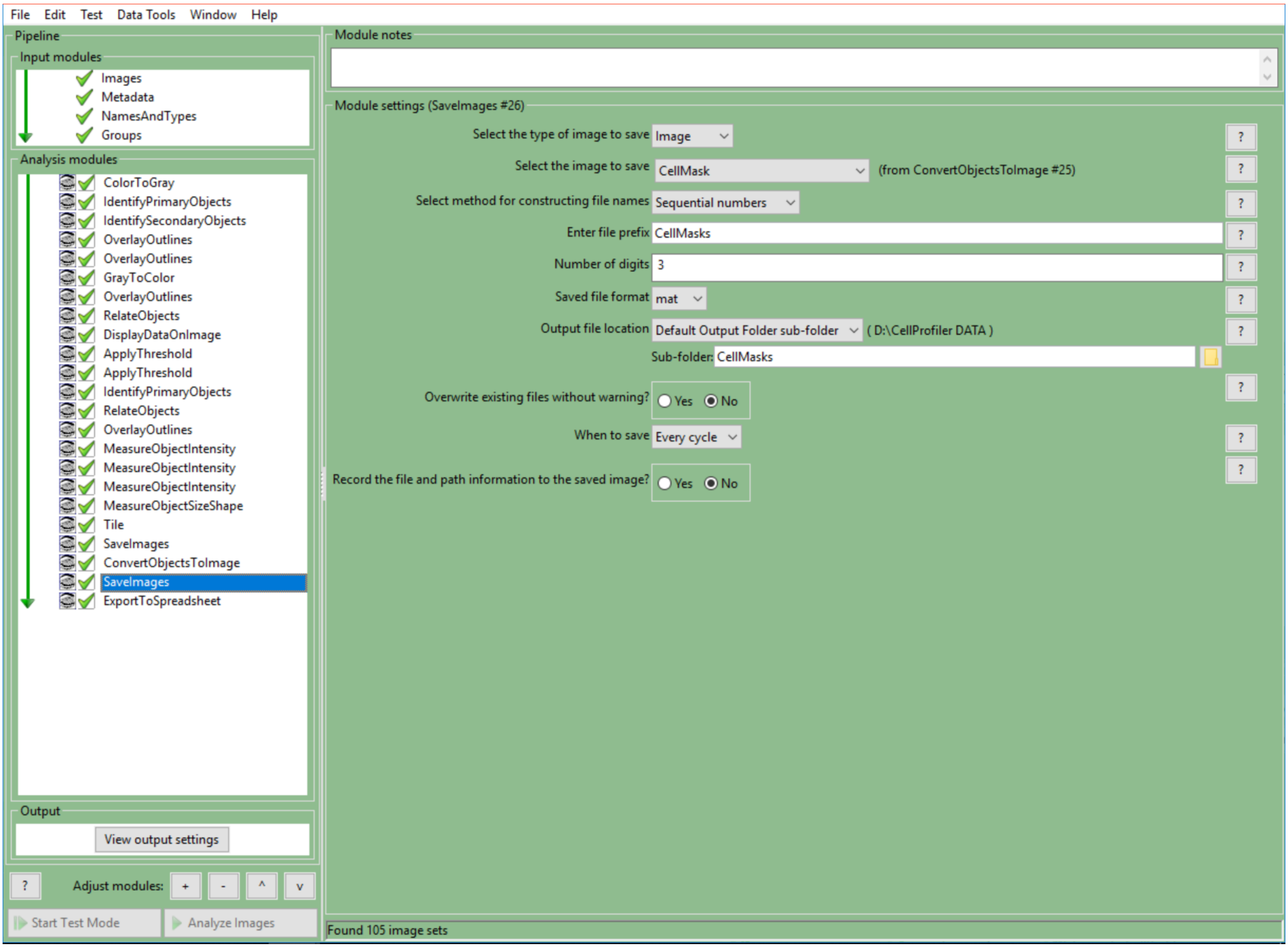
File Edit Test Data Tools Window Help
Pipeline
Input modules
Images
Metadata
NamesAndTypes
Groups
Analysis modules
ColorToGray
IdentifyPrimaryObjects
IdentifySecondaryObjects
OverlayOutlines
OverlayOutlines
GrayToColor
OverlayOutlines
RelateObjects
DisplayDataOnImage
ApplyThreshold
ApplyThreshold
IdentifyPrimaryObjects
RelateObjects
OverlayOutlines
MeasureObjectIntensity
MeasureObjectIntensity
MeasureObjectIntensity
MeasureObjectSizeShape
Tile
SaveImages
ConvertObjectsToImage
SaveImages
ExportToSpreadsheet
Output
View output settings
Adjust modules:
Start Test Mode
Analyze Images
Module notes
Module settings (SaveImages #26)
Select the type of image to save Image
Select the image to save CellMask (from ConvertObjectsToImage #25)
Select method for constructing file names Sequential numbers
Enter file prefix CellMasks
Number of digits 3
Saved file format mat
Output file location Default Output Folder sub-folder (D:\CellProfiler DATA)
Sub-folder: CellMasks
Overwrite existing files without warning? Yes No
When to save Every cycle
Record the file and path information to the saved image? Yes No
Found 105 image sets

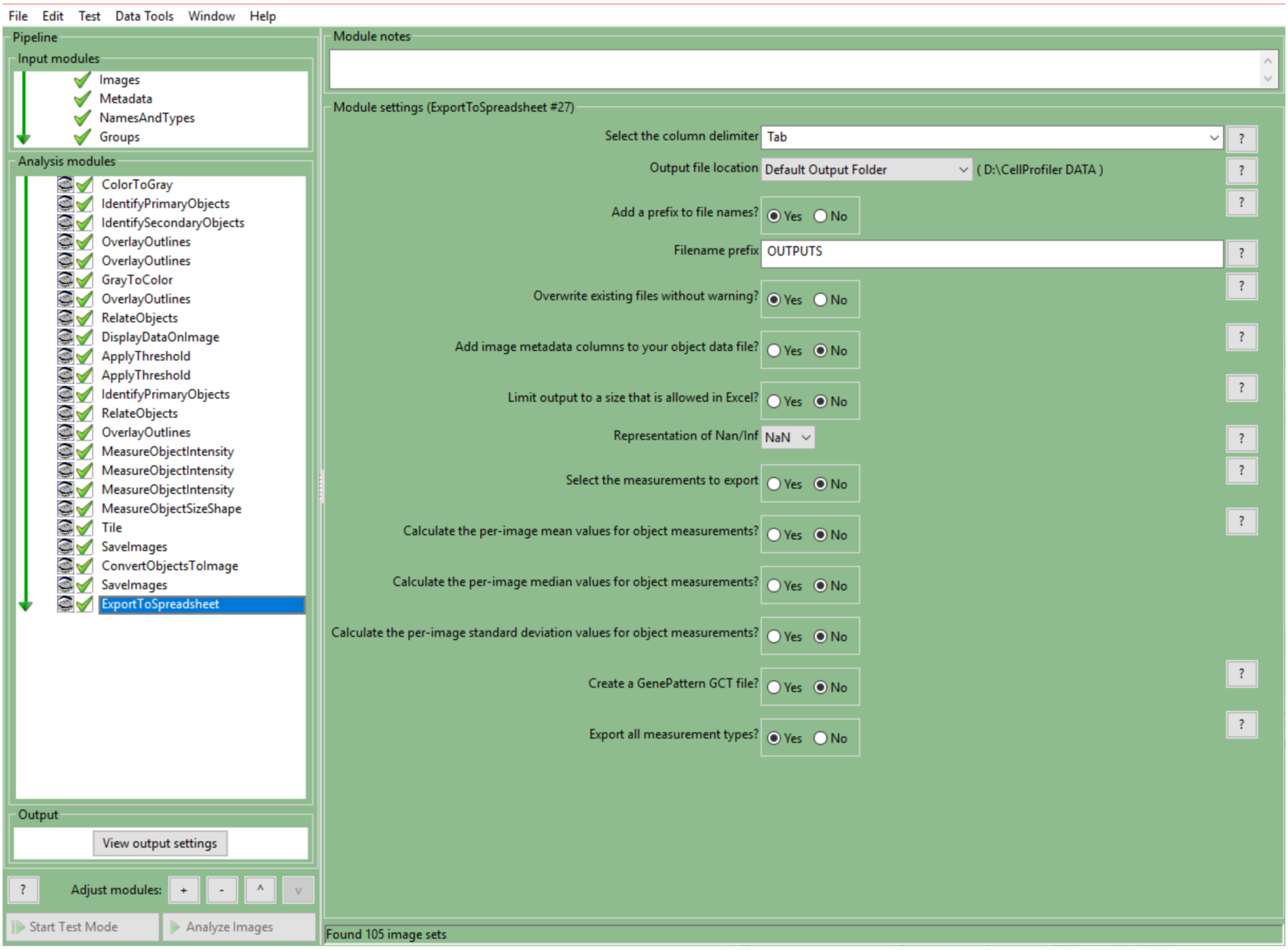
File Edit Test Data Tools Window Help
Pipeline
Input modules
Images
Metadata
NamesAndTypes
Groups
Analysis modules
ColorToGray
IdentifyPrimaryObjects
IdentifySecondaryObjects
OverlayOutlines
OverlayOutlines
GrayToColor
OverlayOutlines
RelateObjects
DisplayDataOnImage
ApplyThreshold
ApplyThreshold
IdentifyPrimaryObjects
RelateObjects
OverlayOutlines
MeasureObjectIntensity
MeasureObjectIntensity
MeasureObjectIntensity
MeasureObjectSizeShape
Tile
SaveImages
ConvertObjectsToImage
SaveImages
ExportToSpreadsheet
Output
View output settings
Adjust modules:
Start Test Mode
Analyze Images
Module notes
Module settings (ExportToSpreadsheet #27)
Select the column delimiter Tab
Output file location Default Output Folder (D:\CellProfiler DATA)
Add a prefix to file names? Yes No
Filename prefix OUTPUTS
Overwrite existing files without warning? Yes No
Add image metadata columns to your object data file? Yes No
Limit output to a size that is allowed in Excel? Yes No
Representation of Nan/Inf NaN
Select the measurements to export Yes No
Calculate the per-image mean values for object measurements? Yes No
Calculate the per-image median values for object measurements? Yes No
Calculate the per-image standard deviation values for object measurements? Yes No
Create a GenePattern GCT file? Yes No
Export all measurement types? Yes No
Found 105 image sets

Supplementary Note 2 – Model parameters and fitting instructions. This section contains a complete list and description of all model parameters alongside dedicated instructions for fitting the nanoparticle uptake model derived herein.

Model parameters required for fitting NLV and area histograms

Symbol	Definition	Use in analysis
A	Area of cell	Cell area is measured for every cell in the population
P(A)	Distribution of area per cell	Determined from the measured values of area.
α	Gamma function fitting parameter	α and β parameters are determined by fitting a gamma function to cell area distribution – *see EXCEL spreadsheet provided in Supplementary Data 2*
β	Gamma function fitting parameter	α and β parameters are determined by fitting a gamma function to cell area distribution – *see EXCEL spreadsheet provided in Supplementary Data 2*
C	Concentration of nanoparticles	Defined by experimental exposure conditions.
t	Time of nanoparticle exposure	Defined by experimental exposure conditions.
N_{NLV}	Number of nanoparticle loaded vesicles per cell	Number of NLV is measured for every cell in the population
$P(N_{NLV})$	Distribution of NLV per cell	Determined from the measured values of N_{NLV}.
r	Negative binomial parameter	Determined from the negative binomial fits to the NLV distribution
p	Negative binomial parameter	Determined from the negative binomial fits to the NLV distribution
λ	Rate of uptake of at least one particle/agglomerate via endocytosis per unit concentration per unit area.	Determined from the negative binomial parameter p or from the mean number of NLV versus concentration-time product – *see analysis pipeline below*

Measured   Experimental Conditions   Fitted

Values of parameters used in the manuscript for fits

	BEAS-2B	A549
Mean α	500.2+/-33.9	280.1+/-24.2
Mean β	3.39+/-0.24	4.23+/-0.28
λ	$0.00107 nM^{-1} hr^{-1} m^{-2}$	$0.00135 nM^{-1} hr^{-1} m^{-2}$

Parameters required to derive model but not required for fitting

Symbol	Definition
N_{end}	Number of endosomes per unit area generated by the cell
N_{arr}	Number of agglomerates arriving at the cell surface during the exposure time
$P(N_{arr})$	Probability of N_{arr} nanoparticle agglomerates arriving at the cell surface during the exposure time.
μ	Endosome generation rate per unit area per unit time
κ	Probablity per unit concentration of a least one particle arriving in the forming endosome.
$\Delta = \kappa C$	Arrival rate of particle clusters in a forming endosome.
$\lambda = \mu\kappa$	Rate of uptake of at least one particle/agglomerate via endocytosis per unit concentration per unit area.
r	Negative binomial parameter
p	Negative binomial parameter

ANALYSIS PIPELINE

Procedure to find the area parameters and NLV distribution

1. Determine the cell area distribution and fit to a gamma function

Use any image analysis package to calculate the area of each cell in the population.

Find the area probability distribution by finding the normalized histogram of the cell areas.

Fit a gamma distribution to the area distribution to determine the gamma function parameters α and β.

See the EXCEL spreadsheet template included in Supplementary Data 2 to perform this analysis.

2. Measure the NLV distribution

Again using image analysis measure the number of fluorescent vesicles (NLV) for each cell in the population.

Find the NLV probability distribution by finding the normalized histogram of the cell NLV counts.

See the EXCEL spreadsheet template included in Supplementary Data 2 to perform this analysis.

Procedure for extracting parameters from NLV distributions

We now have two options to extract parameters from the NLV distributions

3. Determination of the uptake rate λ by fitting NLV distributions

We can directly fit a negative binomial distribution to the measured NLV distributions measured at different dose conditions. This allows us to determine the negative binomial function parameters r and p. From the value of p obtained in this fitting procedure we can invert the equation given in the manuscript

$p=\beta\lambda Ct/(1+\beta\lambda Ct)$

to obtain a value for λ. (as we know C, t and β from the area fitting)

The included EXCEL spreadsheet in Supplementary Data 2 also performs this analysis.

We note this could be extended to simultaneously fit several distributions with the same r and p parameters. (and therefore λ).

4. Determination of the uptake rate λ using mean NLV counts

If we have several dose conditions we are able to plot the mean NLV number per cell versus the concentration-time product as shown in figure 2c/d.

We can fit a straight line to this graph, the slope of which is $\lambda \times \alpha\beta$ as given in equation 14.

As α and β are known from the area so we are able to obtain λ.

Predictability of the model

5. Prediction of NLV distributions and mean NLV number

Once we have derived the NLV uptake parameter λ we are now able to make predictions of the NLV uptake for that cell line for different concentration and times of exposure.

We note the negative binomial distribution parameter $r=\alpha$ can obtained directly from the cell area distribution and $p=\beta\lambda Ct / (1+\beta\lambda Ct)$ can be calculated directly for any concentration, C and exposure time, t.
