# Supplementary material for: The origin of heterogeneous nanoparticle uptake by cells
Source: Nat Commun. 2019 May 28;10:2341. doi: 10.1038/s41467-019-10112-4 (PMC6538724; doi:10.1038/s41467-019-10112-4)
Supplement: Supplementary file 3 — Description of Additional Supplementary Files [file 41467_2019_10112_MOESM3_ESM.docx]

**Description of Supplementary Files**

**File Name:** Supplementary Data 1

**Description:** CellProfiler data analysis pipeline and example image data. This zipped folder contains the CellProfiler image analysis pipeline as a .cpproj file. This can be opened in CellProfiler and used to reproduce the image analysis strategy and subsequent analyses described in this work. Raw image data for the BEAS-2B 0.5nM exposure (DTP = 0.5 nM.Hr) is provided alongside in the Zeiss microscopy .lsm format. This file can be read directly by dragging and dropping it into the file list of the ‘images’ module of the CellProfiler pipeline.

**File Name:** Supplementary Data 2

**Description:** Excel version of the nanoparticle uptake model. This zipped folder contains an excel version of the nanoparticle uptake model alongside detailed instructions of how to use it. This package permits statistical prediction of the distribution of number of NLV or total fluorescence intensity with no dedicated programming required.
