# Supplementary material for: The origin of heterogeneous nanoparticle uptake by cells
Source: Nat Commun. 2019 May 28;10:2341. doi: 10.1038/s41467-019-10112-4 (PMC6538724; doi:10.1038/s41467-019-10112-4)
Supplement: Supplementary file 5 — Supplementary Data 2 [file 41467_2019_10112_MOESM5_ESM.zip › Supplementary Data 2_Rees et al/INSTRUCTIONS.docx]

**Instructions on the use of the excel spreadsheet to fit distributions to cell data**

1. Open the excel spreadsheet (NB: Most of the columns are protected to prevent you from accidentally altering the distribution fitting process, however, **columns B and K** are editable and will allow you to copy and paste your own data into the spreadsheet to be fitted.)
2. Copy and paste data on **cell area** into **column B** of the spreadsheet and data on **NLV** into **column K** of the spreadsheet. You will notice that the various plots will adjust to accommodate the new data, however, for the theoretical plots to be correct you will need to estimate new parameters for the updated datasets.

**Fitting the Gamma distribution**

1. Ensure that you have the **Solver** Add-in, this can be added to your excel by going to **File** in the ribbon at the top of the spreadsheet and clicking on **Options** which will appear in the Green ribbon at the bottom left of the screen.


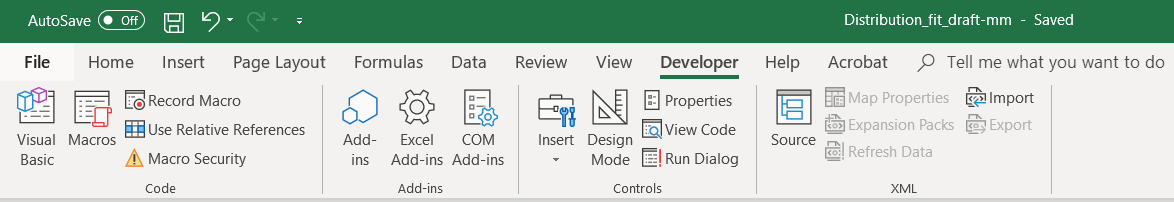

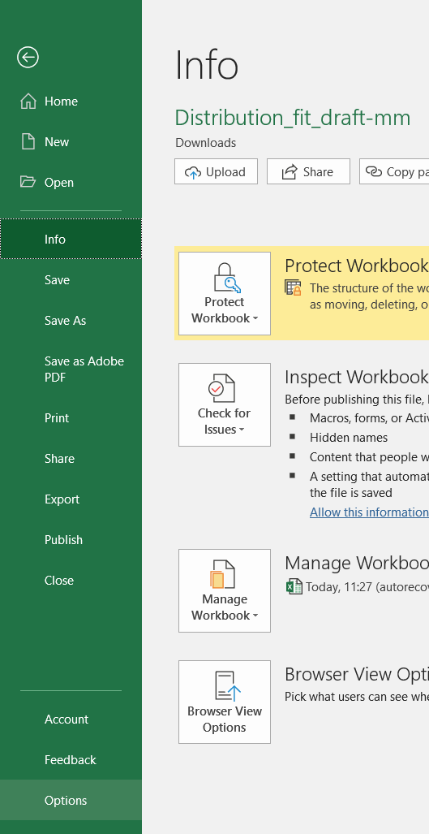


1. A new window titled **Excel options** will appear. Click on **Add-ins** in the left-hand column of the window.


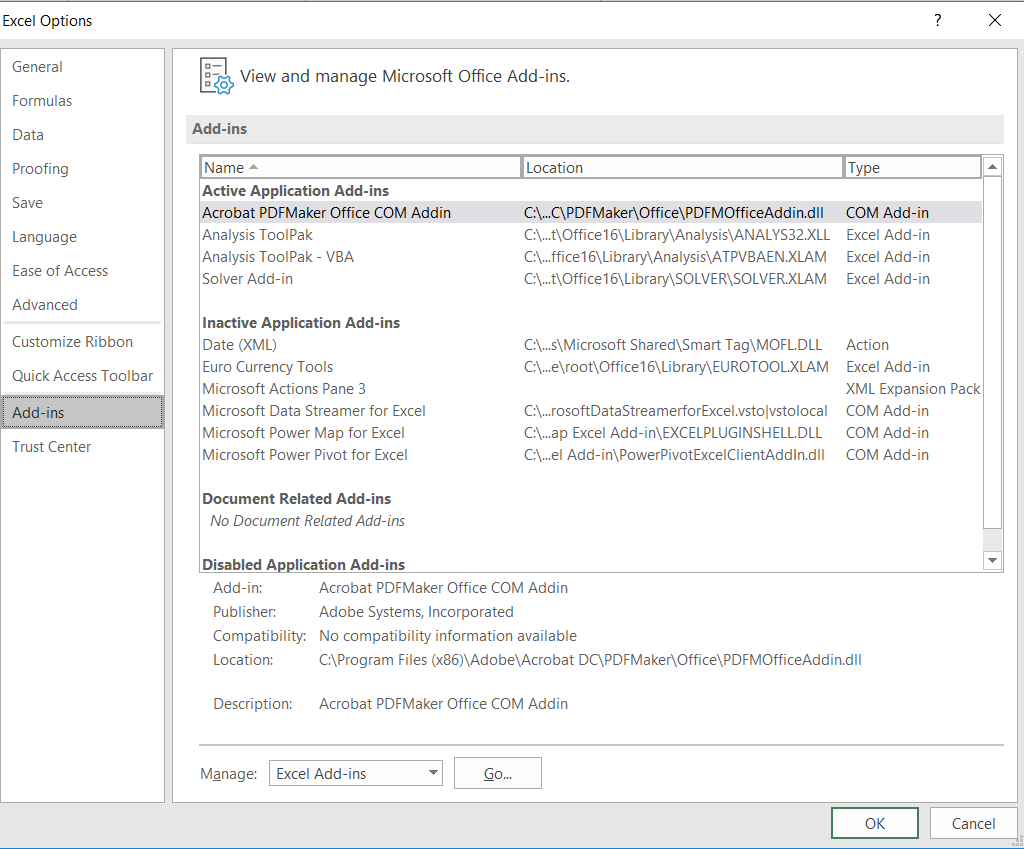


1. A list of **Active application Add-ins** will appear. Click on the **Solver** Add-in which will be last in the list. Click **Go** at the bottom of the window and in the new window that will appear tick **Solver Add-in** and **OK**. Solver has now been added to your excel applications.


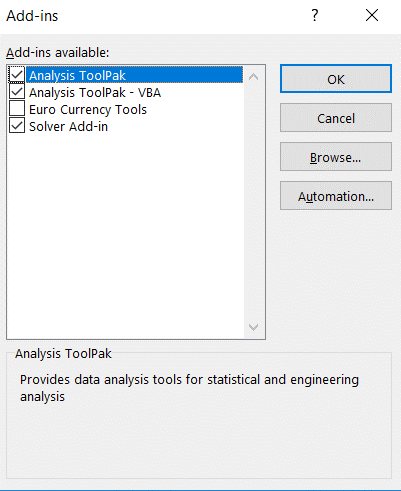


1. In order to fit the **gamma distribution** to the cell area data, copy and paste the initial estimates for alpha and beta (**I 14** and **I 13** respectively) into the parameter boxes (**I 7** and **I** **6**)


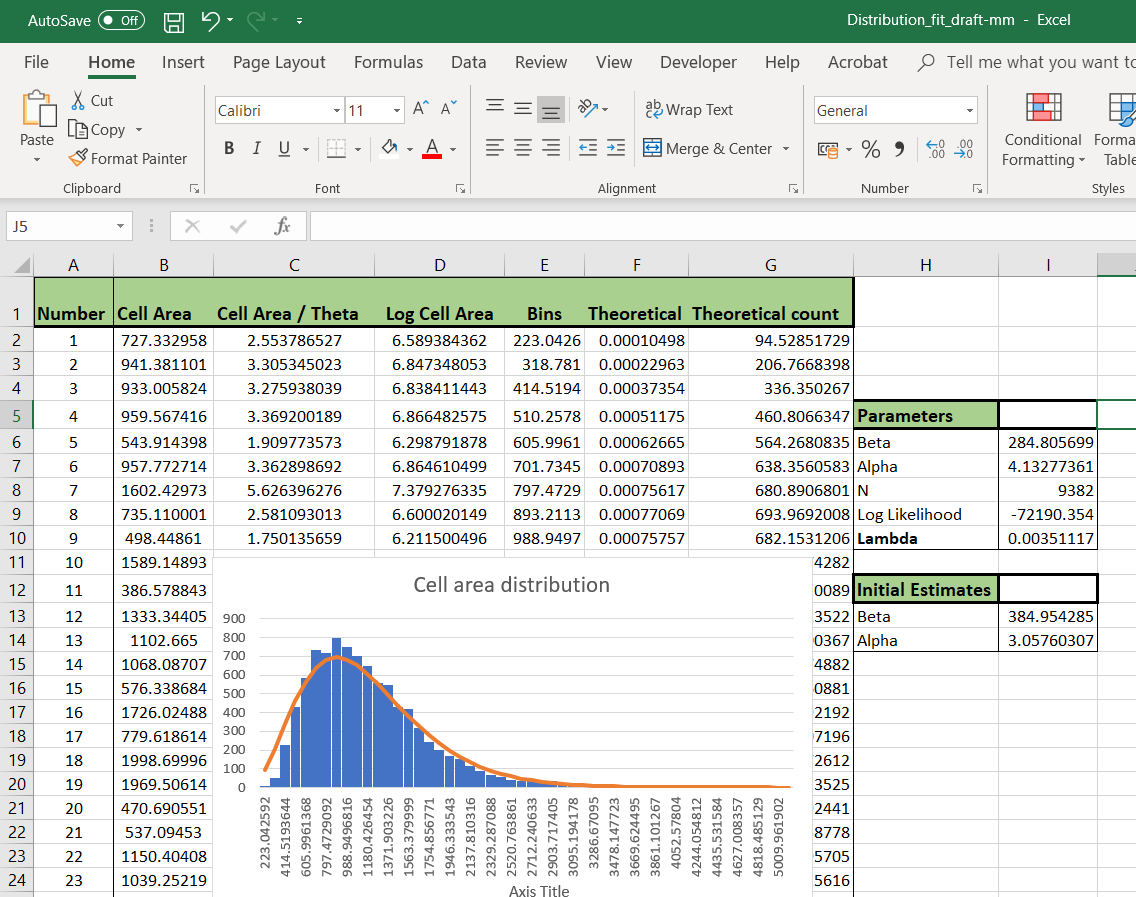


1. Click on the **Data** tab in the ribbon at the top of the spreadsheet and select **Solver** that will appear to the far right of the ribbon


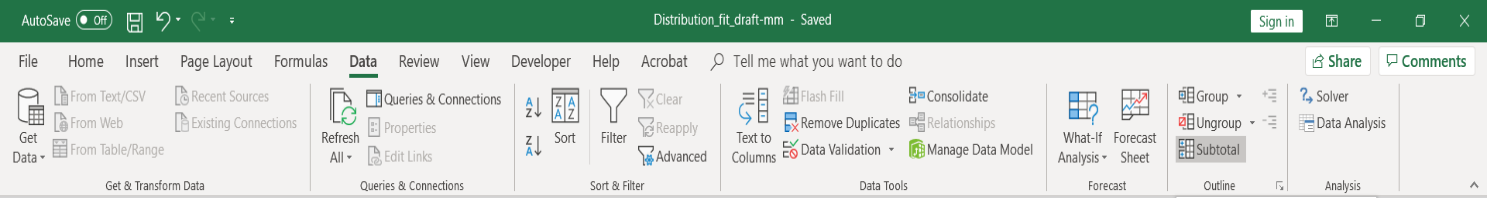


1. The **Solver** Add-in will open up and you should enter the cell you would like to optimise into the **Set Objective** field (In this case it will be the Log Likelihood of the distribution located at **$1$9**). It is also important to make sure you have selected to **Max** this value


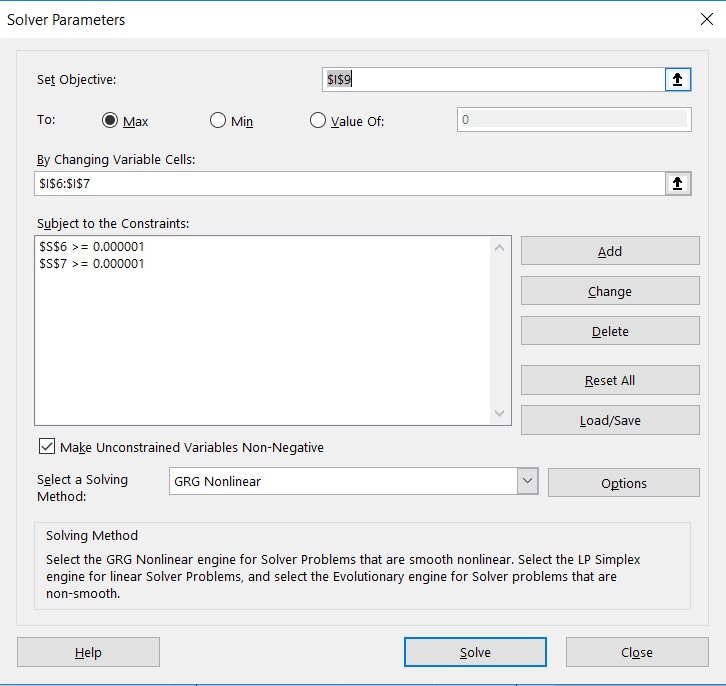


1. Now you need to tell Solver which variables to change (in this case it will be alpha and beta located at **$I$6:I$7)**


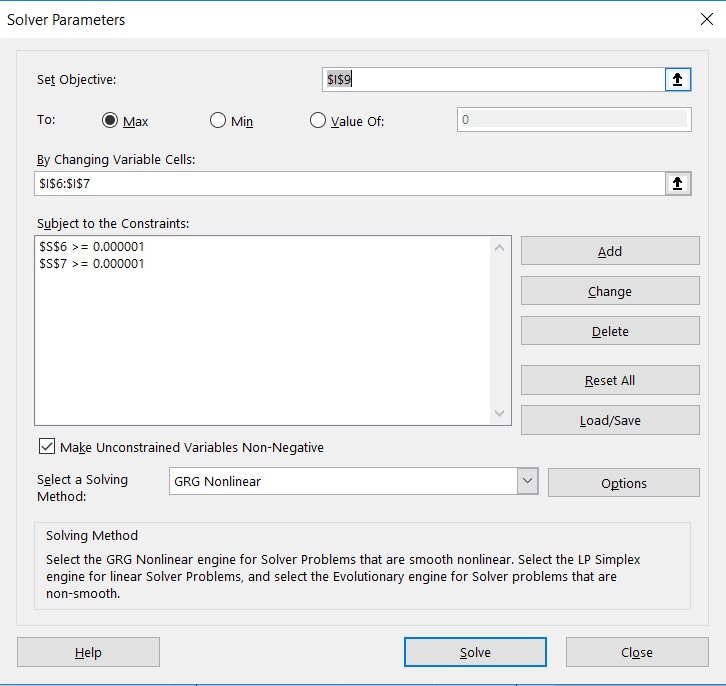


1. You will also need to specify some constraints, to do this click on the **Add** option on the right-hand side of the window. Then ensure to specify that **$I$6 >= 0.000001** and **$I$7 >= 0.000001,** followed by clicking **OK**


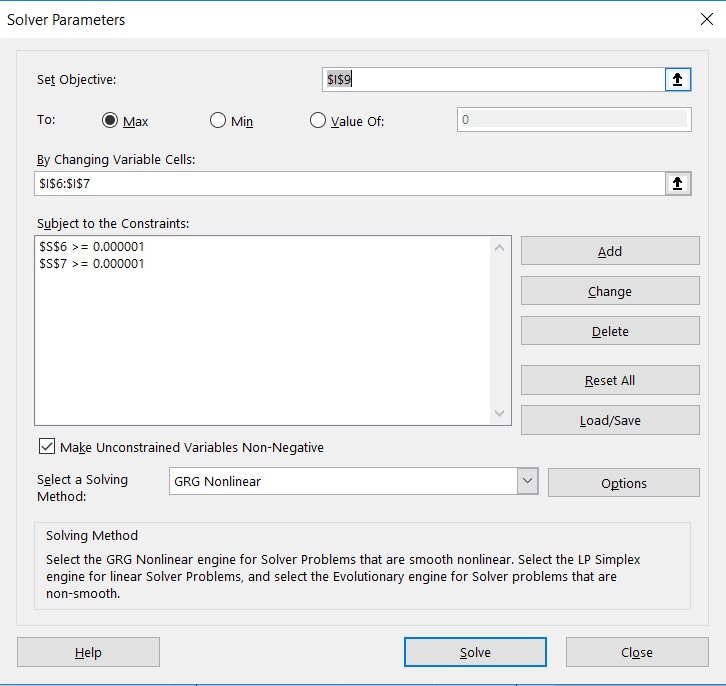

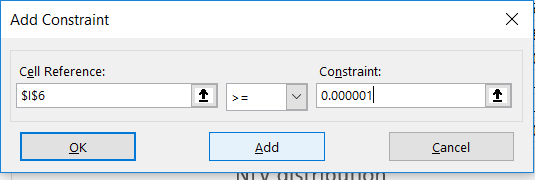


1. Click **Solve** at the bottom of the window once the constraints have been added. You will be told when a solution is reached, if you now look at the parameters located at **I6** and **I7** you will notice that they have been optimised and the plots of the theoretical distribution have been adjusted accordingly


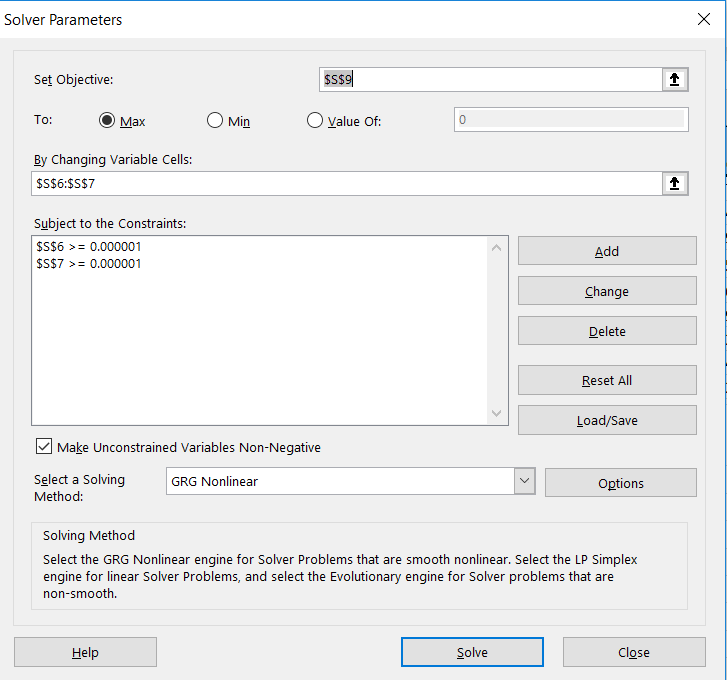

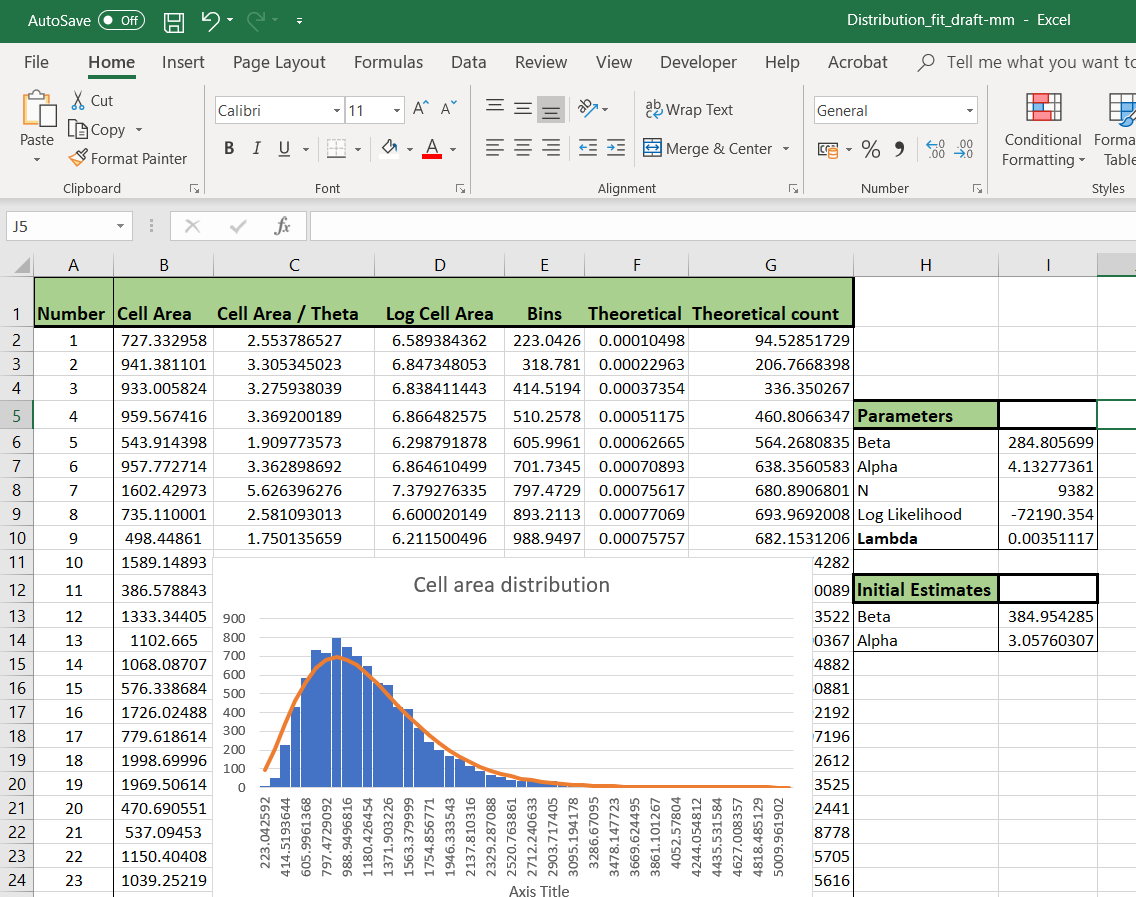


**Fitting the Negative Binomial distribution**

1. In order to fit the negative binomial to the **NLV** data steps 9 – 13 can be repeated. This time to optimise the Log Likelihood at **S9** by changing the variables situated at **S6** and **S7.**
