# Supplementary material for: The origin of heterogeneous nanoparticle uptake by cells
Source: Nat Commun. 2019 May 28;10:2341. doi: 10.1038/s41467-019-10112-4 (PMC6538724; doi:10.1038/s41467-019-10112-4)
Supplement: Supplementary file 6 — Reporting Summary [file 41467_2019_10112_MOESM6_ESM.pdf]

nature research

Corresponding author(s): P. Rees

Last updated by author(s): Apr 4, 2019

Reporting Summary

Nature Research wishes to improve the reproducibility of the work that we publish. This form provides structure for consistency and transparency in reporting. For further information on Nature Research policies, see Authors & Referees and the Editorial Policy Checklist.

Statistics

For all statistical analyses, confirm that the following items are present in the figure legend, table legend, main text, or Methods section.

n/a	Confirmed	
☐	☒	The exact sample size (*n*) for each experimental group/condition, given as a discrete number and unit of measurement
☐	☒	A statement on whether measurements were taken from distinct samples or whether the same sample was measured repeatedly
☐	☒	The statistical test(s) used AND whether they are one- or two-sided<br>*Only common tests should be described solely by name; describe more complex techniques in the Methods section.*
☒	☐	A description of all covariates tested
☒	☐	A description of any assumptions or corrections, such as tests of normality and adjustment for multiple comparisons
☐	☒	A full description of the statistical parameters including central tendency (e.g. means) or other basic estimates (e.g. regression coefficient) AND variation (e.g. standard deviation) or associated estimates of uncertainty (e.g. confidence intervals)
☐	☒	For null hypothesis testing, the test statistic (e.g. F, t, r) with confidence intervals, effect sizes, degrees of freedom and P value noted<br>*Give P values as exact values whenever suitable.*
☒	☐	For Bayesian analysis, information on the choice of priors and Markov chain Monte Carlo settings
☒	☐	For hierarchical and complex designs, identification of the appropriate level for tests and full reporting of outcomes
☒	☐	Estimates of effect sizes (e.g. Cohen's d, Pearson's r), indicating how they were calculated

Our web collection on statistics for biologists contains articles on many of the points above.

Software and code

Policy information about availability of computer code

Data collection	Image data was acquired and stored using the Zeiss .lsm file format. Image analysis was carried out directly from these raw files using the open source CellProfiler software (version 2.2.0). The complete CellProfiler image analysis pipeline and all raw confocal data is provided alongside instructions for running in the BioStudies database under the accession code S-BSST249, allowing full reproduction of our image analysis strategy.
Data analysis	Data analysis was carried out using MATLAB R2018a. A complete, step-wise derivation of our statistical model is presented in the Methods. The MATLAB code needed to reproduce all analyses shown in the final version of the figures alongside instructions for running is deposited in the BioStudies database under the accession code S-BSST249.

For manuscripts utilizing custom algorithms or software that are central to the research but not yet described in published literature, software must be made available to editors/reviewers. We strongly encourage code deposition in a community repository (e.g. GitHub). See the Nature Research guidelines for submitting code & software for further information.

Data

Policy information about availability of data

All manuscripts must include a data availability statement. This statement should provide the following information, where applicable:

- Accession codes, unique identifiers, or web links for publicly available datasets
- A list of figures that have associated raw data
- A description of any restrictions on data availability

28GB of raw confocal data, the CellProfiler pipeline, all MATLAB code needed to reproduce the results and all of the Figures, as well as the measurement outputs of the CellProfiler pipeline are provided in the BioStudies database under the accession code S-BSST249 (www.ebi.ac.uk/biostudies/studies/S-BSST249). A full Data and Code availability statement is included in the manuscript to include the direct accession number. Every Figure legend states that the full data and code are available in the BioStudies database under the accession code S-BSST249.

Field-specific reporting

Please select the one below that is the best fit for your research. If you are not sure, read the appropriate sections before making your selection.

☒ Life sciences ☐ Behavioural & social sciences ☐ Ecological, evolutionary & environmental sciences

For a reference copy of the document with all sections, see nature.com/documents/nr-reporting-summary-flat.pdf

Life sciences study design

All studies must disclose on these points even when the disclosure is negative.

Sample size	Sample sizes were determined by the number of cells and constituent number of nanoparticle-loaded vesicles imaged across all fields-of-view per dose/time exposure combination. The nature of our high throughput imaging approach allowed statistically defensible populations containing more than 10^4 cells or 10^5 NLV to be analysed for each dose / exposure duration.
Data exclusions	As is standard practice for image based cell profiling, cell-objects with areas outside of the 5th-to-95% percentile were discarded prior to analysis to minimise the impact of any mis-segmented cells on the results obtained. Similarly, any cell-objects partially obscured due to overlapping the edges of the imaging field-of-view were also discarded to ensure per-cell nanoparticle delivery was accurately quantified.
Replication	The presented statistical model describing the delivery of nanoparticles to cells was replicated across five different dose-time exposure combinations - spanning a 16-fold range - in two different cell lines. All results are presented in full and the concordance observed across all results was excellent.
Randomization	Flasks of cells were dosed with nanoparticles in a randomised order. Similarly, image-sets for each dose / exposure combination were collected in a random order.
Blinding	Slides were blinded prior to image-set collection.

Reporting for specific materials, systems and methods

We require information from authors about some types of materials, experimental systems and methods used in many studies. Here, indicate whether each material, system or method listed is relevant to your study. If you are not sure if a list item applies to your research, read the appropriate section before selecting a response.

Materials & experimental systems

n/a	Involved in the study	
☒	☐	Antibodies
☐	☒	Eukaryotic cell lines
☒	☐	Palaeontology
☒	☐	Animals and other organisms
☒	☐	Human research participants
☒	☐	Clinical data

Methods

n/a	Involved in the study	
☒	☐	ChIP-seq
☒	☐	Flow cytometry
☒	☐	MRI-based neuroimaging

Eukaryotic cell lines

Policy information about cell lines

Cell line source(s)	Normal bronchial (BEAS-2B) and lung carcinoma (A549) cells lines were purchased from ATCC® (product numbers #CRL-9609 and #CLL-185 respectively).
Authentication	As the cell lines were purchased directly from the supplier for the study, and because the exact cell type used during the study was of minimal relevance (i.e., we provide a universal model of nanoparticle delivery - regardless of cell type), further authentication beyond that of ATCC was not carried out.
Mycoplasma contamination	Both cell lines were tested for Mycoplasma using MycoAlert® mycoplasma detection kit (Lonza, Slough, UK).
Commonly misidentified lines (See ICLAC register)	none known.
